# Cognitive Training for Visuospatial Processing in Children Aged 5½ to 6 Years Born Very Preterm With Working Memory Dysfunction: A Randomized Clinical Trial

**DOI:** 10.1001/jamanetworkopen.2023.31988

**Published:** 2023-09-07

**Authors:** Catherine Gire, Any Beltran Anzola, Stéphane Marret, Laurence Foix L’Hélias, Jean-Christophe Roze, Michèle Granier, Hugues Patural, Bénédicte Lecomte, Bernard Guillois, Isabelle Souksi Medioni, Nathalie Bednarek Weirauch, Olivier Claris, Jean-Michel Hascoët, Pierre Kuhn, Meriem Zahed, Mohamed Boucekine, Pierre-Yves Ancel, Catherine Arnaud, Gilles Cambonie, Valérie Dorriere Datin

**Affiliations:** 1 Department of Neonatology, North Hospital, Assistance Publique–Hôpitaux de Marseille University Hospital, Marseille, France; 2 Faculty of Medicine, Centre for Research on Health Services and Quality of Life–Health Service Research and Quality of Life Center, Aix-Marseille University, Marseille, France; 3 Department of Neonatal Pediatrics & Intensive Care, University Hospital of Rouen, Rouen, France; 4 Department of Neonatology, Armand Trousseau Hospital, Assistance Publique–Hôpitaux de Paris, Paris, France; 5 Department of Neonatal & Pediatric Intensive Care, University Hospital of Nantes, Nantes, France; 6 Department of Neonatology, Sud Francilien Hospital, Corbeil-Essonnes, France; 7 Department of Neonatal & Pediatric Intensive Care, University Hospital of Saint-Etienne, Saint-Etienne, France; 8 Department of Neonatal & Pediatric Intensive Care, University Hospital of Clermont-Ferrand, Clermont-Ferrand, France; 9 Department of Neonatal & Intensive Care, University Hospital of Caen Normandie, Caen, France; 10 Department of Neonatal Pediatrics, University Hospital of Nîmes, Nîmes, France; 11 Department of Neonatal Medicine & Pediatric Intensive Care, University Hospital of Reims, Reims, France; 12 Department of Neonatology & Neonatal Intensive Care, University Hospital of Lyon, Lyon, France; 13 Department of Neonatology, Regional University Hospital of Nancy, Nancy, France; 14 Department of Neonatology, Regional University Hospital of Strasbourg, Strasbourg, France; 15 Obstetrical, Perinatal, and Pediatric Epidemiology Team, Center of Research in Epidemiology and Statistics, Paris University, INSERM, Paris, France; 16 Clinical Research Unit, Center for Clinical Investigation, CHU Cochin Broca Hôtel-Dieu, Paris, France; 17 Clinical Epidemiology Unit, University Hospital Toulouse, Toulouse, France; 18 Center for Epidemiology and Research in Population Health, University of Toulouse, INSERM, Paul Sabatier University, Toulouse, France; 19 Department of Neonatal Pediatrics and Intensive Care, University Hospital of Montpellier, Montpellier, France

## Abstract

**Importance:**

Compared with term-born peers, children born very preterm generally perform poorly in executive functions, particularly in working memory and inhibition. By taking advantage of neuroplasticity, computerized cognitive training of working memory in those children could improve visuospatial processing by boosting visual inhibition via working memory.

**Objective:**

To evaluate the long-term effect of cognitive working memory training on visuospatial processing in children aged 5½ to 6 years born very preterm who have working memory impairment.

**Design, Setting, and Participants:**

This multicenter (18 French university hospitals), open-label randomized clinical trial with 2 parallel groups (EPIREMED) was conducted from November 2016 to April 2018, with the last follow-up during August 2019. Eligible children from the EPIPAGE 2 cohort were aged 5½ to 6 years, were born between 24 and 34 weeks’ gestation, and had a global intelligence quotient greater than 70 and a working memory index less than 85. Data were analyzed from February to December 2020.

**Intervention:**

Children were randomized 1:1 to standard care management and a working memory cognitive training program (Cogmed software) for 8 weeks (25 sessions) (intervention) or to standard management (control).

**Main Outcomes and Measures:**

The primary outcome was the visuospatial index score from the Wechsler Preschool and Primary Scale of Intelligence, 4th Edition. Secondary outcomes were working memory, intellectual functioning, executive and attention processes, language skills, behavior, quality of life, and schooling. Neurobehavioral assessments were performed at inclusion and after finishing training at 6 months (intermeditate assessment; secondary outcomes) and at 16 months (final assessment; primary outcome).

**Results:**

There were 169 children randomized, with a mean (SD) age of 5 years 11 months (2 months); 91 (54%) were female. Of the participants, 84 were in the intervention group (57 of whom [68%] completed at least 15 cognitive training sessions) and 85 were in the control group. The posttraining visuospatial index score was not different between groups at a mean (SD) of 3.0 (1.8) months (difference, −0.6 points; 95% CI, −4.7 to 3.5 points) or 12.9 (2.6) months (difference, 0.1 points; 95% CI, −5.4 to 5.1 points). The working memory index score in the intervention group significantly improved from baseline at the intermediate time point (difference, 4.7 points; 95% CI, 1.2-8.1 points), but this improvement was not maintained at the final assessment.

**Conclusions and Relevance:**

This randomized clinical trial found no lasting effect of a cognitive training program on visuospatial processing in children aged 5½ to 6 years with working memory disorders who were born very preterm. The findings suggest that this training has limited long-term benefits for improving executive function. Transient benefits seemed to be associated with the developmental state of executive functions.

**Trial Registration:**

ClinicalTrials.gov Identifier: NCT02757794

## Key Points

**Question:**

Does cognitive training for working memory in children born very preterm improve visuospatial processing?

**Findings:**

In this multicenter, open label randomized clinical trial of 169 children aged 5½ to 6 years who were born very preterm, a cognitive training program showed no long-term efficacy for visuospatial processing.

**Meaning:**

The study found that a cognitive training program had no benefit for improving visuospatial processing among children born very preterm who had working memory disorders.

## Teaser

This randomized clinical trial evaluates whether cognitive working memory training improves visuospatial processing in children born very preterm who have working memory impairment.

## Introduction

Children born very preterm may have neurobehavioral problems that impact school, family, social adjustments, and their adult lives.^1,2^ Neurodevelopmental outcomes in these children are characterized by a set of developmental dysfunctions (eg, language, praxis, executive function [EF], attention, and behavioral disorders) that tend to accumulate and even potentiate each other.^3,4,5,6^

The EFs are high-level cognitive operations fundamental in learning and social adjustments.^7,8^ Executive dysfunctions are central to phenotype development of children born preterm from a cognitive, behavioral, or social point of view.^6,9,10,11,12,13^ The possibility of training and strengthening EFs to optimize overall executive functioning and promote neurodevelopment has been explored in numerous preschool studies and throughout childhood and adolescence.^14,15^ Different modalities have been proposed, ranging from generalist school-based interventions to targeted interventions, such as mindfulness, sports, music, or cognitive training.^16^ A recent systematic review examining the association of cognitive training with preschoolers’ EF that did not include studies on children born very preterm (977 children receiving training vs 1060 controls) showed training programs were significantly more effective for children with developmental difficulties (eg, attention-deficit/hyperactivity disorder) or low socioeconomic status than for typically developing children not of low socioeconomic status.^15^

Specific computerized cognitive training programs supporting EFs have primarily focused on working memory (WM) training with a cognitive training program (Cogmed) or a general approach to training all EFs (Brian; BrainGame).^17^ These cognitive training programs are associated with improved individual WM performance, with a time-limited effect, but not with changes in other untrained EFs or language and visuospatial processing. Additionally, the outcomes of a cognitive training program could be modified depending on the child’s age at testing.^15^ Visuospatial WM cognitive training program methods may suit preschool groups, whose WM development is uniquely visuospatial, thus taking advantage of the neuroplasticity period,^15^ the central executive control system, and the phonological loop’s later development. To date, 2 studies involving preschool-age children and 1 study of adolescents (<150 individuals) born at very low birth weight (<1500 g) showed improved WM at 5 months after a cognitive training program, without knowing whether the results were maintained over time or transferred to other untrained EFs linked to WM.^18,19,20,21^ Moreover, a large randomized clinical trial of a cognitive training program in school-aged children born preterm showed no long-term effects.^22^

Finally, children born very preterm are at risk for global or local visual processing difficulties, which appear to be partly mediated by visual inhibition disorders.^23,24^ One large study of a cognitive training program^25^ showed an improvement in visual inhibition within an adolescent population that underwent surgery for congenital heart disease, with neurobehavioral sequelae similar to those of children born prematurely.^25^

Our study assessed both the ability of a computerized cognitive training program for WM to improve long-term visuospatial abilities in children born very preterm and its effect on overall executive functions. If successful, this study could facilitate a preventative cognitive impairment strategy in children born very preterm.

## Methods

### Study Design

EPIREMED (NCT02757794) is a multicenter (18 units within French university hospitals), randomized (1:1 allocation ratio), and controlled open-label clinical trial with 2 parallel groups: (1) a control group receiving standard care management and (2) an intervention group receiving standard care management and a cognitive training program (Cogmed JM). The EPIREMED trial was approved by the French Ethics Committee and the French National Agency for Medicines and Health Products Safety. Obligatory written informed consent from a parent or legal guardian was obtained for each participant. This study followed the Consolidated Standards of Reporting Trials (CONSORT) reporting guideline.

A detailed description of the EPIREMED trial protocol has been previously published.^26^ The original, final protocol, and summary of amendments are available in Supplement 1. There were no design changes made after beginning the study. A description of the EPIPAGE 2 cohort, used to identify eligible participants, is available in the EPIREMED protocol (Supplement 1) and previous EPIPAGE 2 publications.^1,27,28,29^

### Participants

Participants selected from the EPIPAGE 2 cohort^1^ had the following eligibility criteria: (1) birth between 24 and 34 weeks’ gestation and completion of EPIPAGE 2 assessments at 5½ years of age,^1^ (2) full-scale intelligence quotient (FSIQ) greater than 70, and (3) WM impairment defined by a WM index score of 85 or lower. Enrollment began November 2016 and ended April 2018, with the last follow-up during August 2019. The study included 3 cognitive assessments performed by a neuropsychologist at each inclusion center who was not blinded to the allocation groups: between ages 5½ and 6 years (inclusion visit) and after finishing training at 6 months, between ages 6 and 7 years (intermediate visit), and at 16 months, between ages 7 and 7½ years (final visit).

### Interventions

#### Intervention Group

Two neuropsychologists trained in the computerized cognitive training program (developed for children aged 4-7 years) explained the process to the parent(s) or legal guardian(s) and provided WM and software information sheets. The professional scheduled and designed the structure of the sessions: 3 weekly 15-minute sessions for 8 weeks. Sessions were conducted at home or at the inclusion center.

The child, accompanied by an adult, completed a series of interactive activities automatically adapted for each individual. After each session, the neuropsychologist checked the adherence and results online. The program calculated a performance score representing the difference between the maximum and starting levels and offered analysis of the child’s progress compared with their baseline.^30^ The parents participated in a weekly 30-minute discussion with the neuropsychologist.

#### Control Group

No rehabilitation program was conducted in the control group. The control group received customary care management with speech therapy and/or academic support for children experiencing academic difficulties.

### Outcomes

The primary outcome was the visuospatial index (VSI) score from the Wechsler Preschool and Primary Scale of Intelligence, 4th Edition (WPPSI-IV).^31^ Secondary outcomes were working memory, intellectual functioning, executive and attention processes, language skills assessed by the Communiquer, Lire et Ecrire Pour Apprendre^32^ battery (including pragmatic and semantic aspects), behavior, quality of life, and schooling. The specific outcome assessment instruments and collected measures are described in eTable 1 in Supplement 2.

### Sample Size

A sample size of 64 participants per group (128 total) was selected to obtain 80% power to detect a mean (SD) difference of 7.5 (0.5) points on the VSI at 16 months after training between the 2 groups and was clinically significant compared with similar studies.^22,33^ The threshold for statistical significance was 2-sided *P* = .05. We assumed 166 patients were needed (83 per group), projecting that 23% of patients would be lost to follow-up between baseline and their last assessment.

### Randomization

A secure, computer-generated clinical research platform was used for participant randomization with a 1:1 allocation ratio. At the end of the initial visit, each child was randomly allocated to either the intervention or control group. This platform used a permuted block design stratified on the center and gemellarity: singleton or twin (ie, if 1 twin had a WM anomaly, only this child was selected for randomization; if both twins had a diminished WM, both were randomized to the same group to reduce contamination). Children randomized to the intervention group received cognitive training.^26^

### Statistical Analysis

#### Data Imputation

Intention-to-treat analyses were done. To fortify the validity of the results and get the most accurate regression model estimates, a multiple imputation procedure was conducted before all analyses. The distribution of missing data are shown in eTable 6 in Supplement 2. To contextualize the impact of imputation, univariate and multivariate analyses without multiple imputations were also performed but were not reported.

The imputation of multiple data sets was carried out using the chained equations algorithm.^34^ Five imputation data sets were used according to previous literature.^35,36,37^ The imputed variables were those of the main criterion and the secondary criteria, assessed at the 3 evaluation times, as well as the adjustment variables, as described in the next section.

#### Statistical Modeling of the Primary and Secondary Outcomes

To assess the effect of cognitive training on the outcomes at each time point (baseline and after finishing training at 6 months [±2 months] and 16 months [±2 months]), a mixed model for repeated measures was performed for each outcome. We systematically adjusted for confounding by including the following confounders in the mixed regression model: neurodevelopmental profile severity (for 3 subtests of NEPSY, 2nd Edition^38^: auditory attention score, design fluency score, and inhibition score); 1 subtest of the WPPSI-IV (processing speed index); the presence of an impairment in motor performance, assessed by the Movement Assessment Battery for Children, 2nd Edition^39^ (scores ≤5th percentile); and gestational age, birth weight, child’s sex, parents’ socioprofessional status, and parents’ educational levels. These confounding factors were selected by univariate preselection using a threshold of *P* ≤ .10 or by their clinical relevance. Estimated marginal means for each group at each time were reported. The unstructured covariance matrix was used for the within-subject correlation.

An additional sensitivity analysis using the previous mixed model was conducted for participants who completed at least 15 training sessions by recommendations of the cognitive training program to assess the conclusions’ robustness. When appropriate, 95% CIs were presented. The α level of significance was set at 5%; hence, 2-sided *P* < .05 was considered to indicate a statistically significant difference between the 2 groups. According to Cohen criteria, the size difference between the 2 groups was measured by the effect size.^40^

Another sensitivity analysis was conducted for participants who completed at least 20 or 25 cognitive training program sessions. Data were analyzed from February to December 2020 using IBM SPSS Statistics for Windows, version 20.0. The statistical analysis plan is available in Supplement 1.

## Results

### Participants

**Figure 1.**
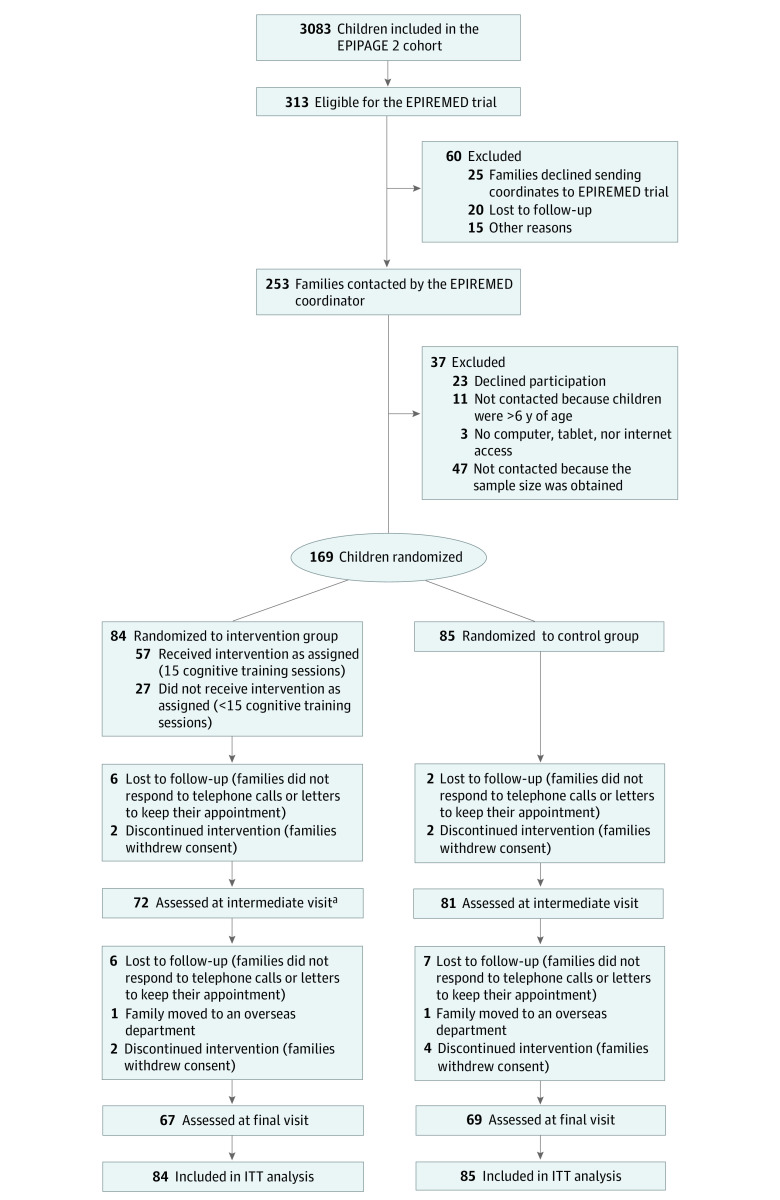
CONSORT Flow Diagram Overseas departments are territorial authorities integrated into the French Republic: Guadeloupe, Martinique, French Guiana, Reunion Island, and Mayotte. ITT indicates intention-to-treat. ^a^A total of 4 families did not keep scheduled appointment but were assessed at the final visit.

Of the 3083 children in the EPIPAGE 2 cohort, 313 were eligible for the EPIREMED trial. The trial’s enrollment consisted of 169 children with a mean (SD) age of 5 years 11 months (2 months), mean (SD) FSIQ score of 88.5 (10.3), and mean (SD) WM score of 80.0 (5.3); 91 children (54%) were female, and 78 (46%) were male. Among these children, 84 were randomized to the intervention group and 85 to the control group. The retention rate at a mean (SD) of 3.0 (1.8) months was 91% and at a mean (SD) of 12.9 (2.6) months was 80% (Figure 1). Demographic and clinical characteristics at birth were similar between participants and nonparticipants (eTable 2 in Supplement 2). The demographic and clinical characteristics at birth and the randomization baseline and the primary end point (VSI score) in both treatment groups appear in Table 1. The neurodevelopmental profile was compared between the 2 groups, with the intervention group having the better profile (Table 2).

**Table 1.** Demographic and Clinical Characteristics of Participants at Birth and Randomization Baseline

Characteristic	Total missing data, No. (%)	Participants (N = 169)^a^	
		Intervention group (n = 84)	Control group (n = 85)
**At birth**			
Maternal age, mean (SD)	0	30 y 3 mo (6 y 2 mo)	30 y (6 y 4 mo)
Parents’ socioprofessional status, No. (%)^b^			
Higher	10 (6)	63/81 (78)	61/78 (78)
Lower		17/81 (21)	16/78 (21)
Without socioprofessional status		1/81 (1)	1/78 (1)
Antenatal corticosteroid therapy, full course minimum	12 (7)	50/78 (64)	52/79 (66)
Antenatal administration of magnesium sulfate during last hospitalization	5 (3)	8/82 (10)	14/82 (17)
Multiple births	0	20/84 (24)	27/85 (32)
Gestational age, mean (SD), wk	0	29.8 (2.7)	29.3 (2.5)
Birth weight, mean (SD), g	0	1357.6 (496.7)	1277.1 (514.0)
Birth weight, percentile of EPOPé curves			
<10th	0	35/84 (42)	39/85 (46)
≥10th		49/84 (58)	46/85 (54)
Child’s sex			
Female	0	42/84 (50)	36/85 (42)
Male		42/84 (50)	49/85 (58)
Mode of delivery			
Vaginal without instruments	3 (2)	17/84 (20)	23/82 (28)
Vaginal with instruments		4 /84 (5)	2/82 (2)
Cesarean		63/84 (75)	57/82 (70)
Apgar at 5 min, mean (SD)	0	8.1 (2.1)	8.4 (1.9)
Bronchopulmonary dysplasia^c^			
None	6 (4)	63/81 (78)	59/82 (72)
Mild		10/81 (12)	10/82 (12)
Moderate		1/81 (1)	3/82 (4)
Severe		7/81 (9)	10/82 (12)
Postnatal corticotherapy	6 (4)	7/82 (9)	11/81 (14)
Intraventricular hemorrhage			
None	2 (1)	66/84 (79)	55/83 (66)
Grade 1 or 2		15/84 (18)	25/83 (30)
Grade 3 or 4		3/84 (4)	3/83 (4)
Necrotizing enterocolitis	3 (2)	4/84 (5)	8/82 (10)
Severe neonatal morbidity	5 (3)	14/83 (17)	18/81 (22)
Late-onset sepsis	2 (1)	13/84 (15)	21/83 (25)
Presence of severe lesions on last TFU scan before discharge home	2 (1)	4/84 (5)	3/83 (4)
**At randomization baseline**			
Child’s age, mean (SD)	0	5 y 11 mo (2 mo)	5 y 10 mo (2 mo)
Parents’ educational level^d^	12 (7)	56/79 (71)	46/78 (59)
Full-scale IQ>70	0	84/84 (100)	85/85 (100)
Working memory index ≤85	0	84/84 (100)	85/85 (100)
Visuospatial index			
Score, mean (SD)	0	95.5 (10.9)	92.2 (11.9)
*Z*-score, mean (SD)		−0.3 (0.7)	−0.5 (0.8)

Abbreviations: EPOPé, Obstetrical, Perinatal and Pediatric Epidemiology Research Team at the Center for Research on Epidemiology and Statistics Sorbonne Paris Cité; TFU, transfontanellar ultrasonography.

^a^ Data are presented as the number of participants with data/total number (percentage) unless otherwise indicated.

^b^ Higher socioprofessional status comprises 3 working categories: managerial, intermediate, and administrator. Lower socioprofessional status comprises 2 categories: domestic or sales employee.

^c^ Mild was defined as 28 or more days of oxygen and spontaneous respiration-room air at 36 weeks; moderate, 28 or more days of oxygen and mechanical ventilation or continuous positive airway pressure or fraction of inspired oxygen greater than 21% at 36 weeks; and severe, 28 or more days of oxygen and mechanical ventilation or continuous positive airway pressure or fraction of inspired oxygen 30% at 36 weeks.

^d^ At least 1 parent with a minimum of 1 year of university education.

**Table 2.** Neurodevelopmental Profile of Randomized Participants

	Intervention group (n = 84)	Control group (n = 85)	*P* value	Effect size^a^
Child age, mean (SD)	5 y 11 mo (2 mo)	5 y 10 mo (2 mo)	.14	0.2
Visuospatial index				
Score, mean (SD)	95.5 (10.9)	92.2 (11.9)	.07	0.2
*Z*-score, mean (SD)	−0.3 (0.7)	−0.5 (0.8)		
Working memory index				
Score, mean (SD)	80.5 (4.7)	79.4 (5.7)	.15	0.2
*Z*-score, mean (SD)	−1.3 (0.3)	−1.4 (0.4)		
Total MABC-2 score, mean (SD)	77.3 (13.3)	71.6 (14.7)	.01	0.3
Intellectual functioning				
Full-scale IQ				
Mean (SD)	90.6 (10.9)	86.4 (9.3)	.008	0.3
*Z*-score, mean (SD)	−0.6 (0.7)	−0.9 (0.6)		
Verbal comprehension index				
Score, mean (SD)	97.3 (13.9)	95.0 (12.9)	.26	0.1
*Z*-score, mean (SD)	−0.2 (0.9)	−0.3 (0.9)		
Fluid reasoning index				
Score, mean (SD)	95.8 (12.9)	92.2 (11.5)	.06	0.2
*Z*-score, mean (SD)	−0.3 (0.9)	−0.5 (0.8)		
Processing speed index				
Score, mean (SD)	93.0 (12.3)	88.2 (11.9)	.01	0.3
*Z*-score, mean (SD)	−0.5 (0.8)	−0.8 (0.8)		
Executive and attention processes				
Inhibition				
Score, mean (SD)	8.6 (3.0)	7.1 (3.1)	.005	0.4
*Z*-score, mean (SD)	−0.5 (1.0)	−1.0 (1.0)		
Statue				
Score, mean (SD)	9.2 (3.2)	9.3 (3.4)	.84	<0.2
*Z*-score, mean (SD)	−0.3 (1.1)	−0.2 (1.1)		
Speeded naming				
Score, mean (SD)	10.3 (3.0)	9.0 (2.7)	.03	0.3
*Z*-score, mean (SD)	0.1 (1.0)	−0.3 (0.9)		
Auditory attention				
Score, mean (SD)	8.8 (3.6)	7.5 (2.9)	.01	0.3
*Z*-score, mean (SD)	−0.4 (1.2)	−0.8 (1.0)		
Design fluency				
Score, mean (SD)	8.4 (2.6)	7.5 (2.6)	.04	0.3
*Z*-score, mean (SD)	−0.5 (0.9)	−0.8 (0.9)		
Educational assistance at school^b^	9/74 (12)	23/75 (31)	.006	0.5
Medical consultation^c^	39/72 (54)	49/76 (64)	.20	0.2

Abbreviation: MABC-2, Movement Assessment Battery for Children, 2nd Edition.

^a^ Effect size measures the size of the difference between the 2 groups. A positive value reflects a positive effect on the score and a negative value, a negative effect on the score in the intervention group. According to Cohen criteria, effect sizes could be categorized as small (0.2), medium (0.5), or large (≥0.8).

^b^ Children who benefitted from any of the following school assistance: special needs teaching assistant, assistance by any other professional, technical aid, personalized educational plans for children with disabilities, and other types of support. Data were missing for 20 participants (12%).

^c^ Children who, in the 12 months before inclusion in the study, were followed up at a specialist center (Early Medical-Social Action Centers and Medical Psychologic Centers of Early Childhood) or benefited from 1 or more medical consultations by the following professionals: psychologist, psychometrician, orthoptist, speech therapist, and/or physiotherapist. Data were missing for 21 participants (12%).

The participants randomized to the intervention group completed a mean (SD) of 18.1 (9.1) training sessions. Among them, 57 children (68%) completed 15 or more sessions, 52 (62%) completed 20 or more sessions, and 43 (51%) completed 25 sessions, with mean (SD) progression index scores of 23.4 (6.4), 23.8 (6.3), and 24.0 (6.3), respectively. The workforce’s power was reduced to 32% at 15 sessions, 38% at 20 sessions, and 49% at 25 sessions (eTable 3 in Supplement 2). Mean (SD) duration of training was 3.2 (1.6) months. Children whose parents had a higher socioprofessional status had better adherence (eTable 4 in Supplement 2).

### Outcomes

#### Primary Outcome

**Figure 2.**
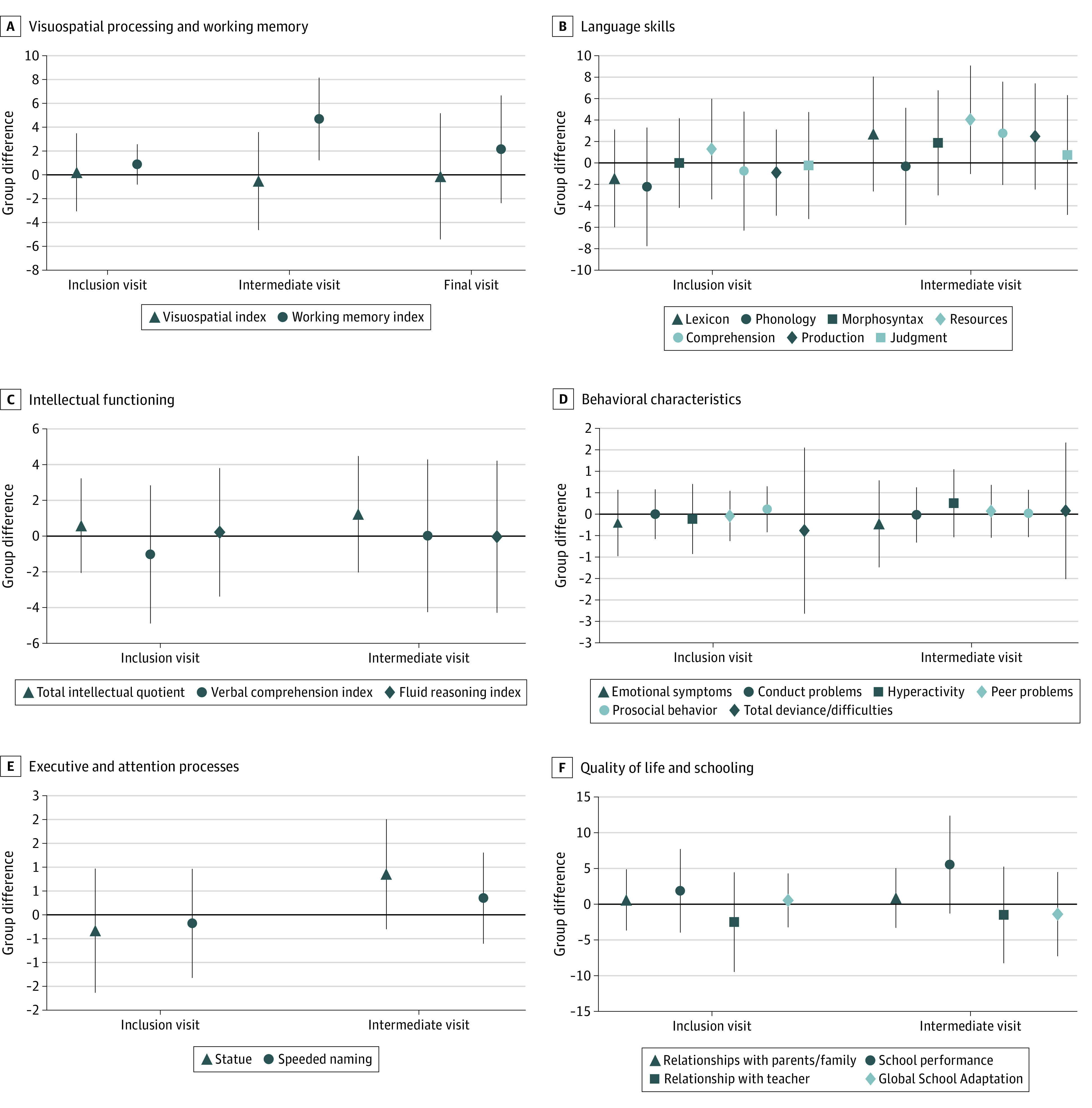
Treatment Group Differences at Inclusion and the Intermediate and Final Visits Results are adjusted for neurodevelopmental profile severity for 3 subtests of NEPSY, 2nd edition (auditory attention score, design fluency score, and inhibition score) and 1 subtest of the Wechsler Preschool and Primary Scale of Intelligence, 4th Edition (processing speed index); the presence of an impairment in motor performance as assessed by the Movement Assessment Battery for Children, 2nd Edition (scores ≤5th percentile); and gestational age, birth weight, child’s sex, parents’ socioprofessional status, and parents’ educational level. Point estimates reflect regression coefficients from mixed-effects models with missing data imputations, and error bars represent 95% CIs. Group difference greater than 0 reflects a higher score in the intervention group, and less than 0 reflects a lower score. Language skills were assessed using the Communiquer, Lire et Écrire Pour Apprendre.

There was no group difference in VSI score at a mean (SD) of 3.0 (1.8) months (difference, −0.6 points; 95% CI, −4.7 to 3.5 points) or 12.9 (2.6) months (difference, 0.1 points; 95% CI, −5.4 to 5.1 points) after finishing training, even after adjustment for both neurodevelopmental profile severity and multiple imputation (Figure 2). The sensitivity analysis adjusted for the same factors confirmed this result (Table 3), as did the analyses with 20 and 25 sessions (eTable 5 in Supplement 2).

**Table 3.** Sensitivity Analysis Adjusted With Missing Data Imputations Restricted to 142 Children Who Completed at Least 15 Training Sessions

	Inclusion visit			Intermediate visit			Final visit		
	Group difference (95% CI)^a^	*P* value^b^	Effect size^c^	Group difference (95% CI)^a^	*P* value^b^	Effect size^c^	Group difference (95% CI)^a^	*P* value^b^	Effect size^c^
Visuospatial index	1.2 (−2.6 to 5.1)	.53	0.6	0.4 (−3.8 to 4.6)	.86	0.2	0.6 (−5.9 to 7.0)	.86	0.2
Working memory index	1.0 (−1.0 to 3.1)	.32	1.0	6.5 (3.1 to 10.0)	<.001	3.9	3.7 (−1.1 to 8.5)	.13	1.5
Intellectual functioning									
Full-scale IQ	1.6 (−1.5 to 4.7)	.31	1.0	3.0 (−0.4 to 6.4)	.09	1.7	NA	NA	NA
Verbal comprehension index	−1.0 (−5.3 to 3.2)	.64	−0.4	0.7 (−3.9 to 5.2)	.78	0.3	NA	NA	NA
Fluid reasoning index	4.0 (0.04 to 8.0)	.05	1.9	2.4 (−1.7 to 6.6)	.25	1.2	NA	NA	NA
Executive and attention processes									
Statue	0.2 (−1.4 to 1.7)	.82	0.2	0.6 (−0.7 to 1.9)	.34	0.9	NA	NA	NA
Speeded naming	−0.3 (−1.8 to 1.2)	.69	−0.4	0.4 (−0.7 to 1.5)	.51	0.7	NA	NA	NA
Language skills^d^									
Lexicon	−0.8 (−6.2 to 4.6)	.77	−0.3	3.1 (−2.6 to 8.8)	.29	1.1	NA	NA	NA
Phonology	−1.7 (−8.0 to 4.6)	.59	−0.5	0.8 (−5.3 to 6.9)	.80	0.2	NA	NA	NA
Morphosyntax	0.5 (−4.3 to 5.3)	.84	0.2	3.3 (−1.6 to 8.3)	.19	1.3	NA	NA	NA
Resources	2.0 (−3.4 to 7.4)	.47	0.7	4.6 (−0.8 to 10.0)	.10	1.6	NA	NA	NA
Comprehension	−0.01 (−6.5 to 6.5)	>.99	0.0	2.8 (−2.3 to 8.0)	.28	1.0	NA	NA	NA
Production	0.2 (−4.5 to 5.0)	.93	0.1	4.0 (−1.0 to 9.0)	.12	1.5	NA	NA	NA
Judgment	−0.2 (−5.9 to 5.6)	.96	−0.1	1.9 (−4.4 to 8.2)	.56	0.6	NA	NA	NA
Behavior									
Emotional symptoms score	−0.2 (−1.1 to 0.7)	.64	−0.4	−0.2 (−1.2 to 0.8)	.73	−0.3	NA	NA	NA
Conduct problems score	−0.03 (−0.7 to 0.6)	.94	−0.1	0.3 (−0.5 to 1.0)	.49	0.7	NA	NA	NA
Hyperactivity score	−0.3 (−1.3 to 0.6)	.48	−0.7	0.4 (−0.6 to 1.3)	.45	0.7	NA	NA	NA
Peer problems score	−0.2 (−0.9 to 0.4)	.50	−0.7	0.1 (−0.6 to 0.8)	.75	0.3	NA	NA	NA
Prosocial behavior score	0.2 (−0.4 to 0.8)	.48	0.7	0.04 (−0.6 to 0.7)	.91	0.1	NA	NA	NA
Total deviance or difficulties score	−0.8 (−3.0 to 1.4)	.47	−0.7	0.6 (−1.3 to 2.5)	.55	0.6	NA	NA	NA
Quality of life									
Relationship with parents and family	−0.8 (−5.8 to 4.1)	.74	−0.3	1.5 (−2.8 to 5.8)	.49	0.6	NA	NA	NA
School performance	2.7 (−4.4 to 9.8)	.46	0.7	5.4 (−1.5 to 12.4)	.13	1.5	NA	NA	NA
Relationship with teacher	−2.6 (−10.7 to 5.6)	.53	−0.6	−1.5 (−8.9 to 5.8)	.68	−0.4	NA	NA	NA
Schooling									
Global school adaptation score	0.9 (−2.9 to 4.7)	.65	0.4	0.1 (−6.1 to 6.3)	.97	0.04	NA	NA	NA
Parents’ anxiety									
Anxious state score	−1.5 (−5.2 to 2.2)	.42	−0.8	−0.7 (−4.0 to 2.7)	.69	−0.4	NA	NA	NA
Anxious trait score	−0.7 (−4.1 to 2.7)	.68	−0.4	−0.02 (−3.4 to 3.4)	>.99	0.01	NA	NA	NA

Abbreviation: NA, not applicable.

^a^ Group differences greater than or less than 0 reflect a higher or lower score in the intervention group, respectively.

^b^ The Statistical Modeling of the Primary and Secondary Outcomes subsection of the Methods section gives the adjusted variables.

^c^ Positive value reflects a positive effect on the score and a negative value, a negative effect on the score in the intervention group.

^d^ Assessed using Communiquer, Lire et Écrire Pour Apprendre.^32^

#### Secondary Outcomes

At the intermediate point (mean [SD], 3.0 [1.8] months after finishing training), there was a positive effect of the cognitive training program on WM only (difference, 4.7 points; 95% CI, 1.2-8.1 points); even after adjustment and multiple imputation, this effect was not maintained long term (Figure 2). The sensitivity analyses indicated a significant effect on WM, with a marginal clinical effect on resource skill language while exploring the short-term mnemonic span (Table 3). This trend persisted when we increased the number of sessions to 20 and 25 (eTable 5 in Supplement 2).

## Discussion

In children born very preterm with WM impairment, a cognitive training program showed no medium-term or long-term effect on visuospatial skills. Activation of the WM was transient with no lasting retention and did not lead to any significant transfer near (other EFs, other cognitive functions) or far (concerning the child’s behavior, quality of life, or learning).

This large study is, to our knowledge, the first multicenter computerized WM cognitive training study involving children aged 5 to 6 years born very preterm with a long-term effect evaluation. Among the few studies using the same cognitive training program in children born prematurely, the population size, conditions of the control group, waiting list or active group,^20^ inclusion of children born at full term,^18,21,41^ and differences in the age at cognitive training administration make comparisons difficult. One study in school-aged children born very preterm (IMPRINT)^21,22^ that was comparable to the current study in size and results showed transitory improvement in verbal WM 2 weeks after administration of the same cognitive training program but without any long-term effects. In contrast to our study, the control group received the same intervention as the cognitive training program group except that the WM load was set at a low level. We used a passive group for our study feasibility, thus not making it possible to control the effects of parental care and charisma. However, a meta-analysis^15^ of the outcomes of cognitive training during preschool age showed that there were no differences for this age group whether using a passive or an active group. Conversely, the same cognitive training program used in this study improved visual inhibition disorders in adolescents who had congenital heart disease with a high prevalence of executive dysfunctions and attention disorders similar to those in children born preterm at the 3-month follow-up but not the long-term follow-ups.^25^ This improvement was attributed to the increased motivation of the participants promoted by the video game–like nature of the program and to their neurodevelopment.^25,42^ This inhibition use develops throughout childhood and adolescence whenever a conflict arises between the 2 modes of reasoning. The child or adolescent learns to resist the impulse of the fast (heuristic) and easy strategy in a cognitive trap situation in favor of the onerous algorithmic logical reasoning.^8,43^ Thus, this triple system—heuristic, algorithmic, and inhibitory—is the basis of cognitive reasoning. The children’s young age is probably the key for not enhancing other areas of EF (inhibition) with a computerized cognitive training program. The EFs are not well defined during preschool and school age, especially inhibition, which dissociates as age increases.^44,45^

Similarly, it should be noted that quality of adherence (progression index) is difficult to achieve. In our study, as in the IMPRINT study,^22^ we found weak evidence that a cognitive training program could clinically boost resource skills language or explore the short-term mnemonic span related to verbal WM in children born very preterm at the intermediate point with children who completed more than 15 sessions.^22^ This effect may be in favor of a close transfer to an untrained function (verbal WM) in relation to the holistic nature of development: language requiring a certain number of good-quality functions to develop, including EFs.^46^ Therefore, use of a cognitive training program could, by training visuospatial WM, improve verbal WM, which is not directly trained, due to the intercorrelation and overlap between EFs at this developmental age.^15^

Long-term outcome assessments were comprehensively extended to cognitively trained WM and nontrained functions, such as visuospatial skills. We consider the VSI as sound according to different criteria: (1) children born very preterm are particularly at risk of developing local or global processing difficulties mediated by the inhibition visual disorders^23^; (2) Calderon and colleagues^25^ showed a transfer of WM training (with the same cognitive training program) to inhibitory visual control; and (3) there are, to our knowledge, no studies proving the long-term effectiveness of this cognitive training program.^47^ Our measurement tests had good psychometric quality (validity and reproducibility), showing good sensitivity to change, good ecological validity, and a low retest effect. The Communiquer, Lire et Ecrire Pour Apprendre is a battery for assessing linguistic skills, and its morphosyntax and resources include pragmatic and semantic aspects linked to EF.^32^ Thus, our study measured the benefits of WM training at different levels to analyze the mechanisms underlying its benefits.^48^ Although it seems generally accepted that cognitive training for an EF can lead to an improvement in the same function, a transfer of improvement to a closely related function is possible by soliciting the same domains of neural networks.^44,49^ Finally, we used a mixed model, hypothesizing that individual differences in the baseline efficiency of fundamental executive processes could modulate neuroplasticity following cognitive training. In other words, EFs change with age in terms of onset and development speed.^7^ The retention rate at a mean (SD) of 3.0 (1.8) months was excellent (91%) and was good (80%) at a mean (SD) of 12.9 (2.6) months, but treatment adherence (at least 15 sessions) was obtained for 68% of patients as proof of study feasibility. Statistical analysis took into account treatment adherence (sensitivity analysis) and confounding factors that could influence adherence, such as parents’ socioprofessional status and educational levels.

For preschool-aged children born very preterm whose entire neurological development is affected (cognitive, behavioral, and social components),^50,51^ programs such as Montessori-type school programs or Tools of the Mind in North America that integrate emotional and social components, including psychomotor components such as yoga or martial arts, would probably be more efficient.^15^ The advantages of cognitive training for EFs in a group setting, such as school, rather than individual computerized cognitive training, as in our study, are peer motivation and interaction, which prompt emulation and are more in line with the heuristic development of preschool-age children. In this young age group, card games, body exercises, and paper-and-pencil activities may be more appropriate to arouse motivation and less costly in attention and would correspond more to the strategy of cognitive reasoning.^44^

### Limitations

This study has limitations. At 15 sessions, the sensitivity of our results improved, but our workforce’s power was reduced. The use of methods for imputing missing data preserved the statistical power used to estimate the sample size, and nonresponders were likely to have outliers. Therefore, the loss of these nonresponders could underestimate variability and thus result in an artificially narrow CI for the cognitive training effect. Even if the randomization was homogeneous on the basic eligibility criteria, this would not reflect a potential flaw in the randomization process or a random allocation of severity. The groups born very preterm presented heterogeneity in the severity of their neurodevelopmental profile,^13,52^ which may have influenced the training’s efficacy. These differences are almost obligatory if randomization is by individual rather than severity cluster on the neurodevelopmental phenotype of children born very preterm. Results of the IMPRINT study showed an FSIQ difference of severity between the 2 preterm groups at baseline.^22^

At baseline, the intervention group had fewer special educational services. During the training, this frequency was not reported, although this might have influenced the outcome. The neuropsychological assessment was carried out by testers not blinded to the child’s assignment to either the intervention group or the control group; the tester could have given parents suggestions to improve the poor results in the control group, thereby reducing a potential effect of the intervention, or in other ways as if it were an expectancy bias that the intervention should work. However, we found no other options to ensure the feasibility of the study, and testers were asked to adhere to the test guidelines as much as possible.

## Conclusion

In this randomized clinical trial, there was no lasting effect of a cognitive training program on VSI scores in children aged 5½ to 6 years born very preterm, suggesting that it is challenging to improve EFs. Other studies also failed to find long-lasting effects, as reported in a previous meta-analyses.^53^ These results underline the importance of a minimum cognitive training adherence time before observing a significant improvement in trained or untrained functions, and maintaining this benefit probably requires repetitive cognitive training.

## Notes

Supplement 1.
Trial Protocol

Supplement 2. **eTable 1.** Collected Measures
eReferences
**eTable 2.** Demographics and Clinical Characteristics of the Included and Nonincluded Children at Birth and at 5 Years Old **eTable 3.** Description of Training Adherence in the Intervention Group **eTable 4.** Training Adherence in the Intervention Group According to Parents’ Socioprofessional Status and Educational Level With Missing Data Imputation **eTable 5.** Sensitivity Analysis Adjusted With and Without Missing Data Imputations Restricted to Children Who Completed at Least 20 or 25 sessions of Training in the Intervention Group **eTable 6.** Missing Data Distribution

Supplement 3.
EPIREMED Study Group

Supplement 4.
Data Sharing Statement
