# Supplementary material for: Cognitive Training for Visuospatial Processing in Children Aged 5½ to 6 Years Born Very Preterm With Working Memory Dysfunction: A Randomized Clinical Trial
Source: JAMA Netw Open. 2023 Sep 7;6(9):e2331988. doi: 10.1001/jamanetworkopen.2023.31988 (PMC10485729; doi:10.1001/jamanetworkopen.2023.31988)
Supplement: Supplement 1. — Trial Protocol [file jamanetwopen-e2331988-s001.pdf]

Long-term effects of visual spatial working memory training program performed at preschool age in very preterm infants with visual spatial working memory deficit
A Randomized Controlled Trial
EPIREMED

Programme Hospitalier de Recherche clinique
Appel à projets national 2015

Coordinator

Dr Catherine Gire	Neonatology, CHU Marseille, hôpital Nord, AP-HM, Marseille

Co-investigators

Pr Arnaud Catherine	Toulouse France Département de Santé Publique, Université de Toulouse III UMR 1027 Inserm
Pr Bednarek Nathalie	Reims France CHRU Reims
Pr Cambonie Gilles	Montpellier France CHRU Montpellier
Pr Claris Olivier	Lyon France Hospices Civils de Lyon
Pr Debillon Thierry	Grenoble France CHRU Grenoble
Dr Foix-Hélias Laurence	Paris France APHP - Trousseau
Dr Garcia Patricia	Marseille France APHM - Conception
Dr Granier Michelle	Paris France Centre Hospitalier Sud Francilien
Pr Guillois Bernard	Caen France CHRU Caen
Pr Hascoet jean Michel	Nancy.France. CHRU. Nancy
Pr Kuhn Pierre	Strasbourg France CHRU Strasbourg
Dr Lecomte Benedicte	Clermont-Ferrand.France. CHRU Clermont Ferrand
Dr Maillotte Anne Marie	Nice.France. CHU NICE
Pr Mitanchez Delphine	Paris France APHP - Trousseau
Pr Marret Stephane	Rouen France CHRU Rouen
Pr Patural Hugues	Saint Etienne France CHU Saint-Etienne
Pr Picaud Jean-Charles	Lyon France Hospices Civils de Lyon
Pr Roze Jean Christophe	Nantes France CHRU Nantes
Pr Saliba Elie	Tours France CHRU tours
Dr Souksi Médioni Isabelle	Montpellier CHU
Dr Tréluyer Jean-Marc	CIC Necker APHP

Collaborateurs

Mrs Calderon Johanna	Paris France Inserm, Equipe EPOPé, Unité 1153 Unité 1153

Methodologists

Pr Pascal Auquier	Public Health, CHU Timone, AP-HM
Dr Pierre-Yves Ancel	Inserm u 1153. Equipe EPOPÉ
Dr Karine Baumstarck	Clinic Research Plateform, AP-HM
Ms Monique Kaminski	Inserm u 1153. Equipe EPOPÉ

**Project management**	Direction of research and clinical innovation (DRCI) – APHM
**Quality assurance**	Good clinical practices and vigilances platform – DRCI APHM
**Sponsor**	Assistance Publique – Hôpitaux de Marseille

Sommaire

I SUMMARY 5

II INTRODUCTION: BACKGROUND AND RATIONALE 11

2.1 Premature birth concerns on public health issues 11

2.2 Executive Functions and, in particular, Working Memory 12

2.3 Executive Functions and Working Memory in premature children 14

2.4 Cognitive Training 16

2.5 Working hypothesis 18

2.6 EPIREMED: a new randomized controlled trial nested in the ongoing French national cohort EPIPAGE 2. 19

III OBJECTIVES 21

3.1 PRIMARY OBJECTIVE 21

3.2 SECONDARY OBJECTIVES 21

IV METHODOLOGY 22

4.1 DESIGN 22

4.2 PARTNERS 22

4.3 STUDY POPULATION 24

4.4 THE TWO STRATEGIES 24

4.5 ENDPOINTS 26

V GENERAL FRAMEWORK 29

5.1 STEERING COMMITTEE 29

5.2 IDENTIFICATION OF ELIGIBLE CHILDREN 29

5.3 BASELINE ASSESSMENT AND INCLUSION 30

5.4 RANDOMIZATION 30

5.5 FOLLOW UP FOR THE TWO GROUPS 31

VI DATA MANAGEMENT AND STATISTICAL ANALYSIS 32

6.1 SAMPLE SIZE 32

6.2 STATISTICAL ASPECTS 32

VII ORIGINALITY AND INNOVATIVE ASPECTS 35

VIII EXPECTED FINDINGS AND INDIVIDUAL/COLLECTIVE POTENTIAL BENEFITS 36

IX CALENDAR 37

X PROJECT FAISABILITY 38

10.1 PARTICIPATION OF A RESEARCH NETWORK 38

10.2 OTHER ELEMENTS OF PROJECTS FEASIBILITY 38
10.3 TEAM EXPERIENCE IN THE FIELD 38
10.4 FINAL REPORT AND PUBLICATION 40
10.5 CASE REPORTS FORM 40
XI REGULATORY AND ETHICAL ASPECTS 41
11.1 APPROVALS 41
11.2 CONFIDENTIALY 41
11.3 OTHER REGULATORY ASPECTS 41
XIII APPENDIX 51
Appendix 1 – Conceptual model for scholl neuropsychological assessment 51
Appendix 2 – Working Memory model (Baddely and Hitch 1995) 52
TABLE 1 – CT in preschool children in the different metanalysis (Kark 2013, Melby Lervag 2013, Kirk 2015, Magan Spencer –Smith 2015) 53

I SUMMARY

**SHORT TITLE**	**EPIREMED**
**LONG TITLE**	Long-term effects of visual spatial working memory training program performed at preschool age in very preterm infants with visual spatial working memory deficit. A Randomized Controlled Trial : EPIREMED
**TYPE OF STUDY**	Prospective, Randomized, Controlled, multicentric trial nested in a population-based epidemiological survey
**DURATION**	36 months
**NUMBER OF CENTRES**	18 centers
**RATIONALE**	Over the last years in France, Very Preterm (VP) birth rates and survival rate have risen. However, the proportion VP survivors with severe deficits have remained stable. Neuropsychological disorders and behavioral deficits are the deficiencies most encountered and have serious consequences on scholarship, familial and social adaptation as well as an impact on future adult lives. Compared to peers born at term, VP children generally perform poorly in Executive Functions (EF) and in particular in Working Memory (WM).<br>WM is defined as a “brain system that provides temporary storage and manipulation of information necessary for (…) complex cognitive tasks”. WM is regulated by a central executive control system and two subordinate subsystems: the visuospatial sketchpad and the phonological loop. This process is considered as a prerequisite for other EF such as reasoning and planning and for predicting intelligence and academic success. In VP children, WM impairment is linked to learning disabilities and is reported to have a strong influence on language and visuospatial processing. Overall academic achievements are thus impaired and an intervention strategy to minimize prematurity’s long-term WM impact needs to be developed.<br>WM can be improved by adaptive training tasks that encourage individuals to work continuously on their personal WM capacity. In recent years, several Cognitive Training (CT) programs focused on improving WM capacity. These programs have succeeded in improving individual performance in some specific WM capacities, but not in everyday functions such as other EF dimensions, language and visuospatial processing. Therefore, the functional benefits of CT have become controversial. Two recent meta-analyses (2015) were particularly focused on the efficiency of computerized visuospatial WM CT programs: Cogmed. The impact on the trained WM is significant and seems to be sustained over time. Improving non-trained functions such as verbal WM, attention, and secondarily learning disorders, is possible, but has yet to be proven on larger numbers. Further randomized studies are required to test CT efficacy in preschool-age VP infants.
**ORIGINALITY AND INNOVATIVE ASPECTS**	The EPIPAGE 2, a current French national population-based prospective cohort of 4,200 VP, born in 2011, offers an interesting and unique setting

	for carrying out a randomized study : EPIREMED trial.<br>EPIREMED is the largest study to assess the efficacy of a visuospatial CT (Cogmed) in preschool-age preterm children. Visuospatial CT is a well-adapted method to reach WM in this age group whose WM is only visuospatial (subcomponent visuospatial sketchpad), taking advantage of the neuroplasticity period. The central executive control system and the phonological loop will develop in children later on. Furthermore about 30% of VP children have low performances on the visuospatial processing. The intention of the study is to assess CT ability to improve visuospatial processing and moreover to assess its impact on the global function and learning abilities of the brain. To date, three small studies have shown that CT (Cogmed) may lead to improvements in WM and in other untrained activities in preschool age VP infants. If conclusive, this study will open the way for developing a preventative strategy for cognitive disorders in VP infants.
**STUDY OBJECTIVES**	
**Main objective**	The main objective is to assess the long-term effects (18 months) of CT (Cogmed) on visuospatial processing in preschool-age VP infants with WM impairment. Visuospatial processing is a broad cognitive process encompassing many subcomponents such as attention, sensory-motor skills, EF and visuospatial WM.
**Secondary objectives**	To assess, after 6 months of the intervention program, the effects of CT on the following parameters :<br>1 Intellectual functioning : global IQ (Wechsler Pre-Primary Scale of Intelligence)<br>2 Cognitive processing : language and visuospatial processing, working memory, fluidity of intelligence, and speed processing by five primary indexes of the Wechsler Pre-Primary Scale of Intelligence<br>3 Executive functions : auditory attention, planification and inhibition<br>4 Language processing and its abilities : ability of cultural and cognitive verbal learning, phonological judgment and semantics, verbal processing speed, verbal WM, motor programming, visual attention, analogizing ability<br>5 Child behavior<br>6 Parental anxiety<br>7 Child quality of life<br>8 School performance
**ENDPOINT MEASURE**	
**Primary end point (linked to main objective)**	The visuospatial index (VSI) : primary index scores of the WPPSI IV (Wechsler Preschool and Primary Scale of Intelligence). This index enables the assessment of visuospatial processing. It consists of two subtests : block design and object assembly. The average score is 100 and the standard deviation (SD) is 15.
**Secondary end points (linked to secondary**	1 Intellectual functioning : Global IQ, WPPSI IV (Wechsler Pre-Primary Scale of Intelligence)

**objectives)**	2 Cognitive processing, i.e. five primary index scores : Verbal Comprehension [VC], Visuospatial [VS], Working Memory [WM], Fluid Reasoning [FR] and Processing Speed [PS]. At age 5 and ½, each child in the EPIPAGE 2 cohort is assessed with the WPPSI IV. The WM index will detect children eligible for inclusion in the EPIREMED trial<br>3 EF, i.e. three subtests from the developmental Neuropsychological assessment (NEPSY 2) : Design fluency, Auditory Attention and Response set, Statue.<br>4 Language processing and its abilities : Battery CLEA (*Communiquer, Lire et Ecrire pour Apprendre*) 2 to 15 years - Evaluation of linguistic development assessment.<br>5 Behavior assessment by Goodman's Strengths and Difficulties Questionnaire (SDQ).<br>6 Parental anxiety: Spielberger State Trait Anxiety Inventory (STAI).<br>7 Quality of life : wellvalidated standardized French questionnaire assessed by parents (*Vécu et Santé Perçue de l'Adolescent parents*), VSPAp<br>8 School performance: Global School Adaptation questionnaire (GSA).
**STUDY POPULATION**	
**Main inclusion criteria**	1 Infants already included in the EPIPAGE 2 cohort, with a GA between 24 and 34 completed weeks, and with parents' agreement to participate in the follow up<br>2 Assessed with the WPPSI IV tool at age 5 ½ years follow-up<br>3 With a global IQ >70 at assessment with the WPPSI IV<br>4 With WM impairment, ie. a WM index <85 with the WPPSI IV<br>5. Parental consent for their child to participate in this study, coverage by medical insurance, and signed informed consent form
**Main exclusion criteria**	1 Severe cerebral palsy (CP) with a GMFCS score >2 and/or BFMF >2. The severity is based on the Gross Motor Function (GMFCS) and Bimanual Fine Motor Function (BFMF) classification system<br>2 Blindness or amblyopia, defined by a visual acuity <3<br>4 Deafness, as defined by a prescribed hearing aid<br>5 Chromosomal disorder or autistic syndrome<br>6 Children already included in the EPILANG study (another project linked to Epipage 2) or other interventional study<br>7 Children who do not speak French<br>8 Triplets
**EXPERIMENTAL DESIGN**	
	Open Randomized Clinical Trial**.** EPIREMED is a randomized controlled study comparing two strategies: CT versus standard care management.
**General framework**	1 Patient selection at the EPIPAGE 2 cohort follow-up using WPPSI IV (total IQ >70 and WM <85)<br>2 Inclusion and complementary neuropsychological assessment of the

	patients, consisting of the EF (NEPSY 2), language and its abilities (CLEA), infant behavior (SDQ), parental anxiety (STAI), quality of life assessment (VSPAp), School performance (GSA)<br>3 A computer-generated randomized list will be drawn up using a permuted block design (stratified on center and gemellarity, singleton/twin)<br>4 Cogmed program for subjects in the experimental arm and customary care management for subjects in the control arm,<br>5 Follow-up for both groups at 6 and 18 months after inclusion
**Experimental group (Cogmed)**	A "Cogmed" physician trained in computerized CT Cogmed, i.e., a neuropsychologist or speech therapist, independent of the initial assessment, will provide the parents with a document explaining WM and the software. Cogmed JM (4-7 years) is an online computer program for WM rehabilitation. This "Cogmed" physician will schedule and design the structure of the sessions. Depending on where parents can attend and have computer-internet access, sessions will be conducted at home or potentially with a "tutor" at a hospital or reeducation center. The child, accompanied by an adult, completes a series of interactive activities automatically adapted to each individual. After each session, the "Cogmed" physician will check the compliance and results online. The program calculates a performance score, representing the difference between the maximum and starting levels and offering analysis of the subject's progress compared to baseline. The parents are provided with 30-minute discussion every week. The program consists of three 15-minute sessions per week for 8 weeks.
**Control group**	The rehabilitation program will not be conducted in this group. The children will be followed up with customary care management. Speech therapy and/or academic support are recommended for those experiencing academic difficulties.
**Study schedule**	**We have planned :**<br>**1 For the experimental group :**<br>1.1 For the children : neuropsychological assessment at inclusion and two post-intervention assessments (8 and 18 months after the inclusion), and 8 weeks of CT,<br>1.2 For the parents : program management, regular phone calls to check up<br>1.3 For the teacher and parents : completing questionnaires at inclusion and 8 months after.<br>**2 For the control group :** The children will all undergo the same supplementary tests and the parents and teacher will receive the same questionnaires
**STATISTICAL ANALYSIS**	The sample size was calculated to obtain a 80% power to detect a difference of 7.5 points on the VS index (estimated standard deviation : 15)

	at 18 months between the 2 groups. This has been considered to be clinically significant. With the threshold for statistical significance set at a p-value of 0.05, assuming that a potential 15% of patients will be lost to follow-up, these calculations showed that 144 patients are needed (72 per group). A total of 150 individuals will be included, 75 for the control group and 75 for the experimental group. Assuming that 30-40% of the EPIPAGE 2 infants will present a WM abnormality, i.e. 1,600 infants, the recruitment will be ensured.
**NUMBER OF PARTICIPANTS**	**150**
**FEASIBILITY OF THE PROJECT**	**Participation of a research network.** EPIREMED operates within the EPIPAGE 2 cohort, 4,200 children, with follow-up rates of 87%. EPIPAGE 2, is already associated with nine complementary French projects, as well as the European EPICE and the French national cohort ELFE**.**<br><br>**Other aspects to insure the feasibility of the project.** The EPIREMED centers are organized for the diagnosis of WM deficits. The entertaining aspect of computerized CT (Cogmed) is adapted to the child's age. Cogmed has previously been used in contexts other than prematurity and tested in randomized studies: three of these, for preschool-age children, showed short term efficacy. The involvement of parents that are aware of their children's development helps assure follow up compliance.
**EXPECTED PATIENT OR PUBLIC HEALTH BENEFITS**	This project's primary goal is to demonstrate the necessity of early visuospatial VM assessment within the vulnerable population of VP children, and to prove the feasibility and efficacy of computerized CT using online software programs. Neurodevelopmental problems are common in VP children. Recent publications have reported many disorders in their specific cognitive functions, one of which is WM. Although neuro-development of VP children remains a public health priority because of their increased birth and survival rates, the interventions to improve this issue remain very few. There are currently no truly effective interventions to deal with academic achievement in preschool-age very preterm infants and their neuropsychological problems; thus, currently no specific management care is recommended for these children. Cogmed is based on interactive software and parental support. The program takes into account the child's environment, a factor of great relevance since it is closely linked to the child's future development. The expected benefits of CT (Cogmed) for those children in this study are enhanced WM, possibly leading to better EF by taking advantage of cerebral plasticity. A better global neuropsychological development improvement (language and visuospatial processing) can be expected with an improvement in learning and decreased behavioral problems. For parents, their guidance is required in Cogmed and can reduce their anxiety by fully embracing their role as primary agents in their child's development. This is consistent with recommendations for family-centered healthcare and can significantly

	improve the quality of life in both the children and their parents. In the long term, these improvements might also reduce those global costs linked to the consequences of extreme prematurity. Finally, if proved effective for this vulnerable population, this treatment can be a possible option or alternative in any other preschool population complaining of early academic difficulties related to WM deficits.

II INTRODUCTION: BACKGROUND AND RATIONALE

2.1 Premature birth concerns on public health issues

2.11 Becoming aware of premature birth issues

The EPIPAGE 2 study shows that the advances made in perinatal care have increased the premature survival rates as well as increased their census in the general population (Torchin H 2015, Ancel PY 2015). Each year there are about 10,000 very preterm births (births before 33 weeks of amenorrhea [SA]) in France. The consequent increase in this population's survival rate warrants an evaluation of their future (Ancel PY 2015, Jarjour IT 2015, Doyle LW 2014, Anderson PJ 2014). Severe sequelae such as cerebral palsy have declined, but specific cognitive difficulties, most often in multiple areas, (motor, language, visual-spatial, attention, executive function, etc.) and minor motor disorders are still very frequent. These sequelae can hinder normal scholastic development and social integration and require an increased need for rehabilitation during preschool and school ages (Marret S 2014).

In 1997 the EPIPAGE 1 cohort study included all very premature births in nine French regions in order to assess their neuro-developmental futures at ages 5 and 8 as compared to term births. In this study, 2357 surviving children born very preterm, 1817 (77%) were assessed at a median age of 5 years. 33% had their MPC (Mental Processing Composite) below 85 (i.e., -1 standard deviation (SD) below normal) and there were 12% scoring below 70 (- 2SD) (the threshold indicating cognitive impairment); as compared to 11% and 3% respectively of children born at term (Larroque B 2008). Half of the premature children received care that was mainly speech and psychological therapy (Marret S 2009).

In 1995 the EPICURE study in the United Kingdom and Ireland observed intellectual difficulties in children born extreme premature (before 26 SA). In this study, 308 surviving children, 241 (78%) were assessed at a median age of six years and four months. There were 46% with severe cognitive deficit (IQ <-2SD) and 30% with moderate cognitive impairment (between -1 and -2 SD) (Marlow N 2005).

Overall, there are cognitive deficits associated with prematurity that increase as the gestational age decreases: 1.5 IQ points per week from 33 SA (Johnson S 2007), regardless of improved perinatal care (Anderson PJ 2014, Bhutta AT 2002, Kerr Wilson CO 2012).

2.12 Learning disorders of very and extremely premature infants

It is shown that very premature babies have less success in school and in their professional life. In the EPIPAGE study, 5% of eight year old children born very prematurely were enrolled in institutional or specialized classes and 95% in regular classes. This is compared with 1% and 99% respectively of those children born at term. Among those children in regular classes, 18% of the very premature births repeated a class at least once compared to 5% of term births (Marret S 2009).

Scholastic failure increases with decreased gestational age. There are studies that segregate out mental impairments and neurosensory disorders and focus exclusively on the learning problems of premature children.

Grunau shows that 65% of 8-9 year old who are extremely prematurely born have learning disabilities compared with 13% of full-term children of the same age (Grunau RE 2002). A meta-

analysis confirms the performance difference in these two birth groups with a difference of 0.5 SD for reading, 0.8 SD for phonology and 0.6 SD in mathematics (Aarnoudse-Moens CS 2009).

In a study of school aged children born extremely premature, there is a performance difference of 0.5 to 0.6 SD in reading, mathematics and phonology compared to children born full-term (Hutchinson EA 2013). In the EPICURE study, 11-year old born extremely premature performed at -1 SD in reading and -1.7 SD in math compared to term infants. In the same study, 52% of the premature children were dyslexic vs 11 % of those born full term and respectively, 70% vs 14% had dyscalculia (Johnson S 2009).

Litt reports that school children aged 8-9 year old with less than 750 grams birth weight had seven times more dyslexia, six times more dyscalculia and 13 times more dyscalculia combined with dyslexia than full-term children (Litt J 2005).

These problems persist into adolescence and adulthood resulting in poor integration in high school and university (Saigal S 2006, Mathiasen R 2009, Rai E 2011) as well as increased unemployment, under qualified employment, anxiety disorders and a loss of self-esteem (Boyle MH 2013). Zwicker studied prematurity's impact on the child's and family quality of life (QOL) during the child's early, adolescent and young adult years. The impact appears more marked in preschoolers than in the adolescent and young adult (Zwicker JG 2008).

2.2 Executive Functions and, in particular, Working Memory

2.21 Definitions of Executive Functions (Appendix 1)

Executive Functions (EF) define the cognitive operations that allow an individual to adjust his behavior and activity in response to environmental requirements and fluctuations.

The EF comes into play when an individual is confronted with a "non-routine" situation that requires problem solving. The principal mental processes characterizing EF are:

- organizing and planning data based on what needs to be achieved, choosing relevant information,
- implementing processing operations, inventing new situations and modifying them if they deviate from the purpose (Anderson PJ 2012, Parkinson J 2015),
- suppressing extraneous information, resisting distractions,
- organizing task-relevant information in the memory to use them later (Working Memory : WM).

It is understood that each of these mental processes or "executive mental functions" can be evaluated by "specific" tests but they are often interlinked and dependent on the mental attention processes (auditory and/or visual). For this reason, definitions vary significantly from publication to publication (Epsy KA 2004, Parkinson J 2015).

Schematically we can isolate four main executive mental processes :

1. ***Planning*** : mental diagram of a single action, anticipating the goal to be reached - "how to achieve that goal"
2. ***Flexibility*** : adaptation of an action plan for environmental contingencies, ability to modify strategies in case of error, maintain attention - "the art of adapting to change"

3. ***Inhibition*** : capacity to ignore distractions and resist giving one reply rather than another
4. ***Working Memory (WM)*** : ability to store verbal or visuo-spatial information in one's mental space and to manipulate them, implementing strategies, processing action sequences, reasoning - "*art de faire*".

All in all, the EF are the "superior" functions that play an important role in cognitive development and social adjustment (Diamond A 2013) (Appendix 1).

2.22 Working Memory: the organizational components (Appendix 2)

WM is a key determinant of several higher–order cognitive functions such as reasoning, fluid intelligence, problem solving and language comprehension (Borella E 2010).

According to Baddeley, the working memory (WM) is based on the coordinated functions of a set of autonomous sub-components (Baddeley A 1996, Shuchan B 2008, Cowan N 2014) :

1. ***The phonological loop or verbal WM***. The phonological loop is responsible for storing visually or aurally presented verbal information. It is composed of two sub-components: a phonological reserve and a recapitulating articulatory loop. For example, when auditory information appears, a phonological analysis is stored as phonological codes. Sentence comprehension, particularly sentences that are long and complex, is based on this mechanism. This subcomponent allows to transfer verbal information, which was obtained visually, to the phonological storage system. The phonological loop is only fully operational when a child is about 8 year old, although the first elements appear at age 4.
2. ***The visuo-spatial sketchpad or visuo-spatial WM***. Visuo-spatial information is encoded by the visuo-spatial sketchpad and is distinguished within its interior with a system for descriptive visual information (shapes, colors...) and a system for spatial information (location). Studies have shown that the motor WM is involved in morphogenetic movements (reproduction of a given body form) and topokenetics (movements made according to a goal and taking into account the environmental information). These are in close collaboration with the visuo-spatial Working Memory. The visuo-spatial storage system maintains information and its variations, interacting with a spatial attention system, thereby facilitating the retention of location information. This is used to resolve visual and spatial type tasks such as space orientation when using a map. The visuo-spatial sketchpad is also responsible for the ability to transform and rotate mental images. As a result, we can mentally describe and explore an object or place.
3. ***The Central Administrator***. It has the function of management and control, it also supervises and coordinates the phonological loop and the visuo-spatial sketchpad. It inhibits irrelevant information and activates information stored in long-term memory such as reasoning, decision-making and action planning. It enables the WM to change continuously its responses based on recent internal information (from the long-term memory) or external information (from sensory input). This updating process supervises the passage of information between subsystems and long-term memory.
4. ***The episodic buffer***. This is a temporary storage system for multi-modal information. As a "buffer" it functions as a temporary interface between the phonological loop and the visuo-spatial sketchpad as well as long-term memory. It is "episodic" because it stores "episodes" in which information is integrated in space and time. This buffer is different from episodic memory because it functions as a temporary storage system but can be preserved in

amnesic patients with major impairment of long-term memory. This storage system is also controlled by the central administrator.

2.23 Working Memory development in children

The performance of WM increases with age (Logie R 1996, Pickering MJ 2001). Initially, young children do not have the ability to encode information in phonological form, and the visual-spatial encoding of objects assumes that role (Suchan B 2008, Kail R 1990, Cowan N 2014). There seems to be predominance for spatial encoding up to the age of 8-10 (De Ribeaupierre A, 2000).

Depending on the WM sought, we observe variations of this function. We also note that boys have a visuo-spatial span mnemonic slightly higher than girls. Nonetheless, the rhythm of this span is not correlated with the intellectual development of the child and there are obviously large inter-individual variations.

Some memory functions of the intervening central administrator such as inhibition of irrelevant information, treatment strategies, execution speed, gradually develop during childhood (Pickering SJ, 2001).

The evolution of attentional capacity can therefore affect the child's ability to grasp information and to inhibit irrelevant information (Cowan N 2014). In this sense, the challenge of this developmental phase seems to be particularly focused on the linking between memory processes, executive functions and attention (Rose SA 2011).

2.24 Executive functions, Working Memory and learning difficulties

EF, WM and attention have related developmental and functional operations and are involved in the development of more complex cognitive processes such as language or visuo-spatial performance (Swanson HL 2011). Their alterations can therefore change intelligence as measured by IQ (J Parkinson 2015, Alloway TP 2010). Many studies have confirmed a correlation between EF and WM abnormalities and learning disabilities such as dyslexia or dyscalculia (Gathercole SE 2000, 2004, 2006, Swanson HL 2004, Espy KA 2004, 2006, Alloway TP 2010, Clark CA 2010). Robin and Parkinson confirm this correlation in a meta-analysis of 67 cross-sectional studies published over the last 25 years on school children of varying ages (Robin J and Parkinson J 2015).

Most studies have been published after 2010 and demonstrate an evolving interest in topic with 35% of these studies focusing only on WM. The link between abnormal EF and learning disabilities remains the same in each age group (3-6 years, 6-11 years and 12-18 years) regardless of the analyzed executive function (planning or inhibition or mental flexibility or WM) and the concerned learning disabilities (dyslexia or dyscalculia).

2.3 Executive Functions and Working Memory in premature children

2.31 Executive Functions and prematurity

EF in very preterm children has generated considerable interest, with numerous studies reporting this cognitive domain to be an area of concern for this population. There are two meta-analyses of EF in premature children.

From their studies published between 1990 and 2008, Mulder and al in 2009 analyze each executive process in a total of 830 very prematurely born school children vs 740 born full-term (Mulder H

2009). The authors show a difference of 0.3 SD for inhibition, 0.5 SD for verbal WM and 0.4 SD for the planning compared to the full-term control group. These differences are even greater for those with lesser gestational ages (within 26 SA: a difference of 0.5 SD for the inhibition and 0.7 SD for verbal WM). The gap is interesting, especially for verbal WM, and it increases with the age of children suggesting a worsening over time. Mental flexibility remains unchanged as compared to the control group.

The Aarnoudseen-Moens's review concerns children born between 1998 and 2008 comparing very extremely premature births to those full-term births (Aarnoudseen-Moens CSH 2009). Only two executive components are covered: WM and mental flexibility. This metaanalysis shows a decrease of 0.6 SD for verbal WM and 0.4 SD for the visuo-spatial WM. Differently from Mulder, this difference stabilizes with age, and mental flexibility is impacted 0.5 SD as compared with the control group.

2.32 Working Memory and prematurity

Almost all studies, regardless of the child's age, show a lower verbal and visuo-spatial WM performance in the very premature child compared to full-term children (Anderson PJ 2004 Aarnoudseen Moens CSH-2009, Rose Baron SA 2011).

In very premature born preschool children, visuo-spatial WM is not as good as those born at term (Epsy KA 2002, Borradori Tolsa C 2014, Baron IS 2010). This is confirmed for verbal WM skills for school age children and adolescents (Taylor HG 2000, 2004). In 2013 Omizollo studied the correlation between WM (verbal and visuo-spatial) and the learning of a 7 year old very prematurely born cohort. The very premature group had 2.1 to 3.5 times more deficit in WM than the control group (Omizollo C 2013).

There is a significant risk for dysexecutive disorder in preschool and school age children with very premature births. Despite this, there are very few long-term studies of the trajectory of these dysexecutive disorders and it is not yet clear whether these disorders are responsible for developmental delays (Anderson PJ 2015). In fact, studies of adolescents or adults are contradictory. Dysexecutive disorders reported in prematurely born adolescents and adults suggest these symptoms persist beyond school age (Taylor HG 2004, Novartis C 2007). However, the absence of a difference or a marginal difference of WM performance is found in other studies of adolescents (Rushe TM 2001, Saavalainen P 2007, Curtis WJ 2002).

2.33 Prematurity: diffuse or selective cognitive impairment ?

The extremely preterm birth population is vulnerable to a lower overall IQ than the general population and therefore requires more discriminative neuropsychological testing to identify affected functions (Anderson PJ 2014).

Hutchinson and Anderson speculate on the damage to a primary, original cognitive function (Hutchinson EA 2013 and Anderson PJ 2013, 2014). For example a WM deficit, and/or attention and/or processing speed, which impact other mental processes, could be the cause of delayed language or dysexecutive disorders (Mulder H 2010, 2011).

This is why early determination of specific cognitive profiles, beginning at a pre-school age, could allow more focused monitoring and/or development of interventional strategies (Taylor HG 2006).

Mulder and al were interested in the origins of learning and attention disorders as well as the EF disorders in the very premature vs term-birth children (Mulder H 2010, 2011). These authors report that the processing speed and WM are independent predictors of academic difficulties amongst the very premature-birth preschool children. According to these authors, the processing speed deficit (which is dependent on gestational age) is correlated to EF : WM (verbal and visuo-spatial), inhibition, cognitive flexibility (Mulder H 2010, 2011). Similarly there is a correlation between the measurement of the processing speed and/or WM and the symptoms of impulsivity/hyperactivity, attention disorders.

2.4 Cognitive Training

2.41 Definition of Cognitive Training

The identification of weak processes in some pathologies, as well as improvement in knowledge of brain function development and the possibility of neuronal plasticity, have led teams of researchers to develop intervention programs focusing on cognitive processes described as Cognitive Training (CT). In short, the CT is defined as the rehabilitation of one or several altered cognitive functions. CT is based on the concept that repeated practice within a specific domain will result in gain in both cognitive and behavioral efficiency of the targeted domain as well as subsequently transferring improvements to untrained domains.

Two types of techniques are used:

- Restorative (training of the defected function through repeated practice),
- Development of alternative skills (compensatory strategy).

The CT may be based on different types of materials: paper/pencil, card games, pieces of wood, behavioral therapies, and software. Specific computer techniques (software) have been developed; they continuously and automatically adjust to task levels and difficulties as well as to the subjects' capabilities in order to optimize the effects of training. This made the scientific community react with a sense of caution against this highly lucrative industry (Parkinson J 2015). Similarly the link between EF deficits and learning disabilities has revived the interest of CT techniques in schools so as to improve standards (Parkinson J 2015). For this reason, many researchers have raised the question of rehabilitation of EF in recent years and asked if CT of EF could improve learning in the general population ; in children with neurocognitive disorders ; and more specifically, in those with premature births.

2.42 Cognitive training in the general population: intervention in school

In 2015 Robin and Parkinson, by means of a meta-analysis of school children having no mental deficiencies, report only five randomized studies with control-groups analyzing interventional components (Robin J 2015, Parkinson J 2015). If the executive functioning is changed by intervening in schools, does that then lead to children's improved achievement? The authors note that there are few studies with any controls for such characteristics as parental education, socio-economic status or IQ, although these characteristics have been found to be associated with EF development. These show that the EF could be improved by CT but with little performance impact on reading and/or mathematics. There is only one study (Rabiner DL 2010), performed on school-aged children and using an attention re-education computer program, which improved attention spans and class

behavior but not directly improved achievement. Other criteria such as repeating a class or the student future are not analyzed.

Bierman and Torres describe the range of what are designed to influence EF in school age children and explore whether or not there is evidence that EF improve as a result (Bierman KL 2015, Torres M 2015). They conclude there is a mixed evidence for the malleability of EF as a result of interventions.

2.43 Cognitive Training in population with cognitive disorders (Table 1)

A meta-analysis (Kacrh D 2013) uses the CT studies of EF in children and adolescents in cases of cognitive impairment. The effects are generally low on EF and attention ([0.18 SD, 95% CI 0.11 to 0.47] and [0.17 SD, 95% CI 0.12-0.42]), with an unexceptional WM (0.65 SD, 95% CI -0.12 to 1.42). The most consistent benefit concerns the improvement of behavioral disorders (0.58 SD, 95% CI 0.31 to 0.85), but this result comes from studies with no control groups. Moreover, only two studies (57 patients) involved preschoolers when brain plasticity is the greatest and where there is a significant improvement in intelligence (IQ) and behavior (Galbiati S 2009, Braun J). Several CT meta-analyses of EF in children with attention deficit disorder and hyperactivity (ADHD) show an improvement in trained EF but the effects on ADHD remain moderate and sustained with slow improvement in time. (Rapport MD 2013, Cortese S 2015).

A CT meta-analysis of WM led by Melby-Lervag involving children and adults with cognitive disorders shows a beneficial CT effect on the visuo-spatial long term WM, without influencing other EF, verbal or non-verbal intelligence or learning (Melby-Lervag M 2013). Only three studies involve children less than 7 years (St ClairThompson HL 2010, Thorell L 2008, Bergman-Nutley SB 2011). One other meta-analysis confirms this author's results in children. (Kirk HE 2015).

The main limitation of these meta-analyses is the heterogeneity of the treated disorders: ADHD, TC sequelae, dysexecutive disorders, learning disorders, typically developping children,.etc., appearing in the various age groups as well as different intervention types, their heterogeneous duration, and the different impact indicators that were measured.

However these meta-analyses show in the population of preschool children (despite the limited number (less than 200)), a significant positive CT effect on the WM or other trained EF along with a possible transfer to other functions such as attention, behavior..., intelligence (Soderqvist S 2012, St Clair Thompson HL 2010, Bergman- Nutley SB 2011, Thorell L 2008).

All in all, the EF rehabilitation, and specifically the WM's impact on the various intelligence and learning processes, remains a current scientific debate.

While there are many rehabilitation systems developed, there is no "gold standard" regarding CT benefit in kind, duration and frequency. There exist some methodologically weak studies showing an improved EF with re-education but with little effectiveness on intelligence and learning performance, except in the preschool child.

A large scale, well conducted study on the impact of EF rehabilitation interventions in preschool age children is therefore indicated.

2.44 Cogmed JM : Cognitive Training of the visuo-spatial Working Memory

Cogmed JM is a technique for re-educating the visual spatial WM and is used in many randomized studies of CT, WM and FE (Shinaver CS 2014). It consists of computer software with a specific set of visuo-spatial VM tasks and adjustable levels of difficulty by using a precise algorithm.

Each day, the user performs a predetermined number of events. These results are retained during the training and can provide the basis for further analysis. (Torkel Klingberg, developed by Helena Westerberg and other researchers, Department of Neuropediatrics Astrid Lindgren Karolinska University Hospital and Jonas Beckeman and David Skoglund, video game developers Klingberg).

Two recent meta-analyses (2015) are particularly interested in the efficacy of Cogmed (Shinaver CS 2014, Spencer-Smith M 2015). The impact on the trained WM visual spatial is significant and seems to be sustained over time. Improving non-trained functions such as verbal WM, attention, and secondarily learning disorders, is possible, but has yet to be proven on larger numbers. Above all the WM CT studies of preschool age children were realized with Cogmed JM (Bergman Nutley SB 2011, Thorell LB 2009, and Sodervisqt S 2012).

2.45 Cognitive Training and prematurity

Three studies on premature infants, examined the effects of CT by Cogmed on visuo-spatial WM (Grunewald KH 2013, 2015, Lohaughen GC 2011). The first was a sample of 16 preterm adolescents vs. 19 teenagers with term births. Another was performed on 20 very premature preschool children aged 5-6 years. The third study included 20 very premature preschool children vs 17 in the control group (term birth).

There appears to be a beneficial effect on the trained WM visuo-spatial and a possible transfer to other processes (verbal working memory, attention, etc.). However, these three studies contain very small samples from very different age groups. In two studies the WM is in the process of maturation and in the third, it is practically consolidated. Although, monitoring was limited to seven months and learning disorders were not assessed, they represent preliminary studies with encouraging results.

2.5 Working hypothesis

In recent years the severe deficiencies of prematurity have been stabilized. The majority of sequelae appears as moderate neuropsychological disorders but with serious school, family and social repercussions. These disorders, which persist into adulthood, result in lower social integration. Very premature-birth children present with frequent WM deficits.

WM in these school age children might be correlated with subsequent learning disorders and the origin of complex deficits such as language delays or visuo-spatial performance disorders (Mulder H 2009). This WM vulnerability in the premature needs to be confirmed and might be an avenue for preventive interventions.
WM is a set of processes that maintains active information necessary for performing common cognitive activities, notably the ability to retain information sufficiently long enough so as to manipulate data in the future. WM is controlled by a central administrator module located in the prefrontal cortex that oversees two peripheral modules: the phonological loop and the visual sketchpad. This is linked to other EF such as reasoning, planning, intelligence and academic learning

and participates in multidimensional skills such as language and visuospatial processes (Alloway TP 2006).

A current research question is about the link between EF rehabilitation, including WM, and its impact on intelligence and learning. Cognitive Training (CT) is an interesting approach to the management of WM disorders. While many types of CT interventions improve WM, the benefits on EF, language, visuo-spatial performance and intelligence remain controversial (Parkinson J 2015, Kirk HE 2015, Melby-Lervag M 2013). To date, three small studies have shown that CT (Cogmed) may lead to improvements in WM and in other untrained activities in preschool age VP infants.

Many re-education techniques (books, games, software, etc.) have emerged in recent years without any "gold standard". Cogmed JM software seems efficient among preschool children in recent meta-analyses (Shinaver CS 2015, Melby-Lervag M 2013, Kirk HE 2015) and is also effective in three preliminary studies of preterm infants.
The strategy is to take advantage of brain neuroplasticity by measuring the consequences of visual spatial WM restoration on the overall brain function and learning in very premature preschool children (Grunewald KH 2013, 2015). The hypothesis is that doing CT with preschool children born very preterm (just as their WM is emerging and uniquely non-verbal: visual-spatial sketchpad), may decrease the subsequent dysexecutive disorders, improve intellectual performance (both visuo-spatial and language processing) as well as school integration.

2.6 EPIREMED: a new randomized controlled trial nested in the ongoing French national cohort EPIPAGE 2.

The EPIPAGE 2 is an ongoing national prospective cohort of 4,200 very preterm children born in France in 2011 and offers an interesting and unique setting for implementing a randomized trial: EPIREMED study.

EPIPAGE 2 study aims to examine short- and long-term outcomes of very preterm children and their determining factors. This project seeks to provide new data on the prognosis and etiology of very preterm birth and to assess related medical practices. Accordingly, it should lead to the development of new strategies of management and prevention in high-risk babies. Eligible participants for this prospective population-based study included all live or stillborn infants and all pregnancies terminated between 22 and 31 weeks of gestation occurring in all the maternity units in 25 French regions. In addition, a sample of moderate preterm births, i.e. births and late terminations at 32-34 weeks, was included from the same regions. In all, 7,804 babies (stillbirths and live births) and terminations of pregnancy out of 8,400 eligible births in France in 2011 that were either very preterm (22-31 weeks) or moderately preterm (32-34 weeks) were included. Data on pregnancy, delivery, and neonatal events were extracted from the obstetric and neonatal records. Of the 4,467 children discharged alive from the hospital and eligible for follow-up, 155 (4%) of the families refused further follow-up and 22 died before one-year of age. Finally, 4,290 were included in the follow-up. Nine additional projects are collecting specific data, in addition to the core cohort data, to investigate specific hypotheses among subsamples of the cohort in order to investigate 1) diagnosis of histologic chorioamnionitis, 2) early biomarkers of the child’s health, 3) attitudes of care for extremely preterm infants, 4) painful procedures in neonatal intensive care units, 5) neonatal MRI cerebral abnormalities and their relation to executive functions, 6) associations between early gut

colonization and early and late disease onset, 7) impact of neonatal nutrition on the child's development, 8) mother-infant attachment 9) a randomised, controlled trial to evaluate if a parent-implemented intervention before the corrected age of 3 years in very preterm children with a language delay can improve language.

The follow-up has collected information at corrected ages of 1 (2012) and 2 (2013) years of age and the next steps will be at 5 ½ (2016), 8 (2019), and 12 (2023) years of age.

EPIREMED, the tenth additional project, will be randomised, controlled trial nested in the EPIPAGE 2 cohort. This trial will assess the effectiveness of CT programs on preterm infants of preschool age (5 ½ year old) that have a visuospatial WM deficit. Visuospatial CT is a well-adapted method to reach WM in this age group whose WM is only visuospatial (subcomponent visuospatial sketchpad), thus taking advantage of the neuroplasticity period.

III OBJECTIVES

3.1 PRIMARY OBJECTIVE

The primary objective is to assess the long-term effects (18-months post inclusion) of Cognitive Training (Cogmed JM) on the visual-spatial processing in pre-term birth preschool-aged children with Working Memory impairment.

Visual-spatial processing is a broad cognitive process encompassing many subcomponents such as attention, sensory-motor, executive function and visuo-spatial working memory. The visuo-spatial index is the primary endpoint.

3.2 SECONDARY OBJECTIVES

The secondary objectives are to assess at the six month post-intervention (8 months post inclusion) the effects of the cognitive training on the following parameters:

- Children' parameters :
  - Global intellectual functioning,
  - Different cognitive processes : language, visual-spatial processing, speed processing, working memory, and fluidity of intelligence,
  - Other composites of Executive Functions : auditory attention, flexibility, and inhibition,
  - Language processing abilities : verbal learning abilities (cultural and cognitive), phonological judgment and semantics, verbal processing speed, verbal WM, motor programming, visual attention, and ability to analogize,
  - Behavior and quality of life (QOL),
  - School performance.

- Parents' parameters :
  - Anxiety level.

IV METHODOLOGY

4.1 DESIGN

This trial is a multicenter, randomized, controlled, open-label, two-parallel group study. The recruitment will be prospective.

The two groups are :

- A control group : standard care management
- An experimental group : standard care management in association with a 2-month Cognitive Training program called Cogmed JM

Study design rational:
A Randomized Controlled Trial is the experimental design providing the highest level of evidence with regard to the existing literature in this field. The open design is the only possible option due to the nature of the experimentation. We assume that the primary endpoint will be collected in a standardized procedure ensuring an objective measure. Both the healthcare worker in charge of the evaluation of the primary endpoint and the statistical analysis will be blinded.

4.2 PARTNERS

The multicenter randomized controlled trial, EPIREMED study will use the design of the existing EPIPAGE 2 cohort which is described in section 4.21.

4.21 The EPIPAGE 2 cohort

The study will involve children included in EPIPAGE 2 from the regions participating in EPIREMED. Recruitment will be at the end of the 5 ½ year EPIPAGE 2 assessment.

The EPIPAGE 2 protocol and the main perinatal results are described in two recent publications 2015 (Ancel PY 2014, 2015).

EPIPAGE 2 (an epidemiological study of low gestational ages) is a national prospective cohort study of very premature babies recruited in France in 2011 with the objectives of:

- An improved knowledge of very premature children's futures,
- An assessment and needs forecast for medical and educational care,
- An assessment of the organization of care and medical practices and their impact for the health and development of premature infants,
- An improved knowledge concerning the causes and consequences of prematurity.

The cohort was set up in 25 regions of mainland France and overseas departments/territories. The cohort follows over 4,290 premature children up to age 12 years. Between April and December 2011 these very premature infants (between 22 and 34 weeks of amenorrhea) were included in the study. Information about pregnancy, childbirth and the immediate care of the child were collected in the maternity ward. At the end of their neonatal hospitalization, a complete assessment of their management and complications was compiled. Upon leaving the neonatal unit, all living children with parental consent were included in the follow up.

These children were reassessed at year one and two with self-administered questionnaires completed by both the parents and the attending physician. There was a follow-up rate of 90% and 87% respectively. Continuing assessments are scheduled for 2016, 2019 and 2023 when the children are 5½, 8 and 12 year old. The EPIREMED study will become involved in the 2016 framework corresponding to the 5½ years EPIPAGE evaluation of the children.

According to the needs of the study, there will be a national steering committee comprised of epidemiologists, pediatricians (Dr C Gire as a hospital-based practitioner), obstetricians, and other specialties. This committee will assume responsibility for the scientific and organizational aspects of the study. Each region will have a coordinating team comprised of pediatricians, obstetricians, midwives, and epidemiologists to oversee the study. Dr C Gire will be the PACA-region scientific coordinator. At two years, the PACA questionnaire follow-up rate is 87% for parents and 84% for physicians.

4.22 Investigators and centers

This is a multicenter, interdisciplinary study with recruitment occurring in 18 units in public academic teaching hospitals.

The **investigator coordinator** of the study is Dr Catherine GIRE, CHU Marseille, hospital Nord, AP-HM.

The **co-investigators** are (alphabetic order):

Pr Arnaud Catherine	Toulouse France Département de Santé Publique, Université de Toulouse III UMR 1027 Inserm
Pr Bedbnarek Nathalie	Reims France CHRU Reims
Pr Cambonie Gilles	Montpellier France CHRU Montpellier
Pr Claris Olivier	Lyon France Hospices Civils de Lyon
Pr Debillon Thierry	Grenoble France CHRU Grenoble
Dr Foix-Hélias Laurence	Paris France APHP - Trousseau
Dr Garcia Patricia	Marseille France APHM - Conception
Dr Granier Michelle	Paris France Centre Hospitalier Sud Francilien
Pr Guillois Bernard	Caen France CHRU Caen
Pr Hascoet jean Michel	Nancy.France. CHRU. Nancy
Pr Kuhn Pierre	Strasbourg France CHRU Strasbourg
Dr Lecomte Benedicte	Clermont-Ferrand.France. CHRU Clermont Ferrand
Dr Maillotte Anne Marie	Nice.France. CHU NICE
Pr Mitanchez Delphine	Paris France APHP - Trousseau
Pr Marret Stephane	Rouen France CHRU Rouen
Pr Patural Hugues	Saint Etienne France CHU Saint-Etienne
Pr Picaud Jean-Charles	Lyon France Hospices Civils de Lyon
Pr Roze Jean Christophe	Nantes France CHRU Nantes
Pr Saliba Elie	Tours France CHRU tours
Dr Souksi Médioni Isabelle	Montpellier CHU
Dr Tréluyer Jean-Marc	CIC Necker APHP

Methodological support will be provided by:

- The Clinical Research Platform of AP-HM (responsible : Pr Pascal Auquier ; medical referent : Dr Karine Baumstarck),
- INSERM unit 1153 EPOPÉ team (Dr Pierre Yves Ancel, Monique Kaminski)

4.23 Sponsor

The sponsor, in charge of all regulatory aspects, is represented by the APHM (*Assistance Publique Hôpitaux de Marseille).*

4.3 STUDY POPULATION

Eligible subjects must meet all of the following criteria.

4.31 Inclusion criteria

- Already included in the EPIPAGE 2, born between 24 and 34 weeks' GA (gestational amenorrhea),
- Children aged 5 to 6 years,
- Children exhibiting a total intellectual quotient >70 from the WPPSI IV (during the 5-year assessment in EPIPAGE 2),
- Children having a visuo-spatial Working Memory impairment defined by a working memory index <85 from the WPPSI IV,
- Children with parents (or legal guardians) authorizing participation in the study and a signed informed consent form,
- Children affiliated with medical insurance.

4.32 Non-inclusion criteria

- Children with severe cerebral palsy, based on the Gross Motor Function (GMFCS score >2) and Bimanual Fine Motor Function (BFMF >2) classification system (Elvrum AG 2015, Marois P 2015),
- Children with blindness or amblyopia, defined by a visual acuity <3 (during the 5-year assessment in EPIPAGE 2),
- Children with deafness, as defined by a prescribed hearing aid,
- Children with chromosomal disorder or autistic syndrome,
- Children included in the EPILANG study protocol (an ancillary project to EPIPAGE),
- Children who do not speak French
- Children with parents having no internet connection,
- Triplets.

4.33 Exclusion (premature study end) criteria

- Children and/or parents wishing to interrupt his/her participation during the study.

4.4 THE TWO STRATEGIES

4.41 Experimental group: the Cognitive Training

* Description of the intervention:

A Cogmed trained neuropsychologist or speech therapist, unrelated to the initial assessment, will acquaint the parents with the program which includes a software presentation, the setting of expectations, the CT objectives and a determination of the reward system along with a document to explain WM and software. He/She calendars and establishes the sessions' structure. The purpose of this initial interview is to build support for the patient and the parents.

Cogmed JM (4-7 years) is a computerized, online WM rehabilitation program. This will be usually at home, or at the hospital or in a rehabilitation center with a "tutor" according to the parents' ability and their access to an internet connected computer. The child, who is accompanied by his /her parents, or a "guardian": is given a series of interactive, automatically and individually adapted exercises. In Cogmed JM, sessions last 15-20 minutes, with three exercises out of seven for each session, with a fairground graphic interface.

The Cogmed JM practitioner consults the on-line compliance and exercise results at each session. The program calculates the performance index: the difference between the maximum level and the starting level which is used to assess progress against a standard norm. The parents benefit from a weekly 30-minute interview in order to support and strengthen the patient's motivation. The interview focuses on his/her evolving performance and rewards progress. At approximately the 15th session they are made aware of improvements in their daily lives.

* In total:

The program includes a total of three 15-minute sessions per week for eight weeks and involves :

<u>For Children</u>

- One medical visit and a neuropsychological examination at inclusion ,
- 2 neuropsychological tests after the intervention (8 months, and 18 months after the inclusion and ,
- The 8-weeks CT program.

<u>For Parents</u>

- An 8 weeks monitoring program,
- A weekly phone call,
- Delivering the questionnaire to the teacher at the inclusion and after eight months,
- Filling out self administered questionnaires and answering to a face to face questionnaire (at inclusion and eight months).

<u>For teachers</u>

Completion of the questionnaire at the inclusion and 8 months later.

4.42 Control group: current standard care management

The rehabilitation program will not be offered to this group. The children will be followed up along with their routine care management. Speech therapy and/or academic support may be recommended for those experiencing academic difficulties. The visits are the same for the children and the questionnaires are the same for parents and teacher than in the experimental group.

4.43 Total participation time
The total participation time is 18 months for both the experimental and control groups.

4.5 ENDPOINTS

4.51 Primary endpoint
The visual-spatial processing will be assessed by using the visuo-spatial index (VSI) of the Wechsler Preschool and Primary Scale of Intelligence (WPPSI IV). This index consists of two subtests : block design and object assembly. The average score is 100 with a standard deviation of 15.

Rationale for the primary endpoint:

Visuo-spatial performance is a complex mental process requiring multiple mental functions: attention, motor sensory and executive functions and WM (visuo-spatial and verbal).

Premature children perform less well to visual perceptual integration testing than those born full term and they present with a visuo constructive dyspraxia. This deficit is connected to a poor integration of visual function, perceptual and/or fine motor skills (reproduction of a complex geometric figure). This disorder is three times more frequent in adolescent very prematurely born than in adolescents born at term. Among those born extremely prematurely, 30% have results below the 15th percentile in visuo-spatial performance. (Torrioli MG 2000). The impact of WM rehabilitation on visuo-spatial skills is an interesting line of research, most particularly in premature infants.

4.52 Secondary endpoints
The secondary endpoints are related to the children and to their parents.

4.521 Children's endpoints
- The intellectual functioning and other cognitive processes will be obtained by global IQ and IQ indexes using the WPPSI IV (Wechsler) (Marley W 2014).

The WPPSI IV, designed for children ages 4 to 7, assesses the overall intellectual functioning (total comprehensive intellectual quotient : TCIQ) and specific cognitive processes. This is done through the main indices: verbal comprehension index (VCI), fluid reasoning index (FRI), visual spatial index (VSI), processing speed index (PSI), and the Working Memory index (WMI). The evaluation of the Working Memory by the WMI in WPPSI IV is uniquely visuo-spatial at this age. The average is 100 with a standard deviation of 15, as with all Wechsler Scales.

The global IQ, as well as the main indices, will be assessed when monitoring the EPIPAGE cohort.

The global IQ is lower for very premature infants as opposed to those with full-term.(AndersonPJ 2014)

- Executive and attention processes

NEPSY 2 assesses the neuropsychological development of preschool and school age children (3-12 years) and is used to obtain emerging executive functions in 5-6 year old (Korkman M 2001).

Auditory attention, statue, and design fluency are the only three subtests in the Executive and Attention Function domains, which will be administered in this study. These three tests measure selective and divided attention in auditory modality, inhibition and mental flexibility.

A review of Van de Weijer-Bergsma, which is confirmed by Mulder in a meta-analysis, shows that the selective, divided and supported attentional domains are likely to be affected in preschoolers that were prematurely (Van de Weijer-Bergsma E 2008, Mulder H 2009).

- Evaluation of language and its skills

Language is a complex mental process requiring an assessment of all its components. This assessment will be made from the Clea battery (ECPA) calibrated for ages 2-15 years (Charollais A 2014)

The battery consists of seven tests:

1 Known Digital Channels (KDC) : a reflection of verbal learning ability, both cultural and cognitive,
2 Oral Word Identification (OWI) : capacity to give a word phonological and semantic judgment;
3 Rapid Denomination (RD) : timed tested; gives an indication treatment speed,
4 Word memory (WoM) : explores the short-term mnemonic span,
5 Facial and oral praxis photographs (Prax),: relevant in motor programming,
6 Visual attention (VAtt) : inspired from NEPSY, it questions the visual spatial component,
7 Resolution of logical problems (PR) : ability to reason analogically (progressive matrices).

Many studies suggest that specific language disorders can be associated to specific WM impairment, particularly with the phonological loop (Adams AM 2000, Rodriguez A 2009).

Two meta-analyses demonstrate global language gaps/impairment in the very premature vs. the term child with deficits in learning ability, phonology, semantics, grammar, speech coherence and verbal reasonin. (Van Noort-van der speck IL 2011, Reidy N 2013).

- Behavioral Evaluation

The child's behavior will be assessed with the Goodman Strengths and Difficulties Questionnaire, which includes 25 questions answered by the parents (Goodman R 1999), in a self administered questionnaire. It will assess whether or not there is any impact of the intervention on the child's behavior.

- Evaluation of the child's quality of life (QOL)

The quality of life of the children will be assessed using the Perceived Quality of Life and Health of Adolescents and Children Questionnaire (VSP-A) (Auquier P 2001) as reported by the parents. The children's QOL will be reported by their parents.

The 49-item version for children (VSP-Ap) portrays nine dimensions and index:

- relationships with parents/family,

- body image,
- vitality,
- relationships with friends,
- general well-being,
- leisure,
- school performance
- relationships with teacher
- relationships with medical staff.

Scores range between 0 and 100, with higher scores indicating a better QOL. The French norms are available through Ravens-Sieberer U 2007.

-Schooling

Schooling will be evaluated by the GSA questionnaire (Global School Adaptation score), a French tool completed by the teacher, validated by Guimard P (2007) and re-evaluated in a preschool population in 2013 (Boussicault G 2013).

The questionnaire covers six verbal skills(verbal communication, verbal participation, vocabulary, syntax, pronunciation), five non-verbal abilities (memory, arithmetic, logical reasoning skills, manual dexterity and fine motor skills) and eight questions evaluating class behavior (compliance with rules, attention, autonomy, speed of accomplishing the task, self-esteem, ability to keep the pace and fatigability). The final question asks the teacher about possible future special educational needs of the child.

4.522 Parents'endpoints

Anxiety

The Spielberger state-trait anxiety inventory (STAI) will be used to assess anxiety. The STAI is a self-reporting questionnaire consisting of 40 items that measure both the state and trait scores. These scores range from 20 (absence of anxiety) to 80 (high anxiety) (Spielberger C 1983). This questionnaire will assess if anxiety is impacted as a result of parental intervention (mother).

Rationale for the secondary endpoints :

It is of value to measure the impact of intervention on the Working Memory and on other non-trained brain processes as well as on parental anxiety, child behavior and parental perceptions of the quality of life 'child.

V GENERAL FRAMEWORK

5.1 STEERING COMMITTEE

A steering committee will be composed of Dr Gire as coordinating investigator, two co-investigators, the EPIPAGE 2 responsible-in-chief (Dr Ancel), and a methodologist (Dr Baumstarck). The committee will assure the quality of the processes, the validation of the various steps of the study; the identification of potential study dysfunctions and means of remediation as well as the implementation of problem remediation.

5.2 IDENTIFICATION OF ELIGIBLE CHILDREN

5.21 Identifying eligible patients will be obtained during the EPIPAGE 2 group follow-up by WPPSI IV : total IQ >70 and IWM <85

* All children from the Epipage group will benefit from :

- A neuropsychological examination between the ages 5 years 6 months and 5 years 9 months. This examination will include :
    - The WPPSI IV with its main indexes,
    - Some NEPSY subtests to evaluate executive functions (inhibition, mental flexibility), social perceptions, visual-motor precision and language.

  This assessment will be made by a neuropsychologist and will last about 120 minutes.
- A medical examination including :
    - Child's health and treatment history,
    - A neurological and medical examination.

* All parents will be asked to complete the following questionnaires:

- SDQ: Child behavior,
- BRIEF: executive functions,
- SCQ (Social Communication Questionnaire),
- Autism spectrum disorders.

To ensure that the questionnaires will be properly completed, they will be checked by the regional or local coordinator accompanying the parents during the examination.

The evaluation will be carried out for research rather purposes as far as possible, testing time will be respected, and will not take more than half a day.

The tests will be performed in a predefined order, so as to homogenize testing conditions. Evaluation results and a brief written report will be presented to the parents on the examination day, with a more complete report sent by mail after discussion by the local team. The elements to be included in these reports will be discussed again. Time for test readings, scoring and report writing will be integrated in the doctors' and psychologists' remuneration.

This very comprehensive assessment will identify those children eligible for EPIREMED, that is, with a WM index lower than 85, having no exclusion criteria and whose family is able to travel to the participating testing centers.

5.22 Patient contacts

There will be a medical consultant identified at each center to optimize the implementation and the running of the study and will be the liaison between the principal medical investigator and clinical research coordinator.

The clinical research coordinator will establish a list of potentially eligible patients per month/for center in conjunction with the study national coordination. Using this list, each center's referring physician will organize the first telephone interface with those families having contact information and eligible criterion. The study's objectives, those responsible for the study, its development, and its advantages and disadvantages, will be explained. Reimbursement for travel expenses will be routinely offered. Once the family is in agreement, an appointment will be scheduled.

The family will receive a confirmation letter describing all topics discussed by telephone, the appointment date. Telephone contact will be made within two months after the identification in EPIPAGE 2 and an appointment will be set within two months.

Each center will survey the actual inclusion among all potentially eligible children (WM <85), and validate the reasons for not including the other children.

5.3 BASELINE ASSESSMENT AND INCLUSION

Eligible Epipage children with parental consent will be included after a new baseline assessment :

1. A complementary neuropsychological examination by the neuropsychologist who has done the EPIPAGE 2 evaluation which includes:
    - Auditory attention (NEPSY 2),
    - Language skills (ECLA battery).

2. Parents will complete the following questionnaires, and the regional or local coordinators accompanying parents will check their completion.
    - Parental anxiety (Spielberger questionnaire [STAI]),
    - Their children's QOL : (VSPAp)

3 A medical consultant will meet with the parents and children to ensure the criteria of inclusion and will re-explain the study.

5.4 RANDOMIZATION

A randomization list will be established before the implementation of the study with a 1:1 allocation ratio, and will be elaborated by the Clinical Research Platform, APHM (Pr Pascal Auquier).

A computer-generated randomized list using a permuted block design will be done (stratified on center and gemellarity, singleton/twin). Each center will have a specific list and inform the coordinating center of each inclusion. The inclusion form, including its date, the date of randomization, and the patient's initials will be faxed or e-mailed to the coordinating center. This list will be updated at each inclusion with the randomization date will determining the different delays.
Randomization is centralized and balanced by each center. Multiple births represent 30% of the preterm population within the EPIPAGE 2 group. If one twin has a WM anomaly, this child will be

selected for randomization. If both twins have a diminished WM; we will randomize one of the two twins in the study and the same care will be offered to the other twin.

5.5 FOLLOW UP FOR THE TWO GROUPS

Children in the Cogmed JM group will begin the program no later than two months after their inclusion, and children in the standard group will receive the routine management. A similar follow up is planned for the two groups and is based on an intermediate and final visit.

5.51 The 8 month intermediate visit

An assessment will be conducted eight months after being included in the study and will consist of :

1. The children :

- Assessment of the working memory index (WMI),
- IQ and its main indices (Weschler),
- Statue, design fluency and auditory attention : The NEPSY 3 subtests,
- Language and verbal skills (ECLA).

2. The parents:

- A self-administered questionnaire on anxiety (SDQ),
- A questionnaire assessing the behavior of the child (Goodman),
- A questionnaire assessing the child's QOL (Vspa P).

A blind evaluation will be performed by the neuropsychologists at the different EPIREMED participating centers with the group allocation of each child being unknown to the assessors.

5.52 The -18 month final visit

Children in both the intervention and control groups will receive a final evaluation 18 months after inclusion when they will be between 7 and 7 and ½ year old. A blind evaluation will be performed by the neuropsychologists at the different EPIREMED participating centers with the group allocation of each child being unknown to the assessors.

The primary evaluation end point:

The visuo-spatial Index WPPSI IV (Wechsler Preschool and Primary Scale of Intelligence) will be used for this visit. The global IQ, its indices including WMI will also be used to determine the long-term maintenance of working memory (WM) and its impact on intelligence.

VI DATA MANAGEMENT AND STATISTICAL ANALYSIS

6.1 SAMPLE SIZE

The sample size was calculated to obtain an 80% power to detect a difference of 7.5 points on the VSI index (estimated standard deviation: 15) at 18 months between the 2 groups. This has been considered to be clinically significant considering previous similar studies (Anderson PJ 2015). With the threshold for statistical significance set at a p-value of 0.05, assuming that a potential 15% of patients will be lost to follow-up between baseline and last assessments, these calculations showed that 144 patients are needed (72 per group) (Power Analysis and Sample Size Software Version 2008, Utah, USA). A total of 150 individuals will be included, 75 for the control group and 75 for the experimental group.. Assuming that 30-40% of the EPIPAGE 2 children will present with a WM abnormality, i.e., 1,600 children, recruitment should be ensured. In keeping with the highly restrictive selection criteria previously defined (see inclusion and non-inclusion criteria), recruitment is proposed as follows:

Investigators	Center	Average Expected recruitment per month	Expected recruitment
Gire Catherine	Marseille France	1,5	16
Garcia Patricia	Marseille France	1,5	16
Claris olivier	Lyon France	0,6	8
Hascoet Jean Michel	nancy	0.6	8
Patural Hughes	St Etienne France	0,6	8
Roze jean Christophe	Nantes France	0,6	8
Maillotte Anne marie	Nice	0.6	8
Marret stephane	Rouen France	0,6	8
Saliba Elie	Tours France	0,6	8
Guillois Bernard	Caen France	0,6	8
Lecomte Benedicte	Clermont-Ferrand	0.6	8
Cambonie Gilles	Montpellier France	0,6	8

Kuhn Pierre	Strasbourg France	0,6	8
Bednarek Nathalie	Reims France	0,6	8
Granier Michelle	Paris France	0,6	8
Foix-hellias Laurence	Paris France	0,6	8
Mitanchez delphine	Paris France	0,6	8
Debillon Thierry	Grenoble Frange	0.6	8
Arnaud Catherine	Toulouse France	0.5	8
Souksi Medioni Isabelle	Montpellier France	0.5	8
TRELUYER Jean-Marc	CIC Necker APHP	0.5	8
Total			184

6.2 STATISTICAL ASPECTS

Although the analysis' main principles are reported below, a second specific protocol will be provided before the study's initialization. This will be validated by the coordinating investigator, those responsible for the analysis and the biostatistician.

Statistical analysis will be carried out by Pr. Pascal Auquier (Dr. Karine Baumstarck and Anderson Loundou and their team [Clinical research unit, DCR, APHM]). Data will be analyzed using SPSS version 17.0 software. Statistical significance is defined as p<0.05.

The methodology will be based on the Consolidated Standards of Reporting Trials Statement (CONSORT, http:// www.consort-statement.org/consort-statement/)(Schultz KF 2010).

The full analysis which includes all randomized subjects will be used in the primary analysis. The per protocol population (including all randomized subjects with no major protocol deviations) will be used in the secondary analysis to assess the robustness of the results. There is no interim analysis planned.

In each center the actual inclusions will be identified among all potentially eligible children (WM <85).

Demographic and baseline characteristics will be summarized. Quantitative data will be shown as mean ± standard deviation or median with its interquartile range. The qualitative data will be described as percentages with a 95% confidence interval. These data will be compared between the "control" and "experimental" groups using the chi2 test for qualitative variables and the Student t tests for continuous variables.

6.21 Primary endpoints analysis

The analysis of the primary and secondary endpoints will be done with the intent to treat. All tests used will be bilateral with a 5% significance threshold.

The mean scores of the VSI index will be compared between the 2 groups (Student t test or Mann Whitney test). Linear regression will be performed to adjust for potential confounding factors; variables relevant to the models will be selected on their clinical interest and/or a threshold p-value <= 0.1 during bivariate analysis. The final models expressed the beta standardized. The unadjusted analysis will be the primary analysis, and the adjusted analysis will be a complementary analysis.

6.22 Methodology to account for missing data

Children not meeting the primary endpoint measurements will be considered as a failure in the study regardless of their randomization group. Additional analyses will be conducted based on:

1) Available data and,

2) After multiple imputation of missing data.

6.23 Analysis of secondary endpoints

The different indices mean scores of the WPPSI, NEPSY, Clea, SDQ and GSA will be compared between the 2 groups using a Student t test or Mann-Whitney test in accordance with the variables' distribution. Anxiety and QOL scores will be compared between the 2 groups.

The comparisons of percentages will be done with the Pearson Chi-2 test or, if needed, with the Fisher exact test. All tests used will be bilateral with a significance threshold of 5%.

The scores at baseline and 8 and 18 months respectively were compared, and the analysis of variation for repeated measurements will be performed for the two groups to compare changes over time (baseline, 8 and 18 months).

6.24 Patient selection to include in the analysis

All the children registered and randomized in the study will be included in the analysis, respecting the intention to treat. This analysis will be conducted, if necessary, under the maximum bias hypothesis. In second step those wrongly included and any major protocol deviations will be excluded from the analysis.

VII ORIGINALITY AND INNOVATIVE ASPECTS

Our proposed study has different innovative levels:

- EPIPAGE 2, the national prospective cohort of very premature children offers an interesting and unique setting for the implementation of a randomized trial of CT program (EPIREMED) in premature preschoolers. This cohort provides high quality data on the perinatal period, the socio-demographic context and the child's cognitive future.

-EPIREMED is the largest, randomized, interventional study performed on the WM of a very premature birth population.

-EPIREMED, by using Cogmed JM, an on-line computerized program for WM rehabilitation, allows the parents to participate in the child's rehabilitation along with other major development actors

-EPIREMED by using Cogmed JM offers a method to improve the visual WM and possibly other untrained brain skills. Considering the cost to society and the personal investment asked of the parents, it is necessary to prove there is a high level response to this intervention. If the trial is conclusive, it would enable development of a prevention strategy by confirming one of the mechanisms associated with premature birth cognitive disorders.

-EPIREMED, which is nested in the EPIPAGE 2 study, has a follow-up until ages 8-12 years, allowing us to assess the long-term outcome for the child's cognitive performance, behavior and education. Indeed there is a need for scientific evidence regarding the effectiveness of a well-described and applicable intervention in order to improve the medium/long-term future of very premature children. This will enable us to obtain information to improve our knowledge of WM natural history of development in premature children and to identify alteration risk factors.

VIII EXPECTED FINDINGS AND INDIVIDUAL/COLLECTIVE POTENTIAL BENEFITS

This project's primary goal is to demonstrate the necessity of early visuospatial VM assessment within the vulnerable population of VP children, and to prove the feasibility and efficacy of computerized CT using online software programs.

Neurodevelopmental problems are common in VP children. Recent publications have reported many disorders in their specific cognitive functions, one of which is WM. Although neuro-development of VP children remains a public health priority because of their increased birth and survival rates, the interventions to improve this issue remain very few. There are currently no truly effective interventions to deal with academic achievement in preschool-age very preterm infants and their neuropsychological problems; thus, currently no specific management care is recommended for these children.

Cogmed is based on interactive software and parental support. The program takes into account the child's environment, a factor of great relevance since it is closely linked to the child's future development. The expected benefits of CT (Cogmed) for those children in this study are enhanced WM, possibly leading to better EF by taking advantage of cerebral plasticity. A better global neuropsychological development improvement (language and visuospatial processing) can be expected with an improvement in learning and decreased behavioral problems. For parents, their guidance is required in Cogmed and can reduce their anxiety by fully embracing their role as primary agents in their child's development. This is consistent with recommendations for family-centered healthcare and can significantly improve the quality of life in both the children and their parents. In the long term, these improvements might also reduce those global costs linked to the consequences of extreme prematurity.

Finally, if proved effective for this vulnerable population, this treatment can be a possible option or alternative in any other preschool population complaining of early academic difficulties related to WM deficits.

IX CALENDAR

The recruitment duration is estimated to be 18 months depending on the selection criteria and recruitment capacity of the centers. The maximum participation for each patient will be 18 months. The main steps of the study are presented in the following table.

Date	Action
January 2016	Legal authorizations: CPP, ANSM (French authorities)
February 2016	Investigators' meeting:<br>Validation of the study documents (CRF, consents, randomization lists)<br>Preparation for the implementation of the study within the centers<br>- Training of the neuropsychologists
October 2016	First inclusion
January 2018	Last inclusion
June 2019	End of the follow-up
July-December 2019	Quality control and data entry
January to Novembre 2020	Statistical analysis<br>Final report
May 2020	Investigators meeting:<br>Validation of the results of the final analysis<br>- Organization of the data publication

X PROJECT FAISABILITY

10.1 PARTICIPATION OF A RESEARCH NETWORK

EPIREMED is part of a research network along with EPIPAGE 2 that has already partnered in nine complementary projects (Ancel PY 2014) and is involved in the European project EPICE and the French national children cohort, ELFE.

10.2 OTHER ELEMENTS OF PROJECTS FEASIBILITY

This study is based in the EPIPAGE 2 cohort: 4,200 children, with a follow-up rate of 90 and 87% at 1 and 2 years respectively.

Families were recontacted when the child was 3 year old and will be again contacted at age of 4 (2015) to update addresses.

The study's feasibility is high since patients have already been monitored and identified and EPIREMED centers are designed to diagnose WM disorders up until 5 years and ½ of age.

EPIPAGE 2 provides an extensive neuropsychological evaluation at 5 years and ½, which serves as a prescreening. Cogmed JM has already been used in other contexts and in numerous randomized studies (Sodervisqt S 2015).

Three randomized and controlled studies similar to our population (ie preschool children) showed the effectiveness of WM rehabilitation with sustained effects and untrained brain function improvement (Nutley SB 2011, Thorell LB 2009, and Sodervisqt 2013).

Finally, the implementations by parents, key players in the child's development, along with the playful interactions with computers, which are age-appropriate, provide an assurance of compliance which can result in a successful rehabilitation.

10.3 TEAM EXPERIENCE IN THE FIELD

10.31 Coordinating investigator : C Gire

As a neonatologist and pediatric neurologist, Dr. Gire has been involved in neurological development for several years. Dr. Gire works in neonatology and is co-director of the women's parent-child center at *Hôpital Nord at AP-HM (Assistance Publique, Hôpital de Marseille*). Dr. Gire has been affiliated for several years with UPRES - EA 3279, Public health, chronic diseases, quality of life, concepts, applications and limitations, and determinants.

This team works in two main areas:

- Conceptual and methodological aspects underlying quality of life measurements in chronic diseases,
- The role of these measures in maternal and child health; mental health and its precariousness, oncology, and renal Insufficiencies.

Currently she is the principal investigator of PHCR 2009: "Quality of life of very pre-term children during elementary school": Six Level III centers (Marseille [2], Montpellier, Nantes, Nimes, and Rouen) are included in the study of 7 to 9 year old, each with an informed consent. The children were born between 1/1/2004 and 31/12/2007 before the 28th week of gestation and none with any

severe PC and/or mental deficiencies. At the end of a specific consultation the following items are identified for all participating children: QOL data (reported by children and their parents), clinical examination of the child according to “Touwen” protocol, comprehensive cognitive profile (WPPSI IV, NEPSY [executive function and focus], figure REY). The study began in 2012 and will continue until December 2015. In March 2015, the 27th month of the study, 490 parents were contacted (80% of our active files). Among these 490, 347 parents (71%) gave their consent to participate. Dr. Gire is thus expert in the organization of a multicenter study with neuropsychological assessment of children.

Additionally, she is the coinvestigator of an ancillary EPIPAGE project: EPIRMEX. This project focuses on different aspects of conventional or advanced brain MRIs at term (39-41 weeks) in a sub sample of very preterm infants included in EPIPAGE 2,, and its relationship with executive functions up to 5 years et 1 /2 of age. The high number of MRIs performed in our region (130 out of 540) is testimony to Dr. Gire’s strong involvement in this study.

10.32 Science Team

10.321 Baumstarck Karine

Dr Karine Baumstarck is a physician specialized in Public Health. She works in a Hospital University Center (CHU, Assistance Publique, Hôpitaux de Marseille, France) in the Clinical Research Unit as methodologist. She supports different investigators in the structuration of their clinical research projects. She also is affiliated to a University Department (Aix Marseille University, France), EA 3279, Quality of life and chronic diseases, responsible Prof. Pascal Auquier.

10.322 Kaminski Monique

Dr. Kaminski, Director Emeritus of research at INSERM’s EPOPÉ team, UMR 1153, is an epidemiologist in perinatal and children’s health; and works on the long-term development of very premature children. Dr. Kaminski was involved in implementation and data analysis of such major chort study as EPIPAGE 1, EDEN, and is currently a member of the EPIPAGE 2 scientific committee.

10.323 Ancel Pierre Yves

Dr. Ancel is MCU-PH (*Maître de conférence des universités – praticien hospitalier*) at the University Paris V; Director of the Obstetric Epidemiology Team, Perinatal and Pediatric, EPOPé, UMR 1153 Inserm. Dr. Ancel is a perinatal care epidemiologist and a participant in the EPIPAGE 1 cohort as well as the manager/national coordinator of EPIPAGE 2.

10.324 Calderon Johanna

Calderon Johanna neuropsychologist is responsible for a Boston study that is assessing the Cogmed program on children with cardiac malformations

10.33 Investigators and associated members of the steering committee EPIPAGE 2

The EPIREMED study is part of EPIPAGE 2 and allows us to benefit from the collaboration of those involved in the EPIPAGE 2 cohort's steering committee. The different investigators are involved in the monitoring and care of very premature babies. Their diverse training and responsibilities meet all aspects of our project. These investigators are either pediatrician-neonatologists or pediatric neurologists working in type III neonatal units, in neuro-pediatric services for very premature infants’s assessment or in a referral center for learning disabilities.

10.4 FINAL REPORT AND PUBLICATION

In accordance with Article R 5121-13 of the Public Health Code, clinical studies prior to their publication are not subject to any written or oral comments without the joint agreement of the coordinating investigator and the promoter.

Any publication must state that APHM is the promoter and the study's databases will be co-owned by the promoter APHM and by the INSERM U1153, EPOPÉ team. The results, as well as all research-related data, must under no circumstances be transmitted to third parties without compensation negotiated beforehand by the Medical Research Branch of the APHM and/or INSERM U 1153

All data, results, inventions and discoveries resulting from the study will automatically become the exclusive property of the sponsor which may use this information in the manner deemed suitable with the agreement of the investigator.

Publication requirements: all results of an APHM funded clinical study or a request for proposal is under the scientific responsibility of the coordinating investigator, and these results must be made public if they have sufficient scientific validity. Any request to hide the results, change and mitigate the contents of the final report and publication will be rejected by the promoter. Publications must describe in an honest and balanced way all aspects of the study regardless of other interests, particularly those of the non-scientists. The publications' authors must comply with the quality rules of scientific journals.

10.5 CASE REPORTS FORM

All information collected by the overseeing practitioner will be transcribed in the case report. The data, whether clinical or non-clinical, will be transferred as the data are obtained.

XI REGULATORY AND ETHICAL ASPECTS

11.1 APPROVALS

The expected sponsor is represented by the *Assistance Publique-Hôpitaux de Marseille* and must have approvals from the French authorities prior to the study's initiation. The sponsor and the investigators will conduct the study in accordance with Good Clinical Practices and the French applicable regulatory requirements (*Code de la Santé Publique*, article L.1121-1/Public Health Act No. 2004-806 of 9 August 2004 on public health policy and its implementing decrees of August 27, 2006), as the applicable privacy requirements and ethical principles outlined in the Declaration of Helsinki. The protocol and amendments will be approved by the French *Comité de Protection des Personnes* (Protection to Persons committee) and by the *Agence Nationale de Sécurité des Médicaments.* (national agency for drug safety). Participation is voluntary and all patients will submit a written informed consent. Protection of the rights will be guaranteed, and anonymity conserved. The study's results will be in the form of feedback to the patients.

11.2 CONFIDENTIALY

In accordance with French laws and regulations respectively (Law of August 9, 2004, Computer Law and Liberties of January 6, 1978, as amended by the Act of 1 July 1994 and Decree of May 9, 1995) and (National Data Processing and Liberties Commission and the Advisory Committee on the treatment of research information in the health field: Decision of 5 January 2006. Reference Methodology MR-001)): all patient records identities will remain confidential.

11.3 OTHER REGULATORY ASPECTS

Management of protocol amendments and adverse events will follow the regulatory procedures of the sponsor.

11.31 Monitoring and quality control

In accordance with applicable regulations including Good Clinical Practices, and Sponsor's procedures, a Sponsor's monitor will visit the investigator before, during and after the study. Sponsor's monitors will contact the site prior to the start of the study to review with the site staff the protocol, study requirements, and their responsibilities to satisfy regulatory, ethical, and Sponsor's requirements. When reviewing data collection procedures, the discussion will also include identification, agreement and documentation of data items for which the CRF will serve as the source document.

Sponsor will monitor the study and site activity to verify that the:

- Data are authentic, and complete.
- Safety and rights of subjects are being protected (obtaining consents and recording prior to their participation in the trial).
- Study is conducted in accordance with the currently approved protocol and any other study agreements, GCP, and all applicable regulatory requirements.

The investigator and the head of the medical institution agrees to allow the monitor direct access to all relevant documents

The type and frequency of monitoring are determined using the definition of the level of monitoring based on patient risk and depend on the number of patients included, the rate of inclusions and difficulties encountered during the conduct of the study (procedures approved by the group quality promotion FHF which determines the level of monitoring to be carried out based on risk to the subject-OECD recommendation on the Governance of Clinical Trials, December 2012).
In this study, the level of monitoring is rated “minimal” with a risk for the patient level A. The Sponsor’s monitor will verify the signed informed consents. If one / multiple consents are not compliant, files will be monitored randomly.

The protocol’s study information will be posted on **clinicaltrials.gov** before subjects are enrolled.

11.32 Vigilance of clinical trials

Definitions

Adverse event (AE)

Any noxious or undesirable event experienced by a participant during a clinical trial, whether or not considered related to the protocol should be considered as an adverse event.

Serious adverse event (SAE)

A serious adverse event is an adverse event that is:

- Fatal
- life-threatening
- significantly, persistently or permanently disabling
- requiring in-patient hospitalization or prolongation of hospitalization
- requiring intervention to prevent permanent impairment or damage
- Any event that could be considered as potentially harmful
- Any event medically accurate according to the investigator’s judgment

In addition, congenital abnormalities, occurrence of malignancy or clinical injuries resulting from overdose are always considered as SAE.

Responsibilities of the investigator

Modalities of collection, verification and presentation of adverse events

All adverse events occurring during clinical trials have to be collected, verified, registered and reported from Day 0 (inclusion day for the participant) until the last day of the study, or as soon as the investigator becomes aware of the AE that he considers linked to the protocol and this up to its resolution.
Adverse events are collected:

- During clinical examination, from blood sample analysis, or from the investigator questioning the patient
- from the patient’s unsolicited reporting, as encouraged to do towards the investigator

Serious adverse events reporting (SAE)

The investigator must assess the SAE in severity and report it to the sponsor within 24 hours of his/her becoming aware of it (SAE Form at the end of the CRF).

The investigator must search for evidence of a causality between the protocol and the SAE, and supply when possible a medical prognosis. The investigator must provide information on symptoms, time of onset, subsidence, action taken and subject outcome.
The SAE Form must be sent to the sponsor together with the hospital reports, examination reports and biological results related to the SAE (including negative results); Single patient reports must be anonymised and bear a code/randomization number.
Once completed, dated and signed, the Serious Adverse Event Form must be addressed by fax to:

Direction of Clinical Research and Innovation of AP-HM<br>80, rue Brochier, 13354 Marseille Cedex 05<br>Phone 04 91 38 27 47 Fax: 04 91 38 14 79<br>E-mail: drci@ap-hm.fr

All subjects with SAE must be followed up for outcome until resolution (even if the patient dropped out/was excluded from the study); reports must be sent to the sponsor within 8 days of the initial SAE declaration by fax or email.

Causality link
The investigator must assess the causality link between the SAE and the protocol. If a causality link is suspected, the investigator/sponsor will regard the SAE as a Suspected Unexpected Serious Adverse Reaction (SUSAR).

Declaration period
SAEs must be reported for a participant:
- From the day he signed the consent form
- For the duration of the follow-up as defined in the study protocol
- Up to 4 weeks after the end of the study protocol
- Anytime, as soon as the investigator becomes aware of it and if a causality link has been evidenced between the protocol and the SAE.

<u>Responsibilities of the sponsor</u>
Unexpected SAE declaration
The sponsor must assess the causality link between the SAE and the protocol.
The sponsor will also report all expected/unexpected SAE based on the SPC (Summary of Product Characteristics).
The sponsor will assume the responsibility for appropriate reporting of adverse events to Ethics Committees and relevant Health Authorities.
The regulatory delays for SAE declaration are:
- 7 days for SAE, fatal SUSARs or life-threatening SUSARs. A further delay of 8 days is granted for full and accurate documentation of the case
- 15 days for all other SUSARs. A further delay of 8 days is granted for full and accurate documentation of the case
- In double-blinded studies, the sponsor must report the SUSAR to the relevant Health Authorities and Ethics committees as soon as possible after the unblinding visit.

New Safety Data declaration

The sponsor must declare any new safety data and send an Annual Security Report to the relevant Health Authorities and Ethics committees.

Annual Security Report

At the date of the anniversary for the trial authorization issued by Health Authorities and Ethics Committees, the sponsor must write a safety report including:

- The list of serious adverse event that may be linked to the protocol including unexpected and expected serious events.
- A critical assessment of patient safety suitable for research.

This report can be submitted for approval to the principal investigator.

The sponsor must send the Annual Security Report within 60 days of the trial authorization.

Data Safety Monitoring Board

A committee of Data and Safety Monitoring Board will not be formed for this protocol.

XII REFERENCES

Aarnoudse-Moens CS, Weisglas-Kuperus N, van Goudoever JB, Oosterlaan J. Meta-analysis of neurobehavioral outcomes in very preterm and/or very low birth weight children. Pediatrics. 2009 Aug;124(2):717-28

Adams AM, Gathercole SE. Limitations in working memory: implications for language development. Int J Lang Commun Disord. 2000 Jan-Mar;35(1):95-116

Alloway TP, Alloway RG Investigating the predictive roles of working memory and IQ in academic attainment.J Exp Child Psychol. 2010 May;106(1):20-9

Alloway TP, Gathercole SE, Pickering SJVerbal and visuospatial short-term and working memory in children: are they separable? Child Dev. 2006 Nov-Dec;77(6)

Ancel PY, Goffinet F; EPIPAGE 2 Writing Group .EPIPAGE 2: a preterm birth cohort in France in 2011.. BMC Pediatr. 2014 Apr 9;14:97

Ancel PY, Goffinet F; EPIPAGE-2 Writing Group, Kuhn P, Langer B, Matis J, Hernandorena X, Chabanier P, Joly-Pedespan L, Lecomte B, Vendittelli F, Dreyfus M, Guillois B, Burguet A, Sagot P, Sizun J, Beuchée A, Rouget F, Favreau A, Saliba E, Bednarek N, Morville P, Thiriez G, Marpeau L, Marret S, Kayem G, Durrmeyer X, Granier M, Baud O, Jarreau PH, Mitanchez D, Boileau P, Boulot P, Cambonie G, Daudé H, Bédu A, Mons F, Fresson J, Vieux R, Alberge C, Arnaud C, Vayssière C, Truffert P, Pierrat V, Subtil D, D'Ercole C, Gire C, Simeoni U, Bongain A, Sentilhes L, Rozé JC, Gondry J, Leke A, Deiber M, Claris O, Picaud JC, Ego A, Debillon T, Poulichet A, Coliné E, Favre A, Fléchelles O, Samperiz S, Ramful D, Branger B, Benhammou V, Foix-L'Hélias L, Marchand-Martin L, Kaminski M. Survival and morbidity of preterm children born at 22 through 34 weeks' gestation in France in 2011: results of the EPIPAGE-2 cohort study. JAMA Pediatr. 2015 Mar;169 (3):230-8

Anderson PJ .Neuropsychological outcomes of children born very preterm. Semin Fetal Neonatal Med. 2014 Apr;19(2):90-6

Anderson PJ, Doyle LW; Victorian Infant Collaborative Study Group Executive functioning in school-aged children who were born very preterm or with extremely low birth weight in the 1990s.Pediatrics. 2004 Jul;114(1):50-7

Anderson PJ, Reidy N.. Assessing executive function in preschoolers. Neuropsychol Rev. 2012 Dec;22(4):345-60

Auquier P, Clement A, C Sapin, El Khammar M, San Macro JL, Siméoni MC: Development, validation of HRQL measurement in children: VSP-Ae. Qual Life Res 2001, 10: 268

Auquier P, Clément A, Sapin C, El Khammar M, San Marco JL, Siméoni MC: Development, validation of HRQL measurement in children: VSP-Ae. Qual Life Res 2001, 10:268

Aylward GP Cognitive and neuropsychological outcomes: more than IQ scores. Ment Retard Dev Disabil Res Rev. 2002;8(4):234-40

Baddeley A. The fractionation of working memory. Proc Natl Acad Sci U S A. 1996 Nov 26;93(24)

Baron IS, Erickson K, Ahronovich MD, Litman FR, Brandt J. Spatial location memory discriminates children born at extremely low birth weight and late-preterm at age three. Neuropsychology. 2010 Nov;24(6):787-94

Bergman Nutley S, Söderqvist S, Bryde S, Thorell LB, Humphreys K, Klingberg T Gains in fluid intelligence after training non-verbal reasoning in 4-year-old children: a controlled, randomized study. Dev Sci. 2011 May;14(3):591-601

Bhutta AT, Cleves MA, Casey PH, Cradock MM, Anand KJ Cognitive and behavioral outcomes of school-aged children who were born preterm: a meta-analysis. JAMA. 2002 Aug 14;288(6):728-37

Bierman Kl , Torres M . Promoting the development of executive functions though early education and prevention programs. In J.A Griffin LS. Freund and P McCardle (eds) Executive function in preschool age children: integrating mesasurement , neurodevelopment and translational research. Washington , DC : American psychological Associataion (2015)

Boivin MJ, Busman RA, Parikh SM, Bangirana P, Page CF, Opoka RO, Giordani B. A pilot study of the neuropsychological benefits of computerized cognitive rehabilitation in Ugandan children with HIV. Neuropsychology. 2010 Sep;24(5):667-73
Borella E, Carretti B, Riboldi F, De Beni R. Working memory training in older adults: evidence of transfer and maintenance effects .Psychol Aging. 2010. Dec;25(4):767-78
Borradori Tolsa C, Barisnikov K, Lejeune F, Hüppi P.Development of executive functions in preterm children]. Arch Pediatr. 2014 Sep;21(9):1035-40
Borteyrou X, Bruchon-Schweitzer M, Spielberger CD. [The French adaptation of the STAXI-2, C.D. Spielberger's State-trait anger expression inventory]. Encephale. 2008 Jun;34(3):249-55
Boussicault G, Nguyen The Tich S, Branger B, Guimard P, Florin A, Rozé JC, Flamant C.The Global School Adaptation score: a new neurodevelopmental assessment tool for very preterm children at five years of age. J Pediatr. 2013 Aug;163(2):460-4
Boyle MH, Miskovic V, Van Lieshout R, Duncan L, Schmidt LA, Hoult L, Paneth N, Saigal S.Psychopathology in young adults born at extremely low birth weight. Adolesc Med State Art Rev. 2013 Apr;24(1):330-40
Braun J, Weyhreter H, kohnlein O, storck M, Bode H : kognitives training: ein program zur forderung von vorschulkindern mit intellectual defitizen . psychologie in Erziehung und Unterricht ; 47 : 10-7
Brown RT, Wynne ME, Borden KA, Clingerman SR, Geniesse R, Spunt AL Methylphenidate and cognitive therapy in children with attention deficit disorder: a double-blind trial. J Dev Behav Pediatr. 1986 Jun;7(3):163-74
Butler RW, Copeland DR, Fairclough DL, Mulhern RK, Katz ER, Kazak AE, Noll RB, Patel SK, Sahler OJ. A multicenter randomized clinical trial of a cognitive remediation program for childhood survivors of a pediatric malignancy. J Consult Clin Psychol. 2008 Jun;76(3):367-78
Charollais A, Stumpf MH, De Quelen R, Rondeau S, Pasquet F, Marret S; Perinatal Network of Haute-Normandie Delayed language development at two years of age in very preterm infants in the Perinatal Network of Haute-Normandie. Early Hum Dev. 2014 Dec;90(12):891-2
Clark CA, Woodward LJ. Neonatal cerebral abnormalities and later verbal and visuospatial working memory abilities of children born very preterm. Dev Neuropsychol. 2010 2010;35(6):622-42
Cortese S, Ferrin M, Brandeis D, Buitelaar J, Daley D, Dittmann RW, Holtmann M, Santosh P, , Stevenson J, Stringaris A, Zuddas A, Sonuga-Barke EJ; European ADHD Guidelines Group (EAGG Cognitive training for attention-deficit/hyperactivity disorder: meta-analysis of clinical and neuropsychological outcomes from randomized controlled trials. J Am Acad Child Adolesc Psychiatry. 2015 Mar;54(3):164-74
Cowan N. Working Memory Underpins Cognitive Development, Learning, and Education..Educ Psychol Rev. 2014 Jun 1;26(2):197-223
Cowan N. Working Memory Underpins Cognitive Development, Learning, and Education. Educ Psychol Rev. 2014 Jun 1;26(2):197-223
Curtis WJ, Lindeke LL, Georgieff MK, Nelson CA Neurobehavioural functioning in neonatal intensive care unit graduates in late childhood and early adolescence. Curtis WJ, Lindeke LL, Georgieff MK, Nelson CA. Brain. 2002 Jul;125(Pt 7):1646-59
De Ribaupierre A, Bailleux C The development of working memory: further note on the comparability of two models of working memory. J Exp Child Psychol. 2000 Oct;77(2):110-27
Del Giudice E, Grossi D, Angelini R, Crisanti AF, Latte F, Fragassi NA, Trojano L.. Spatial cognition in children. I. Development of drawing-related (visuospatial and constructional) abilities in preschool and early school years. Brain Dev. 2000 Sep;22(6):362-7
Del Giudice E, Grossi D, Angelini R, Crisanti AF, Latte F, Fragassi NA, Trojano L.. Spatial cognition in children. I. Development of drawing-related (visuospatial and constructional) abilities in preschool and early school years. Brain Dev. 2000
Del Giudice E, Trojano L, Fragassi NA, Posteraro S, Crisanti AF, Tanzarella P, Marino A, Grossi D Spatial cognition in children. II. Visuospatial and constructional skills in developmental reading disability..Brain Dev. 2000 Sep;22(6):368-72

Diamond A.Executive functions. Annu Rev Psychol. 2013;64:135-68
Doyle LW, Anderson PJ, Battin M, Bowen JR, Brown N, Callanan C, Campbell C, Chandler S, Cheong J, Darlow B, Davis PG, DePaoli T, French N, McPhee A, Morris S, O'Callaghan M, Rieger I, Roberts G, Spittle AJ, Wolke D, Woodward LJLong term follow up of high risk children: who, why and how?. BMC Pediatr. 2014 Nov 17;14:279
Elvrum AG, Andersen GL, Himmelmann K, Beckung E, Öhrvall AM, Lydersen S, Vik T. Bimanual Fine Motor Function (BFMF) Classification in Children with Cerebral Palsy: Aspects of Construct and Content Validity. Phys Occup Ther Pediatr. 2015 Aug 19:1-16
Espy KA, Bull R, Martin J, Stroup W.Measuring the development of executive control with the shape school. Psychol Assess. 2006 Dec;18(4):373-81
Espy KA, McDiarmid MM, Cwik MF, Stalets MM, Hamby A, Senn TE. The contribution of executive functions to emergent mathematic skills in preschool children. Dev Neuropsychol. 2004;26(1):465-86
Galbiati S, Recla M, Pastore V, Liscio M, Bardoni A, Castelli E, Strazzer S.Attention remediation following traumatic brain injury in childhood and adolescence. Neuropsychology. 2009 Jan;23(1):40-9
Gathercole SE, Alloway TP, Willis C, Adams AM Working memory in children with reading disabilities.J Exp Child Psychol. 2006 Mar;93(3):265-81
Gathercole SE, Pickering SJ, Ambridge B, Wearing H The structure of working memory from 4 to 15 years of age.. Dev Psychol. 2004 Mar;40(2):177-90
Gathercole SE, Pickering SJ.Working memory deficits in children with low achievements in the national curriculum at 7 years of age. Br J Educ Psychol. 2000 Jun;70 (Pt 2):177-94
Goodman R, S Scott. Comparing the Strengths and Difficulties Questionnaire and the Child Behavior Checklist: is small beautiful? J Abnorm Child Psychol 1999; 27(1):17-24
Goodman R. The extended version of the Strengths and Difficulties Questionnaire as a guide to child psychiatric caseness and consequent burden. J Child Psychol Psychiatry 1999; 40(5):791-9
Goodman R. The Strengths and Difficulties Questionnaire: a research note. J Child Psychol Psychiatry 1997; 38(5):581-6
Grunau RE, Whitfield MF, Davis C Pattern of learning disabilities in children with extremely low birth weight and broadly average intelligence. Arch Pediatr Adolesc Med. 2002 Jun;156(6):615-20
Grunewaldt KH, Løhaugen GC, Austeng D, Brubakk AM, Skranes J. Working memory training improves cognitive function in VLBW preschoolers. Pediatrics. 2013 Mar;131(3):e747-54
Grunewaldt KH, Skranes J, Brubakk AM, Lähaugen GC Computerized working memory training has positive long-term effect in very low birthweight preschool children. Dev Med Child Neurol. 2015 Jul 3
Guimard P, Cosnefroy O, Florin A. Teacher rating of behaviors and competences, and prediction of academic performances and of later shool pathways in primary and secondary school. L ‘orientation scolaire et professionelle 2007 ; 36:179-202.
Halperin JM, Marks DJ, Bedard AC, Chacko A, Curchack JT, Yoon CA, Healey DM. Training executive, attention, and motor skills: a proof-of-concept study in preschool children With ADHD. J Atten Disord. 2013 Nov;17(8):711-21
Hutchinson EA[1], De Luca CR, Doyle LW, Roberts G, Anderson PJ; Victorian Infant Collaborative Study Group .School-age outcomes of extremely preterm or extremely low birth weight children. Pediatrics 2013 : 131 : E1053-61
Jarjour IT. Neurodevelopmental outcome after extreme prematurity: a review of the literature. Pediatr Neurol. 2015 Feb;52(2):143-52
Johnson S, Wolke D, Hennessy E, Marlow N .Educational outcomes in extremely preterm children: neuropsychological correlates and predictors of attainment..Dev Neuropsychol. 2011;36(1):74-95
Johnson S. Cognitive and behavioural outcomes following very preterm birth. Semin fetal neonat med 2007;12:363-73
Kail R. the development of memory in children . troisième edition. NY: wh Freeman 1990
Karch D, Albers L, Renner G, Lichtenauer N, von Kries R The efficacy of cognitive training programs in children and adolescents: a meta-analysis. Dtsch Arztebl Int. 2013 Sep;110(39):643-52

Kerr-Wilson CO, Mackay DF, Smith GC, Pell JP. Meta-analysis of the association between preterm delivery and intelligence. J Public Health (Oxf). 2012 Jun;34(2):209-16
Kibby MY, Marks W, Morgan S, Long CJ. Specific impairment in developmental reading disabilities: a working memory approach.. J Learn Disabil. 2004
Kirk HE, Gray K, Riby DM, Cornish KM Cognitive training as a resolution for early executive function difficulties in children with intellectual disabilities. Res Dev Disabil. 2015 Mar;38:145-60
Klingberg T, Forssberg H, Westerberg H Increased brain activity in frontal and parietal cortex underlies the development of visuospatial working memory capacity during childhood..J Cogn Neurosci. 2002 Jan 1;14(1):1-10
Korkman M, Kemp SL, Kirk U Effects of age on neurocognitive measures of children ages 5 to 12: a cross-sectional study on 800 children from the United States. Dev Neuropsychol. 2001;20(1):331-54
Kozulin A, Lebeer J, Madella-Noja A, Gonzalez F, Jeffrey I, Rosenthal N, Koslowsky M.Cognitive modifiability of children with developmental disabilities: a multicentre study using Feuerstein's Instrumental Enrichment--Basic program. Res Dev Disabil. 2010 Mar-Apr;31(2):551-9
Larroque B, Ancel PY, Marret S, Marchand L, André M, Arnaud C, Pierrat V, Rozé JC, Messer J, Thiriez G, Burguet A, Picaud JC, Bréart G, Kaminski M; EPIPAGE Study group.. Neurodevelopmental disabilities and special care of 5-year-old children born before 33 weeks of gestation (the EPIPAGE study): a longitudinal cohort study. Lancet. 2008 Mar 8;371(9615):813-20
Litt J, taylor Hg, klein N, hack M. Learning disability in children with very low birth weight : prevalence,neuropsychological correlates and educational interventions. J learn disabil 2005; 158: 15-20
Logie RH. The seven ages of working memeory. In : richarson JTE; Engle, RW; hasher, L ; Logie Rh Stoltzfus Er, Zacks, Rt editors . wokring memeory and human cognition. New York oxford university press; 1996.p 31-65
Løhaugen GC, Antonsen I, Håberg A, Gramstad A, Vik T, Brubakk AM, Skranes J.Computerized working memory training improves function in adolescents born at extremely low birth weight. J Pediatr. 2011 Apr;158(4):555-561
Marlow N, Wolke D, Bracewell MA, Samara M; EPICure Study Group. Neurologic and developmental disability at six years of age after extremely preterm birth..N Engl J Med. 2005 Jan 6;352(1):9-19
Marois P, Marois M, Pouliot Laforte A, Vanasse M, Lambert J, Ballaz L. Gross Motor Function Measure Evolution Ratio: Use as a control for natural progression in cerebral palsy. Arch Phys Med Rehabil. 2015 Aug 17
Marret S, Ancel PY, Marchand L, Charollais A, Larroque B, Thiriez G, Alberge C, Pierrat V, Rozé JC, Fresson J, Bréart G, Kaminski M; groupe EPIPAGE [Special outpatient services at 5 and 8 years in very-preterm children in the EPIPAGE study]. Arch Pediatr. 2009 Sep;16 Suppl 1:S17-27
Marret S, Chollat C, de Quelen R, Pinto Cardoso G, Abily-Donval L, Chadie A, Torre S, Vanhulle C, Mellier D, Charollais A, Ancel PY; Réseau de périnatalité en Haute-Normandie. [Course and neurological/behavioral development of preterm children]. Arch Pediatr. 2015 Feb;22(2):195-202
Mathiasen R, Hansen BM, Nybo Anderson AM, Greisen G Socio-economic achievements of individuals born very preterm at the age of 27 to 29 years: a nationwide cohort study..Dev Med Child Neurol. 2009 Nov;51(11):901-8
Melby-Lervåg M, Hulme C Is working memory training effective? A meta-analytic review..Dev Psychol. 2013 Feb;49(2):270-91
Mulder H, Pitchford NJ, Hagger MS, Marlow N.Development of executive function and attention in preterm children: a systematic review. Dev Neuropsychol. 2009;34(4):393-421
Mulder H, Pitchford NJ, Marlow N. Inattentive behaviour is associated with poor working memory and slow processing speed in very pre-term children in middle childhood. Br J Educ Psychol. 2011 Mar;81(Pt 1):147-60
Mulder H, Pitchford NJ, Marlow N. Processing speed and working memory underlie academic attainment in very preterm children. Arch Dis Child Fetal Neonatal Ed. 2010

Mulder H, Pitchford NJ, Marlow N. Processing speed mediates executive function difficulties in very preterm children in middle childhood. J Int Neuropsychol Soc. 2011 May;17(3):445-54
Nix RL, Bierman KL, Domitrovich CE, Gill S. Promoting Children's Social-Emotional Skills in Preschool Can Enhance Academic and Behavioral Functioning in Kindergarten: Findings from Head Start REDI.. Early Educ Dev. 2013;24(7)
Nosarti C, Giouroukou E, Micali N, Rifkin L, Morris RG, Murray RM. Impaired executive functioning in young adults born very preterm. J Int Neuropsychol Soc. 2007 Jul;13(4):571-81
Omizzolo C, Scratch SE, Stargatt R, Kidokoro H, Thompson DK, Lee KJ, Cheong J, Neil J, Inder TE, Doyle LW, Anderson PJ. Neonatal brain abnormalities and memory and learning outcomes at 7 years in children born very preterm. Memory. 2014;22(6):605-15
Pascoe L, Roberts G, Doyle LW, Lee KJ, Thompson DK, Seal ML, Josev EK, Nosarti C, Gathercole S, Anderson PJ. Preventing academic difficulties in preterm children: a randomised controlled trial of an adaptive working memory training intervention - IMPRINT study. BMC Pediatr. 2013 Sep 16;13:144
Pickering SJ Development of memory for pattern and path: further evidence for the fractionation of visuo-spatial memory. Gathercole SE, Hall M, Lloyd SA.. Q J Exp Psychol A. 2001 May;54(2):397-420
Pickering SJ. The development of visuo-spatial working memory. Memory. 2001 Jul;9(4):423-432
Rabiner DL, Murray DW, Skinner AT, Malone PS. A randomized trial of two promising computer-based interventions for students with attention difficulties. J Abnorm Child Psychol. 2010 Jan;38(1):131-42
Rai E. Long-term outcomes of prematurity in adolescence. Psychol Med. 2011 Aug;41(8):1763-74
Rapport MD, Orban SA, Kofler MJ, Friedman LM. Do programs designed to train working memory, other executive functions, and attention benefit children with ADHD? A meta-analytic review of cognitive, academic, and behavioral outcomes. Clin Psychol Rev. 2013
Ravens-Sieberer U, Auquier P, Erhart M, Gosch A, Rajmil L, Bruil J, Power M, Duer W, Cloetta B, Czemy L, Mazur J, Czimbalmos A, Tountas Y, Hagquist C, Kilroe J; European KIDSCREEN Group. The KIDSCREEN-27 quality of life measure for children and adolescents: psychometric results from a cross-cultural survey in 13 European countries. Qual Life Res. 2007 Oct;16(8):1347-56
Reidy N, Morgan A, Thompson DK, Inder TE, Doyle LW, Anderson PJ. Impaired language abilities and white matter abnormalities in children born very preterm and/or very low birth weight. J Pediatr. 2013 Apr;162(4):719-24
Robin J, Parkinson J. The potential for school based interventions that target executive function to improve academic achievement : a review. Review of educational research. À paraitre 2015
Rodrigues A, Befi-Lopes DM..Phonological working memory and its relationship with language development in children. Pro Fono. 2009 Jan-Mar;21(1):63-8
Rose SA, Feldman JF, Jankowski JJ, Van Rossem R Basic Information Processing Abilities at 11 years Account for Deficits in IQ Associated with Preterm Birth. Intelligence. 2011 Jul;39(4):198-209

Rose SA, Feldman JF, Jankowski JJ, Van Rossem R The structure of memory in infants and toddlers: an SEM study with full-terms and preterms.. Dev Sci. 2011 Jan;14(1):83-91
Rueda MR, Rothbart MK, McCandliss BD, Saccomanno L, Posner MI. Training, maturation, and genetic influences on the development of executive attention
Rushe TM, Rifkin L, Stewart AL, Townsend JP, Roth SC, Wyatt JS, Murray RM. Neuropsychological outcome at adolescence of very preterm birth and its relation to brain structure. Dev Med Child Neurol. 2001 Apr;43(4):226-33
Saavalainen PM, Luoma L, Laukkanen E, M Bowler D, Määttä S, Kiviniemi V, Herrgård E. School performance of adolescents born preterm: neuropsychological and background correlates. Eur J Paediatr Neurol. 2008 May;12(3):246-52
Saigal S, Stoskopf B, Streiner D, Boyle M, Pinelli J, Paneth N, Goddeeris J Transition of extremely low-birth-weight infants from adolescence to young adulthood: comparison with normal birth-weight controls.JAMA. 2006 Feb 8;295(6):667-75

Schulz KF, Altman DG, Moher D. CONSORT 2010 statement: Updated guidelines for reporting parallel group randomised trials .J Pharmacol Pharmacother. 2010 Jul;1(2):100-7
Shinaver CS 3rd, Entwistle PC, Söderqvist S. Cogmed WM training: reviewing the reviews. Appl Neuropsychol Child. 2014;3(3):163-72
Söderqvist S, Nutley SB, Ottersen J, Grill KM, Klingberg T. Computerized training of non-verbal reasoning and working memory in children with intellectual disability. Front Hum Neurosci. 2012. Oct 2;6:271
Spencer-Smith M, Klingberg T. Benefits of a working memory training program for inattention in daily life: a systematic review and meta-analysis.PLoS One. 2015 Mar 20;10(3)
St Clair-Thompson HL , stevens R, hunt A et bolder E. Improving children's working memory and classroom performance. Eductaionnal psychology 2010, 30, 203-219
Suchan B Neuroanatomical correlates of processing in visual and visuospatial working memory. Cogn Process. 2008 Mar;9(1):45-51
Swanson HL, Sachse-Lee C Mathematical problem solving and working memory in children with learning disabilities: both executive and phonological processes are important.J Exp Child Psychol. 2001 Jul;79(3):294-321
Swanson HL. Intellectual growth in children as afunction of domain specific and domain general working memeory subgroups. Intelligence , 2011. 39, 481-492
Swanson HL.Working memory and phonological processing as predictors of children's mathematical problem solving at different ages. Mem Cognit. 2004 Jun;32(4):648-61
Taylor HG, Minich NM, Klein N, Hack M Longitudinal outcomes of very low birth weight: neuropsychological findings. J Int Neuropsychol Soc. 2004
Taylor HG. Children born preterm or with very low birth weight can have both global and selective cognitive deficits. J Dev Behav Pediatr. 2006 Dec;27(6):485-6
Thorell LB , lindqvist S, Bergmen S, bohlin NG,, klingberg T. training and transfer of executive functions in preschool children developmental science 2009, 12, 106-113
Torchin H, Ancel PY, Jarreau PH, Goffinet F. [Epidemiology of preterm birth: Prevalence, recent trends, short- and long-term outcomes]. J Gynecol Obstet Biol Reprod (Paris). 2015 Jul 1
Torrioli MG, Frisone MF, Bonvini L, Luciano R, Pasca MG, Lepori R, Tortorolo G, Guzzetta FPerceptual-motor, visual and cognitive ability in very low birthweight preschool children without neonatal ultrasound abnormalities. Brain Dev. 2000 May;22(3):163-8
Van de Weijer-Bergsma E, Wijnroks L, Jongmans MJ. Attention development in infants and preschool children born preterm: a review. Infant Behav Dev. 2008 Sep;31(3):333-51
Van Noort-van der Spek IL, Franken MC, Weisglas-Kuperus N .Language functions in preterm-born children: a systematic review and meta-analysis. Pediatrics. 2012 Apr;129(4):745-54
Watkins MW, Beaujean AA. Bifactor structure of the Wechsler Preschool and Primary Scale of Intelligence--Fourth Edition. Sch Psychol Q. 2014 Mar;29(1):52-63
Zwicker JG, Harris SR. Quality of life of formerly preterm and very low birth weight infants from preschool age to adulthood: a systematic review. Pediatrics. 2008 Feb;121(2)

XIII APPENDIX

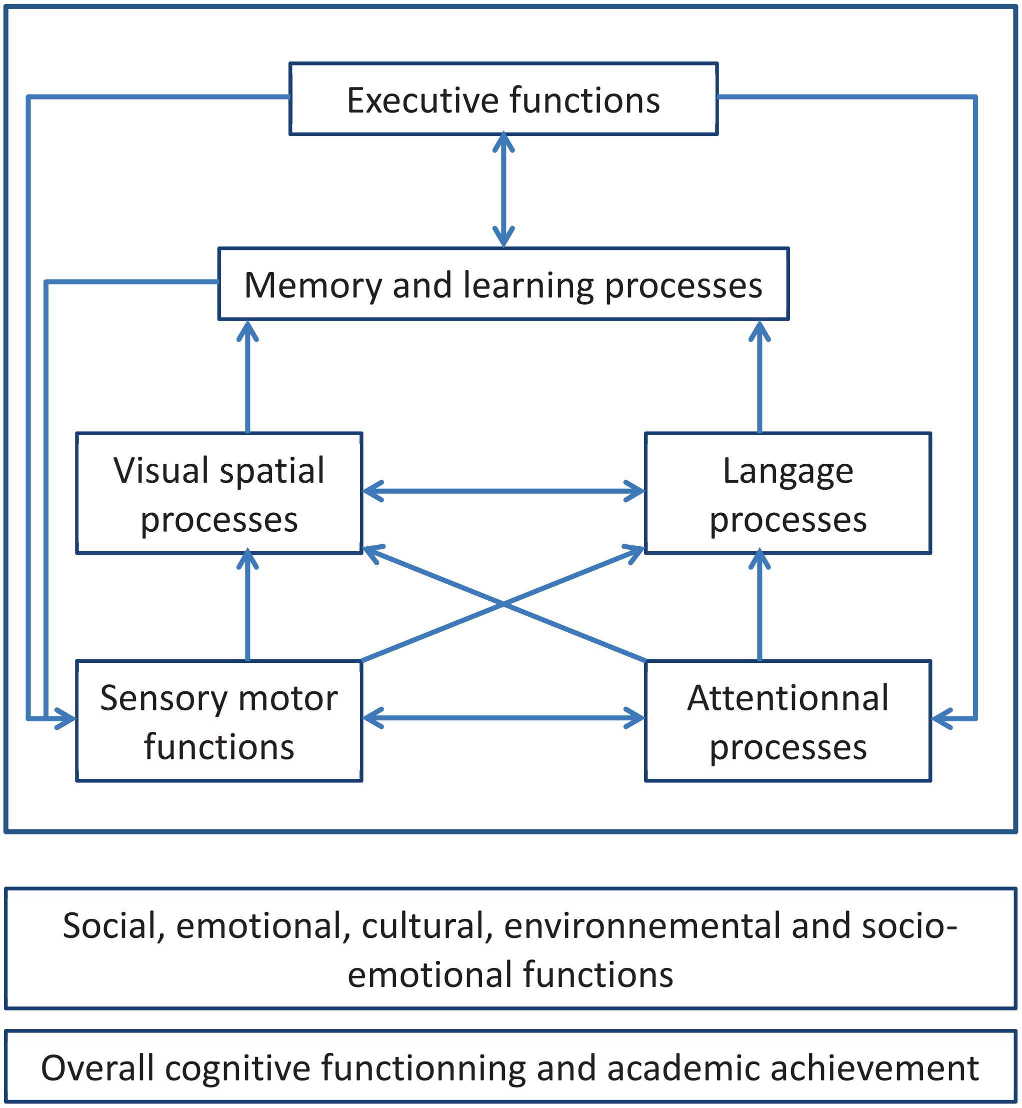

Appendix 1 – Conceptual model for scholl neuropsychological assessment

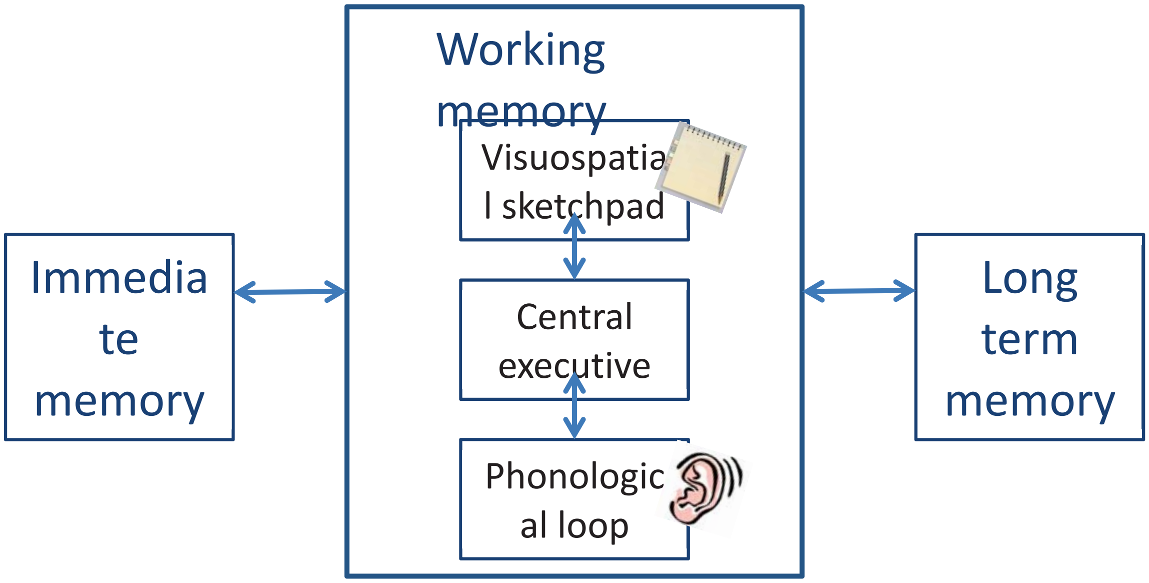

Appendix 2 – Working Memory model (Baddely and Hitch 1995)

Authors	Population	Participant characteristics	Preschool participants : Author, age years, CT, number	Diagnosis	Primary , secondary end points	Results
Kark D 2013	22 studies : children with various cognitive deficit	ADHD ,brain injury, specific learning disorder, healthy children, mental retardation, selective memory disorder, learning difficulties	Boivin MJ (6-16) (CT multi processes captain log) (RCT 32vs28)	Brain injury in connatal , hiv infection	Attention, EF,WM behavior	Attention, EF,WM behavior : NS
			Brown RT(5-13) (CT behavioral therapy) (RCT 19 vs14)	ADHD	Attention, EF,WM, behavior, School Attention, school , ,self esteem	***Wm,* FE*Behavior*,** School, attention, self esteem : NS
			Butler RW (6-17) (CT metacognition)(RCT 109vs54)	Brain injury (brain tumor)	Attention, EF,WM, behavior, School Attention, school , ,self esteem	***Behavior** , Attention : NS
			Kozulin A (4-20)(CT at school : figural visuomotor modality (99vs49)	Mental retardation	Intelligence	***Intelligence**
			Braun (5-7)(CT behavioral therapy)(CS 14vs14)	Brain injury	Attention, EF Intelligence	Attention: NS ***EF,*intelligence**
			Galbiati (6-18)(CT muliprocess: rehacom)(CS 40vs25)	ADHD	Attention EF behavior,Intelligence	***Behavior, *Attention**, ***Intelligence**, EF : NS
Melby – lervag 2013	23 studies adults and children with CT of WM)(4-72 ans)	Learning difficulties, WM deficit, ADHD, older adult, dyslexia,mental defiiciencies	**Bergman Nutley , moy 4.3 (4-5 ans)(RCT CT WM cogmed)** (101)	unselected preschool	Visuospatial WM, non verbal ability, problem solving, fluid intelligence	***WM visuo spatial** problem solving, fluid intelligence,: NS
			Saint clair thompson 2008, (moy 4.5) (RCT CT : memory booster)	unselected preschool	Verbal WM, visuo spatal WM, arithmetics	***Verbal WM, *Visuo spatial WM**, arithmetic : NS
			**Thorell 2009 (moy 4.8) (4-5 ans)RCT WM cogmed)(65)**	unselected preschool	Verbal Wm,visual WM, non verbal ability, attention : 2 points	***Verbal Wm, *visual WM*,** non verbal ability,: ns , attention : 0.05
Kirk 2015	17 studies (children with WM CT , 10 with attention CT)	WM in TD children and ADHD. Attention training in TD and ADHD, CT in children with ID	**Bergman Nutley , moy 4.3 (4-5 ans)(RCT CT WM cogmed) (101)**			
			**Thorell 2009 (moy 4.8) (4-5 ans)RCT WM cogmed)(65)**			
			**Soderq vist 2012 (6-12) (RCT CT WM: cogmed)(41)**	ADHD	Verbal WM,visual WM, short term memory, reaonning, parents ADHD symptom	***Verbal Wm,*visual WM, *short term memory ,** reasonning parents adhd symptom : NS
			Halperin 2012(5 ans) (CT teams : attention)(29)	ADHD	Parent and teacher rating attention,ADHD impairment	Parent and teacher rating attention*, ADHD impairment : NS
			Rudea(5 ans) (CT :attention)(37)	Unseslected preschool	FE	***Fluid intelligence**,vocabulary : NS
Megan spencer smith 2015	CT WM In inattention	ADHD, WM impairment	Grunewald 2013 5-6 ans (CS WM CT: cogmed**)**	WM impairement (VLBW)	Inattentive attention, verbal et visuo-spatial WM	*** attention, *verbal et visuo-spatial WM**

TABLE 1 – CT in preschool children in the different metanalysis (Kark 2013, Melby Lervag 2013, Kirk 2015, Magan Spencer –Smith 2015)

****significance: p<0.05, NS : non significant, gras : preschool children studies.RCT: randomized controlled studies , CS: controlled studies, WM: working memory, EF: executive functions, TD: typically developing, ADHD : Attention Deficit Hyperactivity Disorder,VLBW: very low birth weight*

Protocol and subsequent amendments for EPIREMED study

Protocol Version	Protocol Date	Summary of changes
Version 2	29/03/2016	The first version validated by the French Ethics Committee (*Comité de Protection des Personnes Sud Méditerranée V*) on April 12, 2016 and by the French National Agency for Medicines and Health Products Safety (*Agence Nationale de Sécurité du Médicament et des Produits de Santé*) on April 18, 2016.
Version 3	17/10/2016	The changes concerned:<br>- Withdrawal from an investigating center: Reims University Hospital.<br>- Addition of an investigative center: The CAMSP Center for Early Medico-Social Action of Reims "Being well in Champagne".<br>- Addition of investigators (attached CV dated and signed):<br>o Dr. Meriem Zahed (CHU Marseille);<br>o Dr. Julie Oertel (CHU Nice);<br>o Dr. Sophie Rubio (CHU Lyon).<br><br>This subsequent amendment to protocol was accepted by the French Ethics Committee (*Comité de Protection des Personnes Sud Méditerranée V*) on May 12, 2016.
Version 4	26/10/2017	The changes concerned the modification of sample size. Indeed, the number of children lost to follow-up was larger than estimated. Therefore, the simple size was re-calculated using the new rate of lost to follow-up.<br><br>This subsequent amendment to protocol was accepted by the French Ethics Committee (*Comité de Protection des Personnes Sud Méditerranée V*) on December 7, 2017 and by the French National Agency for Medicines and Health Products Safety (*Agence Nationale de Sécurité du Médicament et des Produits de Santé*) on December 06, 2017.

Long-term effects of visual spatial working memory training program performed at preschool age in very preterm infants with visual spatial working memory deficit
A Randomized Controlled Trial
EPIREMED

Programme Hospitalier de Recherche clinique

Appel à projets national 2015

Coordinator

Dr Catherine Gire	Neonatology, CHU Marseille, hôpital Nord, AP-HM, Marseille

Co-investigators

Pr Arnaud Catherine	Toulouse France Département de Santé Publique, Université de Toulouse III UMR 1027 Inserm
Pr Bednarek Nathalie	Reims France CAMSP Reims
Pr Cambonie Gilles	Montpellier France CHRU Montpellier
Pr Claris Olivier	Lyon France Hospices Civils de Lyon
Pr Debillon Thierry	Grenoble France CHRU Grenoble
Dr Foix-Hélias Laurence	Paris France APHP - Trousseau
Dr Garcia Patricia	Marseille France APHM - Conception
Dr Granier Michelle	Paris France Centre Hospitalier Sud Francilien
Pr Guillois Bernard	Caen France CHRU Caen
Pr Hascoet jean Michel	Nancy France CHRU Nancy
Pr Kuhn Pierre	Strasbourg France CHRU Strasbourg
Dr Lecomte Benedicte	Clermont-Ferrand France CHRU Clermont Ferrand
Dr Oertel Julie	Nice France CHU NICE
Pr Mitanchez Delphine	Paris France APHP - Trousseau
Pr Marret Stephane	Rouen France CHRU Rouen
Pr Patural Hugues	Saint Etienne France CHU Saint-Etienne
Dr Rubio Sophie	Lyon France Hospices Civils de Lyon
Pr Roze Jean Christophe	Nantes France CHRU Nantes
Pr Saliba Elie	Tours France CHRU tours
Dr Souksi Médioni Isabelle	Montpellier CHU
Dr Tréluyer Jean-Marc	CIC Necker APHP
Dr Zahed Meriem	Marseille France APHM - Nord

Collaborateurs

Mrs Calderon Johanna	Paris France Inserm, Equipe EPOPé, Unité 1153 Unité 1153

Methodologists

Pr Pascal Auquier	Public Health, CHU Timone, AP-HM
Dr Pierre-Yves Ancel	Inserm u 1153. Equipe EPOPÉ
Dr Karine Baumstarck	Clinic Research Plateform, AP-HM
Ms Monique Kaminski	Inserm u 1153. Equipe EPOPÉ

**Project management**	Direction of research and clinical innovation (DRCI) – APHM
**Quality assurance**	Good clinical practices and vigilances platform – DRCI APHM
**Sponsor**	Assistance Publique – Hôpitaux de Marseille

Sommaire

I SUMMARY 5
II INTRODUCTION: BACKGROUND AND RATIONALE 11
2.1 Premature birth concerns on public health issues 11
2.2 Executive Functions and, in particular, Working Memory 12
2.3 Executive Functions and Working Memory in premature children 14
2.4 Cognitive Training 16
2.5 Working hypothesis 18
2.6 EPIREMED: a new randomized controlled trial nested in the ongoing French national cohort EPIPAGE 2. 19
III OBJECTIVES 21
3.1 PRIMARY OBJECTIVE 21
3.2 SECONDARY OBJECTIVES 21
IV METHODOLOGY 22
4.1 DESIGN 22
4.2 PARTNERS 22
4.3 STUDY POPULATION 24
4.4 THE TWO STRATEGIES 24
4.5 ENDPOINTS 26
V GENERAL FRAMEWORK 29
5.1 STEERING COMMITTEE 29
5.2 IDENTIFICATION OF ELIGIBLE CHILDREN 29
5.3 BASELINE ASSESSMENT AND INCLUSION 30
5.4 RANDOMIZATION 30
5.5 FOLLOW UP FOR THE TWO GROUPS 31
VI DATA MANAGEMENT AND STATISTICAL ANALYSIS 32
6.1 SAMPLE SIZE 32
6.2 STATISTICAL ASPECTS 33
VII ORIGINALITY AND INNOVATIVE ASPECTS 35
VIII EXPECTED FINDINGS AND INDIVIDUAL/COLLECTIVE POTENTIAL BENEFITS 36
IX CALENDAR 37
X PROJECT FAISABILITY 38
10.1 PARTICIPATION OF A RESEARCH NETWORK 38

10.2 OTHER ELEMENTS OF PROJECTS FEASIBILITY 38
10.3 TEAM EXPERIENCE IN THE FIELD 38
10.4 FINAL REPORT AND PUBLICATION 40
10.5 CASE REPORTS FORM 40
XI REGULATORY AND ETHICAL ASPECTS 41
11.1 APPROVALS 41
11.2 CONFIDENTIALY 41
11.3 OTHER REGULATORY ASPECTS 41
XIII APPENDIX 51
Appendix 1 – Conceptual model for scholl neuropsychological assessment 51
Appendix 2 – Working Memory model (Baddely and Hitch 1995) 52
TABLE 1 – CT in preschool children in the different metanalysis (Kark 2013, Melby Lervag 2013, Kirk 2015, Magan Spencer –Smith 2015) 53

I SUMMARY

**SHORT TITLE**	**EPIREMED**
**LONG TITLE**	Long-term effects of visual spatial working memory training program performed at preschool age in very preterm infants with visual spatial working memory deficit. A Randomized Controlled Trial : EPIREMED
**TYPE OF STUDY**	Prospective, Randomized, Controlled, multicentric trial nested in a population-based epidemiological survey
**DURATION**	36 months
**NUMBER OF CENTRES**	19 centers
**RATIONALE**	Over the last years in France, Very Preterm (VP) birth rates and survival rate have risen. However, the proportion VP survivors with severe deficits have remained stable. Neuropsychological disorders and behavioral deficits are the deficiencies most encountered and have serious consequences on scholarship, familial and social adaptation as well as an impact on future adult lives. Compared to peers born at term, VP children generally perform poorly in Executive Functions (EF) and in particular in Working Memory (WM).<br>WM is defined as a “brain system that provides temporary storage and manipulation of information necessary for (…) complex cognitive tasks”. WM is regulated by a central executive control system and two subordinate subsystems: the visuospatial sketchpad and the phonological loop. This process is considered as a prerequisite for other EF such as reasoning and planning and for predicting intelligence and academic success. In VP children, WM impairment is linked to learning disabilities and is reported to have a strong influence on language and visuospatial processing. Overall academic achievements are thus impaired and an intervention strategy to minimize prematurity’s long-term WM impact needs to be developed.<br>WM can be improved by adaptive training tasks that encourage individuals to work continuously on their personal WM capacity. In recent years, several Cognitive Training (CT) programs focused on improving WM capacity. These programs have succeeded in improving individual performance in some specific WM capacities, but not in everyday functions such as other EF dimensions, language and visuospatial processing. Therefore, the functional benefits of CT have become controversial. Two recent meta-analyses (2015) were particularly focused on the efficiency of computerized visuospatial WM CT programs: Cogmed. The impact on the trained WM is significant and seems to be sustained over time. Improving non-trained functions such as verbal WM, attention, and secondarily learning disorders, is possible, but has yet to be proven on larger numbers. Further randomized studies are required to test CT efficacy in preschool-age VP infants.
**ORIGINALITY AND INNOVATIVE ASPECTS**	The EPIPAGE 2, a current French national population-based prospective cohort of 4,200 VP, born in 2011, offers an interesting and unique setting

	for carrying out a randomized study : EPIREMED trial.<br>EPIREMED is the largest study to assess the efficacy of a visuospatial CT (Cogmed) in preschool-age preterm children. Visuospatial CT is a well-adapted method to reach WM in this age group whose WM is only visuospatial (subcomponent visuospatial sketchpad), taking advantage of the neuroplasticity period. The central executive control system and the phonological loop will develop in children later on. Furthermore about 30% of VP children have low performances on the visuospatial processing. The intention of the study is to assess CT ability to improve visuospatial processing and moreover to assess its impact on the global function and learning abilities of the brain. To date, three small studies have shown that CT (Cogmed) may lead to improvements in WM and in other untrained activities in preschool age VP infants. If conclusive, this study will open the way for developing a preventative strategy for cognitive disorders in VP infants.
**STUDY OBJECTIVES**	
**Main objective**	The main objective is to assess the long-term effects (18 months) of CT (Cogmed) on visuospatial processing in preschool-age VP infants with WM impairment. Visuospatial processing is a broad cognitive process encompassing many subcomponents such as attention, sensory-motor skills, EF and visuospatial WM.
**Secondary objectives**	To assess, after 6 months of the intervention program, the effects of CT on the following parameters :<br>1 Intellectual functioning : global IQ (Wechsler Pre-Primary Scale of Intelligence)<br>2 Cognitive processing : language and visuospatial processing, working memory, fluidity of intelligence, and speed processing by five primary indexes of the Wechsler Pre-Primary Scale of Intelligence<br>3 Executive functions : auditory attention, planification and inhibition<br>4 Language processing and its abilities : ability of cultural and cognitive verbal learning, phonological judgment and semantics, verbal processing speed, verbal WM, motor programming, visual attention, analogizing ability<br>5 Child behavior<br>6 Parental anxiety<br>7 Child quality of life<br>8 School performance
**ENDPOINT MEASURE**	
**Primary end point (linked to main objective)**	The visuospatial index (VSI) : primary index scores of the WPPSI IV (Wechsler Preschool and Primary Scale of Intelligence). This index enables the assessment of visuospatial processing. It consists of two subtests : block design and object assembly. The average score is 100 and the standard deviation (SD) is 15.
**Secondary end points (linked to secondary**	1 Intellectual functioning : Global IQ, WPPSI IV (Wechsler Pre-Primary Scale of Intelligence)

**objectives)**	2 Cognitive processing, i.e. five primary index scores : Verbal Comprehension [VC], Visuospatial [VS], Working Memory [WM], Fluid Reasoning [FR] and Processing Speed [PS]. At age 5 and ½, each child in the EPIPAGE 2 cohort is assessed with the WPPSI IV. The WM index will detect children eligible for inclusion in the EPIREMED trial<br>3 EF, i.e. three subtests from the developmental Neuropsychological assessment (NEPSY 2) : Design fluency, Auditory Attention and Response set, Statue.<br>4 Language processing and its abilities : Battery CLEA (*Communiquer, Lire et Ecrire pour Apprendre*) 2 to 15 years - Evaluation of linguistic development assessment.<br>5 Behavior assessment by Goodman's Strengths and Difficulties Questionnaire (SDQ).<br>6 Parental anxiety: Spielberger State Trait Anxiety Inventory (STAI).<br>7 Quality of life : wellvalidated standardized French questionnaire assessed by parents (*Vécu et Santé Perçue de l'Adolescent parents*), VSPAp<br>8 School performance: Global School Adaptation questionnaire (GSA).
**STUDY POPULATION**	
**Main inclusion criteria**	1 Infants already included in the EPIPAGE 2 cohort, with a GA between 24 and 34 completed weeks, and with parents' agreement to participate in the follow up<br>2 Assessed with the WPPSI IV tool at age 5 ½ years follow-up<br>3 With a global IQ >70 at assessment with the WPPSI IV<br>4 With WM impairment, ie. a WM index <85 with the WPPSI IV<br>5. Parental consent for their child to participate in this study, coverage by medical insurance, and signed informed consent form
**Main exclusion criteria**	1 Severe cerebral palsy (CP) with a GMFCS score >2 and/or BFMF >2. The severity is based on the Gross Motor Function (GMFCS) and Bimanual Fine Motor Function (BFMF) classification system<br>2 Blindness or amblyopia, defined by a visual acuity <3<br>4 Deafness, as defined by a prescribed hearing aid<br>5 Chromosomal disorder or autistic syndrome<br>6 Children already included in the EPILANG study (another project linked to Epipage 2) or other interventional study<br>7 Children who do not speak French<br>8 Triplets
**EXPERIMENTAL DESIGN**	
	Open Randomized Clinical Trial**.** EPIREMED is a randomized controlled study comparing two strategies: CT versus standard care management.
**General framework**	1 Patient selection at the EPIPAGE 2 cohort follow-up using WPPSI IV (total IQ >70 and WM <85)<br>2 Inclusion and complementary neuropsychological assessment of the

	patients, consisting of the EF (NEPSY 2), language and its abilities (CLEA), infant behavior (SDQ), parental anxiety (STAI), quality of life assessment (VSPAp), School performance (GSA)<br>3 A computer-generated randomized list will be drawn up using a permuted block design (stratified on center and gemellarity, singleton/twin)<br>4 Cogmed program for subjects in the experimental arm and customary care management for subjects in the control arm,<br>5 Follow-up for both groups at 6 and 18 months after inclusion
**Experimental group (Cogmed)**	A "Cogmed" physician trained in computerized CT Cogmed, i.e., a neuropsychologist or speech therapist, independent of the initial assessment, will provide the parents with a document explaining WM and the software. Cogmed JM (4-7 years) is an online computer program for WM rehabilitation. This "Cogmed" physician will schedule and design the structure of the sessions. Depending on where parents can attend and have computer-internet access, sessions will be conducted at home or potentially with a "tutor" at a hospital or reeducation center. The child, accompanied by an adult, completes a series of interactive activities automatically adapted to each individual. After each session, the "Cogmed" physician will check the compliance and results online. The program calculates a performance score, representing the difference between the maximum and starting levels and offering analysis of the subject's progress compared to baseline. The parents are provided with 30-minute discussion every week. The program consists of three 15-minute sessions per week for 8 weeks.
**Control group**	The rehabilitation program will not be conducted in this group. The children will be followed up with customary care management. Speech therapy and/or academic support are recommended for those experiencing academic difficulties.
**Study schedule**	**We have planned :**<br>**1 For the experimental group :**<br>1.1 For the children : neuropsychological assessment at inclusion and two post-intervention assessments (8 and 18 months after the inclusion), and 8 weeks of CT,<br>1.2 For the parents : program management, regular phone calls to check up<br>1.3 For the teacher and parents : completing questionnaires at inclusion and 8 months after.<br>**2 For the control group :** The children will all undergo the same supplementary tests and the parents and teacher will receive the same questionnaires
**STATISTICAL ANALYSIS**	The sample size was calculated to obtain a 80% power to detect a difference of 7.5 points on the VS index (estimated standard deviation : 15)

	at 18 months between the 2 groups. This has been considered to be clinically significant. With the threshold for statistical significance set at a p-value of 0.05, assuming that a potential 15% of patients will be lost to follow-up, these calculations showed that 144 patients are needed (72 per group). A total of 166 individuals will be included, 83 for the control group and 83 for the experimental group. Assuming that 30-40% of the EPIPAGE 2 infants will present a WM abnormality, i.e. 1,600 infants, the recruitment will be ensured.
**NUMBER OF PARTICIPANTS**	**166**
**FEASIBILITY OF THE PROJECT**	**Participation of a research network.** EPIREMED operates within the EPIPAGE 2 cohort, 4,200 children, with follow-up rates of 87%. EPIPAGE 2, is already associated with nine complementary French projects, as well as the European EPICE and the French national cohort ELFE**.**<br>**Other aspects to insure the feasibility of the project.** The EPIREMED centers are organized for the diagnosis of WM deficits. The entertaining aspect of computerized CT (Cogmed) is adapted to the child's age. Cogmed has previously been used in contexts other than prematurity and tested in randomized studies: three of these, for preschool-age children, showed short term efficacy. The involvement of parents that are aware of their children's development helps assure follow up compliance.
**EXPECTED PATIENT OR PUBLIC HEALTH BENEFITS**	This project's primary goal is to demonstrate the necessity of early visuospatial VM assessment within the vulnerable population of VP children, and to prove the feasibility and efficacy of computerized CT using online software programs. Neurodevelopmental problems are common in VP children. Recent publications have reported many disorders in their specific cognitive functions, one of which is WM. Although neuro-development of VP children remains a public health priority because of their increased birth and survival rates, the interventions to improve this issue remain very few. There are currently no truly effective interventions to deal with academic achievement in preschool-age very preterm infants and their neuropsychological problems; thus, currently no specific management care is recommended for these children. Cogmed is based on interactive software and parental support. The program takes into account the child's environment, a factor of great relevance since it is closely linked to the child's future development. The expected benefits of CT (Cogmed) for those children in this study are enhanced WM, possibly leading to better EF by taking advantage of cerebral plasticity. A better global neuropsychological development improvement (language and visuospatial processing) can be expected with an improvement in learning and decreased behavioral problems. For parents, their guidance is required in Cogmed and can reduce their anxiety by fully embracing their role as primary agents in their child's development. This is consistent with recommendations for family-centered healthcare and can significantly

	improve the quality of life in both the children and their parents. In the long term, these improvements might also reduce those global costs linked to the consequences of extreme prematurity. Finally, if proved effective for this vulnerable population, this treatment can be a possible option or alternative in any other preschool population complaining of early academic difficulties related to WM deficits.

II INTRODUCTION: BACKGROUND AND RATIONALE

2.1 Premature birth concerns on public health issues

2.11 Becoming aware of premature birth issues

The EPIPAGE 2 study shows that the advances made in perinatal care have increased the premature survival rates as well as increased their census in the general population (Torchin H 2015, Ancel PY 2015). Each year there are about 10,000 very preterm births (births before 33 weeks of amenorrhea [SA]) in France. The consequent increase in this population's survival rate warrants an evaluation of their future (Ancel PY 2015, Jarjour IT 2015, Doyle LW 2014, Anderson PJ 2014). Severe sequelae such as cerebral palsy have declined, but specific cognitive difficulties, most often in multiple areas, (motor, language, visual-spatial, attention, executive function, etc.) and minor motor disorders are still very frequent. These sequelae can hinder normal scholastic development and social integration and require an increased need for rehabilitation during preschool and school ages (Marret S 2014).

In 1997 the EPIPAGE 1 cohort study included all very premature births in nine French regions in order to assess their neuro-developmental futures at ages 5 and 8 as compared to term births. In this study, 2357 surviving children born very preterm, 1817 (77%) were assessed at a median age of 5 years. 33% had their MPC (Mental Processing Composite) below 85 (i.e., -1 standard deviation (SD) below normal) and there were 12% scoring below 70 (- 2SD) (the threshold indicating cognitive impairment); as compared to 11% and 3% respectively of children born at term (Larroque B 2008). Half of the premature children received care that was mainly speech and psychological therapy (Marret S 2009).

In 1995 the EPICURE study in the United Kingdom and Ireland observed intellectual difficulties in children born extreme premature (before 26 SA). In this study, 308 surviving children, 241 (78%) were assessed at a median age of six years and four months. There were 46% with severe cognitive deficit (IQ <-2SD) and 30% with moderate cognitive impairment (between -1 and -2 SD) (Marlow N 2005).

Overall, there are cognitive deficits associated with prematurity that increase as the gestational age decreases: 1.5 IQ points per week from 33 SA (Johnson S 2007), regardless of improved perinatal care (Anderson PJ 2014, Bhutta AT 2002, Kerr Wilson CO 2012).

2.12 Learning disorders of very and extremely premature infants

It is shown that very premature babies have less success in school and in their professional life. In the EPIPAGE study, 5% of eight year old children born very prematurely were enrolled in institutional or specialized classes and 95% in regular classes. This is compared with 1% and 99% respectively of those children born at term. Among those children in regular classes, 18% of the very premature births repeated a class at least once compared to 5% of term births (Marret S 2009).

Scholastic failure increases with decreased gestational age. There are studies that segregate out mental impairments and neurosensory disorders and focus exclusively on the learning problems of premature children.

Grunau shows that 65% of 8-9 year old who are extremely prematurely born have learning disabilities compared with 13% of full-term children of the same age (Grunau RE 2002). A meta-

analysis confirms the performance difference in these two birth groups with a difference of 0.5 SD for reading, 0.8 SD for phonology and 0.6 SD in mathematics (Aarnoudse-Moens CS 2009).

In a study of school aged children born extremely premature, there is a performance difference of 0.5 to 0.6 SD in reading, mathematics and phonology compared to children born full-term (Hutchinson EA 2013). In the EPICURE study, 11-year old born extremely premature performed at -1 SD in reading and -1.7 SD in math compared to term infants. In the same study, 52% of the premature children were dyslexic vs 11 % of those born full term and respectively, 70% vs 14% had dyscalculia (Johnson S 2009).

Litt reports that school children aged 8-9 year old with less than 750 grams birth weight had seven times more dyslexia, six times more dyscalculia and 13 times more dyscalculia combined with dyslexia than full-term children (Litt J 2005).

These problems persist into adolescence and adulthood resulting in poor integration in high school and university (Saigal S 2006, Mathiasen R 2009, Rai E 2011) as well as increased unemployment, under qualified employment, anxiety disorders and a loss of self-esteem (Boyle MH 2013). Zwicker studied prematurity's impact on the child's and family quality of life (QOL) during the child's early, adolescent and young adult years. The impact appears more marked in preschoolers than in the adolescent and young adult (Zwicker JG 2008).

2.2 Executive Functions and, in particular, Working Memory

2.21 Definitions of Executive Functions (Appendix 1)

Executive Functions (EF) define the cognitive operations that allow an individual to adjust his behavior and activity in response to environmental requirements and fluctuations.

The EF comes into play when an individual is confronted with a "non-routine" situation that requires problem solving. The principal mental processes characterizing EF are:

- organizing and planning data based on what needs to be achieved, choosing relevant information,
- implementing processing operations, inventing new situations and modifying them if they deviate from the purpose (Anderson PJ 2012, Parkinson J 2015),
- suppressing extraneous information, resisting distractions,
- organizing task-relevant information in the memory to use them later (Working Memory : WM).

It is understood that each of these mental processes or "executive mental functions" can be evaluated by "specific" tests but they are often interlinked and dependent on the mental attention processes (auditory and/or visual). For this reason, definitions vary significantly from publication to publication (Epsy KA 2004, Parkinson J 2015).

Schematically we can isolate four main executive mental processes :

1. ***Planning*** : mental diagram of a single action, anticipating the goal to be reached - "how to achieve that goal"
2. ***Flexibility*** : adaptation of an action plan for environmental contingencies, ability to modify strategies in case of error, maintain attention - "the art of adapting to change"

3. ***Inhibition*** : capacity to ignore distractions and resist giving one reply rather than another
4. ***Working Memory (WM)*** : ability to store verbal or visuo-spatial information in one's mental space and to manipulate them, implementing strategies, processing action sequences, reasoning - “*art de faire*”.

All in all, the EF are the "superior" functions that play an important role in cognitive development and social adjustment (Diamond A 2013) (Appendix 1).

2.22 Working Memory: the organizational components (Appendix 2)

WM is a key determinant of several higher–order cognitive functions such as reasoning, fluid intelligence, problem solving and language comprehension (Borella E 2010).

According to Baddeley, the working memory (WM) is based on the coordinated functions of a set of autonomous sub-components (Baddeley A 1996, Shuchan B 2008, Cowan N 2014) :

1. ***The phonological loop or verbal WM***. The phonological loop is responsible for storing visually or aurally presented verbal information. It is composed of two sub-components: a phonological reserve and a recapitulating articulatory loop. For example, when auditory information appears, a phonological analysis is stored as phonological codes. Sentence comprehension, particularly sentences that are long and complex, is based on this mechanism. This subcomponent allows to transfer verbal information, which was obtained visually, to the phonological storage system. The phonological loop is only fully operational when a child is about 8 year old, although the first elements appear at age 4.
2. ***The visuo-spatial sketchpad or visuo-spatial WM***. Visuo-spatial information is encoded by the visuo-spatial sketchpad and is distinguished within its interior with a system for descriptive visual information (shapes, colors...) and a system for spatial information (location). Studies have shown that the motor WM is involved in morphogenetic movements (reproduction of a given body form) and topokenetics (movements made according to a goal and taking into account the environmental information). These are in close collaboration with the visuo-spatial Working Memory. The visuo-spatial storage system maintains information and its variations, interacting with a spatial attention system, thereby facilitating the retention of location information. This is used to resolve visual and spatial type tasks such as space orientation when using a map. The visuo-spatial sketchpad is also responsible for the ability to transform and rotate mental images. As a result, we can mentally describe and explore an object or place.
3. ***The Central Administrator***. It has the function of management and control, it also supervises and coordinates the phonological loop and the visuo-spatial sketchpad. It inhibits irrelevant information and activates information stored in long-term memory such as reasoning, decision-making and action planning. It enables the WM to change continuously its responses based on recent internal information (from the long-term memory) or external information (from sensory input). This updating process supervises the passage of information between subsystems and long-term memory.
4. ***The episodic buffer***. This is a temporary storage system for multi-modal information. As a "buffer” it functions as a temporary interface between the phonological loop and the visuo-spatial sketchpad as well as long-term memory. It is "episodic" because it stores "episodes" in which information is integrated in space and time. This buffer is different from episodic memory because it functions as a temporary storage system but can be preserved in

amnesic patients with major impairment of long-term memory. This storage system is also controlled by the central administrator.

2.23 Working Memory development in children

The performance of WM increases with age (Logie R 1996, Pickering MJ 2001). Initially, young children do not have the ability to encode information in phonological form, and the visual-spatial encoding of objects assumes that role (Suchan B 2008, Kail R 1990, Cowan N 2014). There seems to be predominance for spatial encoding up to the age of 8-10 (De Ribeaupierre A, 2000).

Depending on the WM sought, we observe variations of this function. We also note that boys have a visuo-spatial span mnemonic slightly higher than girls. Nonetheless, the rhythm of this span is not correlated with the intellectual development of the child and there are obviously large inter-individual variations.

Some memory functions of the intervening central administrator such as inhibition of irrelevant information, treatment strategies, execution speed, gradually develop during childhood (Pickering SJ, 2001).

The evolution of attentional capacity can therefore affect the child's ability to grasp information and to inhibit irrelevant information (Cowan N 2014). In this sense, the challenge of this developmental phase seems to be particularly focused on the linking between memory processes, executive functions and attention (Rose SA 2011).

2.24 Executive functions, Working Memory and learning difficulties

EF, WM and attention have related developmental and functional operations and are involved in the development of more complex cognitive processes such as language or visuo-spatial performance (Swanson HL 2011). Their alterations can therefore change intelligence as measured by IQ (J Parkinson 2015, Alloway TP 2010). Many studies have confirmed a correlation between EF and WM abnormalities and learning disabilities such as dyslexia or dyscalculia (Gathercole SE 2000, 2004, 2006, Swanson HL 2004, Espy KA 2004, 2006, Alloway TP 2010, Clark CA 2010). Robin and Parkinson confirm this correlation in a meta-analysis of 67 cross-sectional studies published over the last 25 years on school children of varying ages (Robin J and Parkinson J 2015).

Most studies have been published after 2010 and demonstrate an evolving interest in topic with 35% of these studies focusing only on WM. The link between abnormal EF and learning disabilities remains the same in each age group (3-6 years, 6-11 years and 12-18 years) regardless of the analyzed executive function (planning or inhibition or mental flexibility or WM) and the concerned learning disabilities (dyslexia or dyscalculia).

2.3 Executive Functions and Working Memory in premature children

2.31 Executive Functions and prematurity

EF in very preterm children has generated considerable interest, with numerous studies reporting this cognitive domain to be an area of concern for this population. There are two meta-analyses of EF in premature children.

From their studies published between 1990 and 2008, Mulder and al in 2009 analyze each executive process in a total of 830 very prematurely born school children vs 740 born full-term (Mulder H

2009). The authors show a difference of 0.3 SD for inhibition, 0.5 SD for verbal WM and 0.4 SD for the planning compared to the full-term control group. These differences are even greater for those with lesser gestational ages (within 26 SA: a difference of 0.5 SD for the inhibition and 0.7 SD for verbal WM). The gap is interesting, especially for verbal WM, and it increases with the age of children suggesting a worsening over time. Mental flexibility remains unchanged as compared to the control group.

The Aarnoudseen-Moens's review concerns children born between 1998 and 2008 comparing very extremely premature births to those full-term births (Aarnoudseen-Moens CSH 2009). Only two executive components are covered: WM and mental flexibility. This metaanalysis shows a decrease of 0.6 SD for verbal WM and 0.4 SD for the visuo-spatial WM. Differently from Mulder, this difference stabilizes with age, and mental flexibility is impacted 0.5 SD as compared with the control group.

2.32 Working Memory and prematurity

Almost all studies, regardless of the child's age, show a lower verbal and visuo-spatial WM performance in the very premature child compared to full-term children (Anderson PJ 2004 Aarnoudseen Moens CSH-2009, Rose Baron SA 2011).

In very premature born preschool children, visuo-spatial WM is not as good as those born at term (Epsy KA 2002, Borradori Tolsa C 2014, Baron IS 2010). This is confirmed for verbal WM skills for school age children and adolescents (Taylor HG 2000, 2004). In 2013 Omizollo studied the correlation between WM (verbal and visuo-spatial) and the learning of a 7 year old very prematurely born cohort. The very premature group had 2.1 to 3.5 times more deficit in WM than the control group (Omizollo C 2013).

There is a significant risk for dysexecutive disorder in preschool and school age children with very premature births. Despite this, there are very few long-term studies of the trajectory of these dysexecutive disorders and it is not yet clear whether these disorders are responsible for developmental delays (Anderson PJ 2015). In fact, studies of adolescents or adults are contradictory. Dysexecutive disorders reported in prematurely born adolescents and adults suggest these symptoms persist beyond school age (Taylor HG 2004, Novartis C 2007). However, the absence of a difference or a marginal difference of WM performance is found in other studies of adolescents (Rushe TM 2001, Saavalainen P 2007, Curtis WJ 2002).

2.33 Prematurity: diffuse or selective cognitive impairment ?

The extremely preterm birth population is vulnerable to a lower overall IQ than the general population and therefore requires more discriminative neuropsychological testing to identify affected functions (Anderson PJ 2014).

Hutchinson and Anderson speculate on the damage to a primary, original cognitive function (Hutchinson EA 2013 and Anderson PJ 2013, 2014). For example a WM deficit, and/or attention and/or processing speed, which impact other mental processes, could be the cause of delayed language or dysexecutive disorders (Mulder H 2010, 2011).

This is why early determination of specific cognitive profiles, beginning at a pre-school age, could allow more focused monitoring and/or development of interventional strategies (Taylor HG 2006).

Mulder and al were interested in the origins of learning and attention disorders as well as the EF disorders in the very premature vs term-birth children (Mulder H 2010, 2011). These authors report that the processing speed and WM are independent predictors of academic difficulties amongst the very premature-birth preschool children. According to these authors, the processing speed deficit (which is dependent on gestational age) is correlated to EF : WM (verbal and visuo-spatial), inhibition, cognitive flexibility (Mulder H 2010, 2011). Similarly there is a correlation between the measurement of the processing speed and/or WM and the symptoms of impulsivity/hyperactivity, attention disorders.

2.4 Cognitive Training

2.41 Definition of Cognitive Training

The identification of weak processes in some pathologies, as well as improvement in knowledge of brain function development and the possibility of neuronal plasticity, have led teams of researchers to develop intervention programs focusing on cognitive processes described as Cognitive Training (CT). In short, the CT is defined as the rehabilitation of one or several altered cognitive functions. CT is based on the concept that repeated practice within a specific domain will result in gain in both cognitive and behavioral efficiency of the targeted domain as well as subsequently transferring improvements to untrained domains.

Two types of techniques are used:

- Restorative (training of the defected function through repeated practice),
- Development of alternative skills (compensatory strategy).

The CT may be based on different types of materials: paper/pencil, card games, pieces of wood, behavioral therapies, and software. Specific computer techniques (software) have been developed; they continuously and automatically adjust to task levels and difficulties as well as to the subjects' capabilities in order to optimize the effects of training. This made the scientific community react with a sense of caution against this highly lucrative industry (Parkinson J 2015). Similarly the link between EF deficits and learning disabilities has revived the interest of CT techniques in schools so as to improve standards (Parkinson J 2015). For this reason, many researchers have raised the question of rehabilitation of EF in recent years and asked if CT of EF could improve learning in the general population ; in children with neurocognitive disorders ; and more specifically, in those with premature births.

2.42 Cognitive training in the general population: intervention in school

In 2015 Robin and Parkinson, by means of a meta-analysis of school children having no mental deficiencies, report only five randomized studies with control-groups analyzing interventional components (Robin J 2015, Parkinson J 2015). If the executive functioning is changed by intervening in schools, does that then lead to children's improved achievement? The authors note that there are few studies with any controls for such characteristics as parental education, socio-economic status or IQ, although these characteristics have been found to be associated with EF development. These show that the EF could be improved by CT but with little performance impact on reading and/or mathematics. There is only one study (Rabiner DL 2010), performed on school-aged children and using an attention re-education computer program, which improved attention spans and class

behavior but not directly improved achievement. Other criteria such as repeating a class or the student future are not analyzed.

Bierman and Torres describe the range of what are designed to influence EF in school age children and explore whether or not there is evidence that EF improve as a result (Bierman KL 2015, Torres M 2015). They conclude there is a mixed evidence for the malleability of EF as a result of interventions.

2.43 Cognitive Training in population with cognitive disorders (Table 1)

A meta-analysis (Kacrh D 2013) uses the CT studies of EF in children and adolescents in cases of cognitive impairment. The effects are generally low on EF and attention ([0.18 SD, 95% CI 0.11 to 0.47] and [0.17 SD, 95% CI 0.12-0.42]), with an unexceptional WM (0.65 SD, 95% CI -0.12 to 1.42). The most consistent benefit concerns the improvement of behavioral disorders (0.58 SD, 95% CI 0.31 to 0.85), but this result comes from studies with no control groups. Moreover, only two studies (57 patients) involved preschoolers when brain plasticity is the greatest and where there is a significant improvement in intelligence (IQ) and behavior (Galbiati S 2009, Braun J). Several CT meta-analyses of EF in children with attention deficit disorder and hyperactivity (ADHD) show an improvement in trained EF but the effects on ADHD remain moderate and sustained with slow improvement in time. (Rapport MD 2013, Cortese S 2015).

A CT meta-analysis of WM led by Melby-Lervag involving children and adults with cognitive disorders shows a beneficial CT effect on the visuo-spatial long term WM, without influencing other EF, verbal or non-verbal intelligence or learning (Melby-Lervag M 2013). Only three studies involve children less than 7 years (St ClairThompson HL 2010, Thorell L 2008, Bergman-Nutley SB 2011). One other meta-analysis confirms this author's results in children. (Kirk HE 2015).

The main limitation of these meta-analyses is the heterogeneity of the treated disorders: ADHD, TC sequelae, dysexecutive disorders, learning disorders, typically developping children,.etc., appearing in the various age groups as well as different intervention types, their heterogeneous duration, and the different impact indicators that were measured.

However these meta-analyses show in the population of preschool children (despite the limited number (less than 200)), a significant positive CT effect on the WM or other trained EF along with a possible transfer to other functions such as attention, behavior..., intelligence (Soderqvist S 2012, St Clair Thompson HL 2010, Bergman- Nutley SB 2011, Thorell L 2008).

All in all, the EF rehabilitation, and specifically the WM's impact on the various intelligence and learning processes, remains a current scientific debate.

While there are many rehabilitation systems developed, there is no "gold standard" regarding CT benefit in kind, duration and frequency. There exist some methodologically weak studies showing an improved EF with re-education but with little effectiveness on intelligence and learning performance, except in the preschool child.

A large scale, well conducted study on the impact of EF rehabilitation interventions in preschool age children is therefore indicated.

2.44 Cogmed JM : Cognitive Training of the visuo-spatial Working Memory

Cogmed JM is a technique for re-educating the visual spatial WM and is used in many randomized studies of CT, WM and FE (Shinaver CS 2014). It consists of computer software with a specific set of visuo-spatial VM tasks and adjustable levels of difficulty by using a precise algorithm.

Each day, the user performs a predetermined number of events. These results are retained during the training and can provide the basis for further analysis. (Torkel Klingberg, developed by Helena Westerberg and other researchers, Department of Neuropediatrics Astrid Lindgren Karolinska University Hospital and Jonas Beckeman and David Skoglund, video game developers Klingberg).

Two recent meta-analyses (2015) are particularly interested in the efficacy of Cogmed (Shinaver CS 2014, Spencer-Smith M 2015). The impact on the trained WM visual spatial is significant and seems to be sustained over time. Improving non-trained functions such as verbal WM, attention, and secondarily learning disorders, is possible, but has yet to be proven on larger numbers. Above all the WM CT studies of preschool age children were realized with Cogmed JM (Bergman Nutley SB 2011, Thorell LB 2009, and Sodervisqt S 2012).

2.45 Cognitive Training and prematurity

Three studies on premature infants, examined the effects of CT by Cogmed on visuo-spatial WM (Grunewald KH 2013, 2015, Lohaughen GC 2011). The first was a sample of 16 preterm adolescents vs. 19 teenagers with term births. Another was performed on 20 very premature preschool children aged 5-6 years. The third study included 20 very premature preschool children vs 17 in the control group (term birth).

There appears to be a beneficial effect on the trained WM visuo-spatial and a possible transfer to other processes (verbal working memory, attention, etc.). However, these three studies contain very small samples from very different age groups. In two studies the WM is in the process of maturation and in the third, it is practically consolidated. Although, monitoring was limited to seven months and learning disorders were not assessed, they represent preliminary studies with encouraging results.

2.5 Working hypothesis

In recent years the severe deficiencies of prematurity have been stabilized. The majority of sequelae appears as moderate neuropsychological disorders but with serious school, family and social repercussions. These disorders, which persist into adulthood, result in lower social integration. Very premature-birth children present with frequent WM deficits.

WM in these school age children might be correlated with subsequent learning disorders and the origin of complex deficits such as language delays or visuo-spatial performance disorders (Mulder H 2009). This WM vulnerability in the premature needs to be confirmed and might be an avenue for preventive interventions.
WM is a set of processes that maintains active information necessary for performing common cognitive activities, notably the ability to retain information sufficiently long enough so as to manipulate data in the future. WM is controlled by a central administrator module located in the prefrontal cortex that oversees two peripheral modules: the phonological loop and the visual sketchpad. This is linked to other EF such as reasoning, planning, intelligence and academic learning

and participates in multidimensional skills such as language and visuospatial processes (Alloway TP 2006).

A current research question is about the link between EF rehabilitation, including WM, and its impact on intelligence and learning. Cognitive Training (CT) is an interesting approach to the management of WM disorders. While many types of CT interventions improve WM, the benefits on EF, language, visuo-spatial performance and intelligence remain controversial (Parkinson J 2015, Kirk HE 2015, Melby-Lervag M 2013). To date, three small studies have shown that CT (Cogmed) may lead to improvements in WM and in other untrained activities in preschool age VP infants.

Many re-education techniques (books, games, software, etc.) have emerged in recent years without any "gold standard". Cogmed JM software seems efficient among preschool children in recent meta-analyses (Shinaver CS 2015, Melby-Lervag M 2013, Kirk HE 2015) and is also effective in three preliminary studies of preterm infants.
The strategy is to take advantage of brain neuroplasticity by measuring the consequences of visual spatial WM restoration on the overall brain function and learning in very premature preschool children (Grunewald KH 2013, 2015). The hypothesis is that doing CT with preschool children born very preterm (just as their WM is emerging and uniquely non-verbal: visual-spatial sketchpad), may decrease the subsequent dysexecutive disorders, improve intellectual performance (both visuo-spatial and language processing) as well as school integration.

2.6 EPIREMED: a new randomized controlled trial nested in the ongoing French national cohort EPIPAGE 2.

The EPIPAGE 2 is an ongoing national prospective cohort of 4,200 very preterm children born in France in 2011 and offers an interesting and unique setting for implementing a randomized trial: EPIREMED study.

EPIPAGE 2 study aims to examine short- and long-term outcomes of very preterm children and their determining factors. This project seeks to provide new data on the prognosis and etiology of very preterm birth and to assess related medical practices. Accordingly, it should lead to the development of new strategies of management and prevention in high-risk babies. Eligible participants for this prospective population-based study included all live or stillborn infants and all pregnancies terminated between 22 and 31 weeks of gestation occurring in all the maternity units in 25 French regions. In addition, a sample of moderate preterm births, i.e. births and late terminations at 32-34 weeks, was included from the same regions. In all, 7,804 babies (stillbirths and live births) and terminations of pregnancy out of 8,400 eligible births in France in 2011 that were either very preterm (22-31 weeks) or moderately preterm (32-34 weeks) were included. Data on pregnancy, delivery, and neonatal events were extracted from the obstetric and neonatal records. Of the 4,467 children discharged alive from the hospital and eligible for follow-up, 155 (4%) of the families refused further follow-up and 22 died before one-year of age. Finally, 4,290 were included in the follow-up. Nine additional projects are collecting specific data, in addition to the core cohort data, to investigate specific hypotheses among subsamples of the cohort in order to investigate 1) diagnosis of histologic chorioamnionitis, 2) early biomarkers of the child’s health, 3) attitudes of care for extremely preterm infants, 4) painful procedures in neonatal intensive care units, 5) neonatal MRI cerebral abnormalities and their relation to executive functions, 6) associations between early gut

colonization and early and late disease onset, 7) impact of neonatal nutrition on the child's development, 8) mother-infant attachment 9) a randomised, controlled trial to evaluate if a parent-implemented intervention before the corrected age of 3 years in very preterm children with a language delay can improve language.

The follow-up has collected information at corrected ages of 1 (2012) and 2 (2013) years of age and the next steps will be at 5 ½ (2016), 8 (2019), and 12 (2023) years of age.

EPIREMED, the tenth additional project, will be randomised, controlled trial nested in the EPIPAGE 2 cohort. This trial will assess the effectiveness of CT programs on preterm infants of preschool age (5 ½ year old) that have a visuospatial WM deficit. Visuospatial CT is a well-adapted method to reach WM in this age group whose WM is only visuospatial (subcomponent visuospatial sketchpad), thus taking advantage of the neuroplasticity period.

III OBJECTIVES

3.1 PRIMARY OBJECTIVE

The primary objective is to assess the long-term effects (18-months post inclusion) of Cognitive Training (Cogmed JM) on the visual-spatial processing in pre-term birth preschool-aged children with Working Memory impairment.

Visual-spatial processing is a broad cognitive process encompassing many subcomponents such as attention, sensory-motor, executive function and visuo-spatial working memory. The visuo-spatial index is the primary endpoint.

3.2 SECONDARY OBJECTIVES

The secondary objectives are to assess at the six month post-intervention (8 months post inclusion) the effects of the cognitive training on the following parameters:

- children' parameters :
  - Global intellectual functioning,
  - Different cognitive processes : language, visual-spatial processing, speed processing, working memory, and fluidity of intelligence,
  - Other composites of Executive Functions : auditory attention, flexibility, and inhibition,
  - Language processing abilities : verbal learning abilities (cultural and cognitive), phonological judgment and semantics, verbal processing speed, verbal WM, motor programming, visual attention, and ability to analogize,
  - Behavior and quality of life (QOL),
  - School performance.

- Parents' parameters :
  - Anxiety level.

IV METHODOLOGY

4.1 DESIGN

This trial is a multicenter, randomized, controlled, open-label, two-parallel group study. The recruitment will be prospective.

The two groups are :

- A control group : standard care management
- An experimental group : standard care management in association with a 2-month Cognitive Training program called Cogmed JM

Study design rational:

A Randomized Controlled Trial is the experimental design providing the highest level of evidence with regard to the existing literature in this field. The open design is the only possible option due to the nature of the experimentation. We assume that the primary endpoint will be collected in a standardized procedure ensuring an objective measure. Both the healthcare worker in charge of the evaluation of the primary endpoint and the statistical analysis will be blinded.

4.2 PARTNERS

The multicenter randomized controlled trial, EPIREMED study will use the design of the existing EPIPAGE 2 cohort which is described in section 4.21.

4.21 The EPIPAGE 2 cohort

The study will involve children included in EPIPAGE 2 from the regions participating in EPIREMED. Recruitment will be at the end of the 5 ½ year EPIPAGE 2 assessment.

The EPIPAGE 2 protocol and the main perinatal results are described in two recent publications 2015 (Ancel PY 2014, 2015).

EPIPAGE 2 (an epidemiological study of low gestational ages) is a national prospective cohort study of very premature babies recruited in France in 2011 with the objectives of:

- An improved knowledge of very premature children's futures,
- An assessment and needs forecast for medical and educational care,
- An assessment of the organization of care and medical practices and their impact for the health and development of premature infants,
- An improved knowledge concerning the causes and consequences of prematurity.

The cohort was set up in 25 regions of mainland France and overseas departments/territories. The cohort follows over 4,290 premature children up to age 12 years. Between April and December 2011 these very premature infants (between 22 and 34 weeks of amenorrhea) were included in the study. Information about pregnancy, childbirth and the immediate care of the child were collected in the maternity ward. At the end of their neonatal hospitalization, a complete assessment of their management and complications was compiled. Upon leaving the neonatal unit, all living children with parental consent were included in the follow up.

These children were reassessed at year one and two with self-administered questionnaires completed by both the parents and the attending physician. There was a follow-up rate of 90% and 87% respectively. Continuing assessments are scheduled for 2016, 2019 and 2023 when the children are 5½, 8 and 12 year old. The EPIREMED study will become involved in the 2016 framework corresponding to the 5½ years EPIPAGE evaluation of the children.

According to the needs of the study, there will be a national steering committee comprised of epidemiologists, pediatricians (Dr C Gire as a hospital-based practitioner), obstetricians, and other specialties. This committee will assume responsibility for the scientific and organizational aspects of the study. Each region will have a coordinating team comprised of pediatricians, obstetricians, midwives, and epidemiologists to oversee the study. Dr C Gire will be the PACA-region scientific coordinator. At two years, the PACA questionnaire follow-up rate is 87% for parents and 84% for physicians.

4.22 Investigators and centers

This is a multicenter, interdisciplinary study with recruitment occurring in 18 units in public academic teaching hospitals.

The **investigator coordinator** of the study is Dr Catherine GIRE, CHU Marseille, hospital Nord, AP-HM.

The **co-investigators** are (alphabetic order):

Pr Arnaud Catherine	Toulouse France Département de Santé Publique, Université de Toulouse III UMR 1027 Inserm
Pr Bednarek Nathalie	Reims France CAMSP Reims
Pr Cambonie Gilles	Montpellier France CHRU Montpellier
Pr Claris Olivier	Lyon France Hospices Civils de Lyon
Pr Debillon Thierry	Grenoble France CHRU Grenoble
Dr Foix-Hélias Laurence	Paris France APHP - Trousseau
Dr Garcia Patricia	Marseille France APHM - Conception
Dr Granier Michelle	Paris France Centre Hospitalier Sud Francilien
Pr Guillois Bernard	Caen France CHRU Caen
Pr Hascoet jean Michel	Nancy France CHRU Nancy
Pr Kuhn Pierre	Strasbourg France CHRU Strasbourg
Dr Lecomte Benedicte	Clermont-Ferrand France CHRU Clermont Ferrand
Dr Oertel Julie	Nice France CHU Nice
Pr Mitanchez Delphine	Paris France APHP - Trousseau
Pr Marret Stephane	Rouen France CHRU Rouen
Pr Patural Hugues	Saint Etienne France CHU Saint-Etienne
Dr Rubio Sophie	Lyon France Hospices Civils de Lyon
Pr Roze Jean Christophe	Nantes France CHRU Nantes
Pr Saliba Elie	Tours France CHRU tours
Dr Souksi Médioni Isabelle	Montpellier CHU
Dr Tréluyer Jean-Marc	CIC Necker APHP
Dr Zahed Meriem	Marseille France APHM - Nord

Methodological support will be provided by:

- The Clinical Research Platform of AP-HM (responsible : Pr Pascal Auquier ; medical referent : Dr Karine Baumstarck),
- INSERM unit 1153 EPOPÉ team (Dr Pierre Yves Ancel, Monique Kaminski)

4.23 Sponsor

The sponsor, in charge of all regulatory aspects, is represented by the APHM (*Assistance Publique Hôpitaux de Marseille).*

4.3 STUDY POPULATION

Eligible subjects must meet all of the following criteria.

4.31 Inclusion criteria

- Already included in the EPIPAGE 2, born between 24 and 34 weeks' GA (gestational amenorrhea),
- Children aged 5 to 6 years,
- Children exhibiting a total intellectual quotient >70 from the WPPSI IV (during the 5-year assessment in EPIPAGE 2),
- Children having a visuo-spatial Working Memory impairment defined by a working memory index <85 from the WPPSI IV,
- Children with parents (or legal guardians) authorizing participation in the study and a signed informed consent form,
- Children affiliated with medical insurance.

4.32 Non-inclusion criteria

- Children with severe cerebral palsy, based on the Gross Motor Function (GMFCS score >2) and Bimanual Fine Motor Function (BFMF >2) classification system (Elvrum AG 2015, Marois P 2015),
- Children with blindness or amblyopia, defined by a visual acuity <3 (during the 5-year assessment in EPIPAGE 2),
- Children with deafness, as defined by a prescribed hearing aid,
- Children with chromosomal disorder or autistic syndrome,
- Children included in the EPILANG study protocol (an ancillary project to EPIPAGE),
- Children who do not speak French
- Children with parents having no internet connection,
- Triplets.

4.33 Exclusion (premature study end) criteria

- Children and/or parents wishing to interrupt his/her participation during the study.

4.4 THE TWO STRATEGIES

4.41 Experimental group: the Cognitive Training

* Description of the intervention:

A Cogmed trained neuropsychologist or speech therapist, unrelated to the initial assessment, will acquaint the parents with the program which includes a software presentation, the setting of expectations, the CT objectives and a determination of the reward system along with a document to explain WM and software. He/She calendars and establishes the sessions' structure. The purpose of this initial interview is to build support for the patient and the parents.

Cogmed JM (4-7 years) is a computerized, online WM rehabilitation program. This will be usually at home, or at the hospital or in a rehabilitation center with a "tutor" according to the parents' ability and their access to an internet connected computer. The child, who is accompanied by his /her parents, or a "guardian": is given a series of interactive, automatically and individually adapted exercises. In Cogmed JM, sessions last 15-20 minutes, with three exercises out of seven for each session, with a fairground graphic interface.

The Cogmed JM practitioner consults the on-line compliance and exercise results at each session. The program calculates the performance index: the difference between the maximum level and the starting level which is used to assess progress against a standard norm. The parents benefit from a weekly 30-minute interview in order to support and strengthen the patient's motivation. The interview focuses on his/her evolving performance and rewards progress. At approximately the 15th session they are made aware of improvements in their daily lives.

* In total:

The program includes a total of three 15-minute sessions per week for eight weeks and involves :

For Children

- One medical visit and a neuropsychological examination at inclusion,
- 2 neuropsychological tests after the intervention (8 months, and 18 months after the inclusion and,
- The 8-weeks CT program.

For Parents

- An 8 weeks monitoring program,
- A weekly phone call,
- Delivering the questionnaire to the teacher at the inclusion and after eight months,
- Filling out self administered questionnaires and answering to a face to face questionnaire (at inclusion and eight months).

For teachers

Completion of the questionnaire at the inclusion and 8 months later.

4.42 Control group: current standard care management

The rehabilitation program will not be offered to this group. The children will be followed up along with their routine care management. Speech therapy and/or academic support may be recommended for those experiencing academic difficulties. The visits are the same for the children and the questionnaires are the same for parents and teacher than in the experimental group.

4.43 Total participation time

The total participation time is 18 months for both the experimental and control groups.

4.5 ENDPOINTS

4.51 Primary endpoint

The visual-spatial processing will be assessed by using the visuo-spatial index (VSI) of the Wechsler Preschool and Primary Scale of Intelligence (WPPSI IV). This index consists of two subtests : block design and object assembly. The average score is 100 with a standard deviation of 15.

Rationale for the primary endpoint:

Visuo-spatial performance is a complex mental process requiring multiple mental functions: attention, motor sensory and executive functions and WM (visuo-spatial and verbal).

Premature children perform less well to visual perceptual integration testing than those born full term and they present with a visuo constructive dyspraxia. This deficit is connected to a poor integration of visual function, perceptual and/or fine motor skills (reproduction of a complex geometric figure). This disorder is three times more frequent in adolescent very prematurely born than in adolescents born at term. Among those born extremely prematurely, 30% have results below the 15th percentile in visuo-spatial performance. (Torrioli MG 2000). The impact of WM rehabilitation on visuo-spatial skills is an interesting line of research, most particularly in premature infants.

4.52 Secondary endpoints

The secondary endpoints are related to the children and to their parents.

4.521 Children's endpoints

- The intellectual functioning and other cognitive processes will be obtained by global IQ and IQ indexes using the WPPSI IV (Wechsler) (Marley W 2014).

The WPPSI IV, designed for children ages 4 to 7, assesses the overall intellectual functioning (total comprehensive intellectual quotient : TCIQ) and specific cognitive processes. This is done through the main indices: verbal comprehension index (VCI), fluid reasoning index (FRI), visual spatial index (VSI), processing speed index (PSI), and the Working Memory index (WMI). The evaluation of the Working Memory by the WMI in WPPSI IV is uniquely visuo-spatial at this age. The average is 100 with a standard deviation of 15, as with all Wechsler Scales.

The global IQ, as well as the main indices, will be assessed when monitoring the EPIPAGE cohort.

The global IQ is lower for very premature infants as opposed to those with full-term.(AndersonPJ 2014)

- Executive and attention processes

NEPSY 2 assesses the neuropsychological development of preschool and school age children (3-12 years) and is used to obtain emerging executive functions in 5-6 year old (Korkman M 2001).

Auditory attention, statue, and design fluency are the only three subtests in the Executive and Attention Function domains, which will be administered in this study. These three tests measure selective and divided attention in auditory modality, inhibition and mental flexibility.

A review of Van de Weijer-Bergsma, which is confirmed by Mulder in a meta-analysis, shows that the selective, divided and supported attentional domains are likely to be affected in preschoolers that were prematurely (Van de Weijer-Bergsma E 2008, Mulder H 2009).

- Evaluation of language and its skills

Language is a complex mental process requiring an assessment of all its components. This assessment will be made from the Clea battery (ECPA) calibrated for ages 2-15 years (Charollais A 2014)

The battery consists of seven tests:

1 Known Digital Channels (KDC) : a reflection of verbal learning ability, both cultural and cognitive,
2 Oral Word Identification (OWI) : capacity to give a word phonological and semantic judgment;
3 Rapid Denomination (RD) : timed tested; gives an indication treatment speed,
4 Word memory (WoM) : explores the short-term mnemonic span,
5 Facial and oral praxis photographs (Prax),: relevant in motor programming,
6 Visual attention (VAtt) : inspired from NEPSY, it questions the visual spatial component,
7 Resolution of logical problems (PR) : ability to reason analogically (progressive matrices).

Many studies suggest that specific language disorders can be associated to specific WM impairment, particularly with the phonological loop (Adams AM 2000, Rodriguez A 2009).

Two meta-analyses demonstrate global language gaps/impairment in the very premature vs. the term child with deficits in learning ability, phonology, semantics, grammar, speech coherence and verbal reasonin. (Van Noort-van der speck IL 2011, Reidy N 2013).

- Behavioral Evaluation

The child's behavior will be assessed with the Goodman Strengths and Difficulties Questionnaire, which includes 25 questions answered by the parents (Goodman R 1999), in a self administered questionnaire. It will assess whether or not there is any impact of the intervention on the child's behavior.

- Evaluation of the child's quality of life (QOL)

The quality of life of the children will be assessed using the Perceived Quality of Life and Health of Adolescents and Children Questionnaire (VSP-A) (Auquier P 2001) as reported by the parents. The children's QOL will be reported by their parents.

The 49-item version for children (VSP-Ap) portrays nine dimensions and index:

- relationships with parents/family,

- body image,
- vitality,
- relationships with friends,
- general well-being,
- leisure,
- school performance
- relationships with teacher
- relationships with medical staff.

Scores range between 0 and 100, with higher scores indicating a better QOL. The French norms are available through Ravens-Sieberer U 2007.

-Schooling

Schooling will be evaluated by the GSA questionnaire (Global School Adaptation score), a French tool completed by the teacher, validated by Guimard P (2007) and re-evaluated in a preschool population in 2013 (Boussicault G 2013).

The questionnaire covers six verbal skills (verbal communication, verbal participation, vocabulary, syntax, pronunciation), five non-verbal abilities (memory, arithmetic, logical reasoning skills, manual dexterity and fine motor skills) and eight questions evaluating class behavior (compliance with rules, attention, autonomy, speed of accomplishing the task, self-esteem, ability to keep the pace and fatigability). The final question asks the teacher about possible future special educational needs of the child.

4.522 Parents'endpoints

Anxiety

The Spielberger state-trait anxiety inventory (STAI) will be used to assess anxiety. The STAI is a self-reporting questionnaire consisting of 40 items that measure both the state and trait scores. These scores range from 20 (absence of anxiety) to 80 (high anxiety) (Spielberger C 1983). This questionnaire will assess if anxiety is impacted as a result of parental intervention (mother).

Rationale for the secondary endpoints :

It is of value to measure the impact of intervention on the Working Memory and on other non-trained brain processes as well as on parental anxiety, child behavior and parental perceptions of the quality of life 'child.

V GENERAL FRAMEWORK

5.1 STEERING COMMITTEE

A steering committee will be composed of Dr Gire as coordinating investigator, two co-investigators, the EPIPAGE 2 responsible-in-chief (Dr Ancel), and a methodologist (Dr Baumstarck). The committee will assure the quality of the processes, the validation of the various steps of the study; the identification of potential study dysfunctions and means of remediation as well as the implementation of problem remediation.

5.2 IDENTIFICATION OF ELIGIBLE CHILDREN

5.21 Identifying eligible patients will be obtained during the EPIPAGE 2 group follow-up by WPPSI IV : total IQ >70 and IWM <85

* All children from the Epipage group will benefit from :

- A neuropsychological examination between the ages 5 years 6 months and 5 years 9 months. This examination will include :
    - The WPPSI IV with its main indexes,
    - Some NEPSY subtests to evaluate executive functions (inhibition, mental flexibility), social perceptions, visual-motor precision and language.

  This assessment will be made by a neuropsychologist and will last about 120 minutes.
- A medical examination including :
    - Child's health and treatment history,
    - A neurological and medical examination.

* All parents will be asked to complete the following questionnaires:

- SDQ: Child behavior,
- BRIEF: executive functions,
- SCQ (Social Communication Questionnaire),
- Autism spectrum disorders.

To ensure that the questionnaires will be properly completed, they will be checked by the regional or local coordinator accompanying the parents during the examination.

The evaluation will be carried out for research rather purposes as far as possible, testing time will be respected, and will not take more than half a day.

The tests will be performed in a predefined order, so as to homogenize testing conditions. Evaluation results and a brief written report will be presented to the parents on the examination day, with a more complete report sent by mail after discussion by the local team. The elements to be included in these reports will be discussed again. Time for test readings, scoring and report writing will be integrated in the doctors' and psychologists' remuneration.

This very comprehensive assessment will identify those children eligible for EPIREMED, that is, with a WM index lower than 85, having no exclusion criteria and whose family is able to travel to the participating testing centers.

5.22 Patient contacts

There will be a medical consultant identified at each center to optimize the implementation and the running of the study and will be the liaison between the principal medical investigator and clinical research coordinator.

The clinical research coordinator will establish a list of potentially eligible patients per month/for center in conjunction with the study national coordination. Using this list, each center's referring physician will organize the first telephone interface with those families having contact information and eligible criterion. The study's objectives, those responsible for the study, its development, and its advantages and disadvantages, will be explained. Reimbursement for travel expenses will be routinely offered. Once the family is in agreement, an appointment will be scheduled.

The family will receive a confirmation letter describing all topics discussed by telephone, the appointment date. Telephone contact will be made within two months after the identification in EPIPAGE 2 and an appointment will be set within two months.

Each center will survey the actual inclusion among all potentially eligible children (WM <85), and validate the reasons for not including the other children.

5.3 BASELINE ASSESSMENT AND INCLUSION

Eligible Epipage children with parental consent will be included after a new baseline assessment :

1. A complementary neuropsychological examination by the neuropsychologist who has done the EPIPAGE 2 evaluation which includes:

- Auditory attention (NEPSY 2),
- Language skills (ECLA battery).

2. Parents will complete the following questionnaires, and the regional or local coordinators accompanying parents will check their completion.

- Parental anxiety (Spielberger questionnaire [STAI]),
- Their children's QOL : (VSPAp)

3 A medical consultant will meet with the parents and children to ensure the criteria of inclusion and will re-explain the study.

5.4 RANDOMIZATION

A randomization list will be established before the implementation of the study with a 1:1 allocation ratio, and will be elaborated by the Clinical Research Platform, APHM (Pr Pascal Auquier).

A computer-generated randomized list using a permuted block design will be done (stratified on center and gemellarity, singleton/twin). Each center will have a specific list and inform the coordinating center of each inclusion. The inclusion form, including its date, the date of randomization, and the patient's initials will be faxed or e-mailed to the coordinating center. This list will be updated at each inclusion with the randomization date will determining the different delays.
Randomization is centralized and balanced by each center. Multiple births represent 30% of the preterm population within the EPIPAGE 2 group. If one twin has a WM anomaly, this child will be

selected for randomization. If both twins have a diminished WM; we will randomize one of the two twins in the study and the same care will be offered to the other twin.

5.5 FOLLOW UP FOR THE TWO GROUPS

Children in the Cogmed JM group will begin the program no later than two months after their inclusion, and children in the standard group will receive the routine management. A similar follow up is planned for the two groups and is based on an intermediate and final visit.

5.51 The 8 month intermediate visit

An assessment will be conducted eight months after being included in the study and will consist of :

1. The children :

- Assessment of the working memory index (WMI),
- IQ and its main indices (Weschler),
- Statue, design fluency and auditory attention : The NEPSY 3 subtests,
- Language and verbal skills (ECLA).

2. The parents:

- A self-administered questionnaire on anxiety (SDQ),
- A questionnaire assessing the behavior of the child (Goodman),
- A questionnaire assessing the child's QOL (Vspa P).

A blind evaluation will be performed by the neuropsychologists at the different EPIREMED participating centers with the group allocation of each child being unknown to the assessors.

5.52 The -18 month final visit

Children in both the intervention and control groups will receive a final evaluation 18 months after inclusion when they will be between 7 and 7 and ½ year old. A blind evaluation will be performed by the neuropsychologists at the different EPIREMED participating centers with the group allocation of each child being unknown to the assessors.

The primary evaluation end point:

The visuo-spatial Index WPPSI IV (Wechsler Preschool and Primary Scale of Intelligence) will be used for this visit. The global IQ, its indices including WMI will also be used to determine the long-term maintenance of working memory (WM) and its impact on intelligence.

VI DATA MANAGEMENT AND STATISTICAL ANALYSIS

6.1 SAMPLE SIZE

The sample size was calculated to obtain an 80% power to detect a difference of 7.5 points on the VSI index (estimated standard deviation: 15) at 18 months between the 2 groups. This has been considered to be clinically significant considering previous similar studies (Anderson PJ 2015). With the threshold for statistical significance set at a p-value of 0.05, assuming that a potential 23% of patients will be lost to follow-up between baseline and last assessments, these calculations showed that 166 patients are needed (83 per group) (Power Analysis and Sample Size Software Version 2008, Utah, USA). Assuming that 30-40% of the EPIPAGE 2 children will present with a WM abnormality, i.e., 1,600 children, recruitment should be ensured. In keeping with the highly restrictive selection criteria previously defined (see inclusion and non-inclusion criteria), recruitment is proposed as follows:

Investigators	Center	Average Expected recruitment per month	Expected recruitment
Gire Catherine<br>Zahed Meriem	Marseille France	1,5	16
Garcia Patricia	Marseille France	1,5	16
Rubio Sophie	Lyon France	0,6	8
Hascoet Jean Michel	Nancy	0.6	8
Patural Hughes	St Etienne France	0,6	8
Roze jean Christophe	Nantes France	0,6	8
Oertel Julie	Nice	0.6	8
Marret stephane	Rouen France	0,6	8
Saliba Elie	Tours France	0,6	8
Guillois Bernard	Caen France	0,6	8
Lecomte Benedicte	Clermont-Ferrand	0.6	8
Cambonie Gilles	Montpellier France	0,6	8
Kuhn Pierre	Strasbourg France	0,6	8

Bednarek Nathalie	Reims France	0,6	8
Granier Michelle	Paris France	0,6	8
Foix-hellias Laurence	Paris France	0,6	8
Mitanchez delphine	Paris France	0,6	8
Debillon Thierry	Grenoble France	0.6	8
Arnaud Catherine	Toulouse France	0.5	8
Souksi Medioni Isabelle	Montpellier France	0.5	8
TRELUYER Jean-Marc	CIC Necker APHP	0.5	8
Total			184

6.2 STATISTICAL ASPECTS

Although the analysis' main principles are reported below, a second specific protocol will be provided before the study's initialization. This will be validated by the coordinating investigator, those responsible for the analysis and the biostatistician.

Statistical analysis will be carried out by Pr. Pascal Auquier (Dr. Karine Baumstarck and Anderson Loundou and their team [Clinical research unit, DCR, APHM]). Data will be analyzed using SPSS version 17.0 software. Statistical significance is defined as $p<0.05$.

The methodology will be based on the Consolidated Standards of Reporting Trials Statement (CONSORT, http:// www.consort-statement.org/consort-statement/)(Schultz KF 2010).

The full analysis which includes all randomized subjects will be used in the primary analysis. The per protocol population (including all randomized subjects with no major protocol deviations) will be used in the secondary analysis to assess the robustness of the results. There is no interim analysis planned.

In each center the actual inclusions will be identified among all potentially eligible children (WM <85).

Demographic and baseline characteristics will be summarized. Quantitative data will be shown as mean ± standard deviation or median with its interquartile range. The qualitative data will be described as percentages with a 95% confidence interval. These data will be compared between the "control" and "experimental" groups using the chi2 test for qualitative variables and the Student t tests for continuous variables.

6.21 Primary endpoints analysis

The analysis of the primary and secondary endpoints will be done with the intent to treat. All tests used will be bilateral with a 5% significance threshold.

The mean scores of the VSI index will be compared between the 2 groups (Student t test or Mann Whitney test). Linear regression will be performed to adjust for potential confounding factors; variables relevant to the models will be selected on their clinical interest and/or a threshold p-value <= 0.1 during bivariate analysis. The final models expressed the beta standardized. The unadjusted analysis will be the primary analysis, and the adjusted analysis will be a complementary analysis.

6.22 Methodology to account for missing data

Children not meeting the primary endpoint measurements will be considered as a failure in the study regardless of their randomization group. Additional analyses will be conducted based on:

1) Available data and,

2) After multiple imputation of missing data.

6.23 Analysis of secondary endpoints

The different indices mean scores of the WPPSI, NEPSY, Clea, SDQ and GSA will be compared between the 2 groups using a Student t test or Mann-Whitney test in accordance with the variables' distribution. Anxiety and QOL scores will be compared between the 2 groups.

The comparisons of percentages will be done with the Pearson Chi-2 test or, if needed, with the Fisher exact test. All tests used will be bilateral with a significance threshold of 5%.

The scores at baseline and 8 and 18 months respectively were compared, and the analysis of variation for repeated measurements will be performed for the two groups to compare changes over time (baseline, 8 and 18 months).

6.24 Patient selection to include in the analysis

All the children registered and randomized in the study will be included in the analysis, respecting the intention to treat. This analysis will be conducted, if necessary, under the maximum bias hypothesis. In second step those wrongly included and any major protocol deviations will be excluded from the analysis.

VII ORIGINALITY AND INNOVATIVE ASPECTS

Our proposed study has different innovative levels:

- EPIPAGE 2, the national prospective cohort of very premature children offers an interesting and unique setting for the implementation of a randomized trial of CT program (EPIREMED) in premature preschoolers. This cohort provides high quality data on the perinatal period, the socio-demographic context and the child's cognitive future.

-EPIREMED is the largest, randomized, interventional study performed on the WM of a very premature birth population.

-EPIREMED, by using Cogmed JM, an on-line computerized program for WM rehabilitation, allows the parents to participate in the child's rehabilitation along with other major development actors

-EPIREMED by using Cogmed JM offers a method to improve the visual WM and possibly other untrained brain skills. Considering the cost to society and the personal investment asked of the parents, it is necessary to prove there is a high level response to this intervention. If the trial is conclusive, it would enable development of a prevention strategy by confirming one of the mechanisms associated with premature birth cognitive disorders.

-EPIREMED, which is nested in the EPIPAGE 2 study, has a follow-up until ages 8-12 years, allowing us to assess the long-term outcome for the child's cognitive performance, behavior and education. Indeed there is a need for scientific evidence regarding the effectiveness of a well-described and applicable intervention in order to improve the medium/long-term future of very premature children. This will enable us to obtain information to improve our knowledge of WM natural history of development in premature children and to identify alteration risk factors.

VIII EXPECTED FINDINGS AND INDIVIDUAL/COLLECTIVE POTENTIAL BENEFITS

This project's primary goal is to demonstrate the necessity of early visuospatial VM assessment within the vulnerable population of VP children, and to prove the feasibility and efficacy of computerized CT using online software programs.

Neurodevelopmental problems are common in VP children. Recent publications have reported many disorders in their specific cognitive functions, one of which is WM. Although neuro-development of VP children remains a public health priority because of their increased birth and survival rates, the interventions to improve this issue remain very few. There are currently no truly effective interventions to deal with academic achievement in preschool-age very preterm infants and their neuropsychological problems; thus, currently no specific management care is recommended for these children.

Cogmed is based on interactive software and parental support. The program takes into account the child's environment, a factor of great relevance since it is closely linked to the child's future development. The expected benefits of CT (Cogmed) for those children in this study are enhanced WM, possibly leading to better EF by taking advantage of cerebral plasticity. A better global neuropsychological development improvement (language and visuospatial processing) can be expected with an improvement in learning and decreased behavioral problems. For parents, their guidance is required in Cogmed and can reduce their anxiety by fully embracing their role as primary agents in their child's development. This is consistent with recommendations for family-centered healthcare and can significantly improve the quality of life in both the children and their parents. In the long term, these improvements might also reduce those global costs linked to the consequences of extreme prematurity.

Finally, if proved effective for this vulnerable population, this treatment can be a possible option or alternative in any other preschool population complaining of early academic difficulties related to WM deficits.

IX CALENDAR

The recruitment duration is estimated to be 18 months depending on the selection criteria and recruitment capacity of the centers. The maximum participation for each patient will be 18 months. The main steps of the study are presented in the following table.

Date	Action
January 2016	Legal authorizations: CPP, ANSM (French authorities)
February 2016	Investigators' meeting:<br>Validation of the study documents (CRF, consents, randomization lists)<br>Preparation for the implementation of the study within the centers<br>- Training of the neuropsychologists
October 2016	First inclusion
January 2018	Last inclusion
June 2019	End of the follow-up
July-December 2019	Quality control and data entry
January to Novembre 2020	Statistical analysis<br>Final report
May 2020	Investigators meeting:<br>Validation of the results of the final analysis<br>- Organization of the data publication

X PROJECT FAISABILITY

10.1 PARTICIPATION OF A RESEARCH NETWORK

EPIREMED is part of a research network along with EPIPAGE 2 that has already partnered in nine complementary projects (Ancel PY 2014) and is involved in the European project EPICE and the French national children cohort, ELFE.

10.2 OTHER ELEMENTS OF PROJECTS FEASIBILITY

This study is based in the EPIPAGE 2 cohort: 4,200 children, with a follow-up rate of 90 and 87% at 1 and 2 years respectively.

Families were recontacted when the child was 3 year old and will be again contacted at age of 4 (2015) to update addresses.

The study's feasibility is high since patients have already been monitored and identified and EPIREMED centers are designed to diagnose WM disorders up until 5 years and ½ of age.

EPIPAGE 2 provides an extensive neuropsychological evaluation at 5 years and ½, which serves as a prescreening. Cogmed JM has already been used in other contexts and in numerous randomized studies (Sodervisqt S 2015).

Three randomized and controlled studies similar to our population (ie preschool children) showed the effectiveness of WM rehabilitation with sustained effects and untrained brain function improvement (Nutley SB 2011, Thorell LB 2009, and Sodervisqt 2013).

Finally, the implementations by parents, key players in the child's development, along with the playful interactions with computers, which are age-appropriate, provide an assurance of compliance which can result in a successful rehabilitation.

10.3 TEAM EXPERIENCE IN THE FIELD

10.31 Coordinating investigator : C Gire

As a neonatologist and pediatric neurologist, Dr. Gire has been involved in neurological development for several years. Dr. Gire works in neonatology and is co-director of the women's parent-child center at *Hôpital Nord at AP-HM (Assistance Publique, Hôpital de Marseille*). Dr. Gire has been affiliated for several years with UPRES - EA 3279, Public health, chronic diseases, quality of life, concepts, applications and limitations, and determinants.

This team works in two main areas:

- Conceptual and methodological aspects underlying quality of life measurements in chronic diseases,
- The role of these measures in maternal and child health; mental health and its precariousness, oncology, and renal Insufficiencies.

Currently she is the principal investigator of PHCR 2009: "Quality of life of very pre-term children during elementary school": Six Level III centers (Marseille [2], Montpellier, Nantes, Nimes, and Rouen) are included in the study of 7 to 9 year old, each with an informed consent. The children were born between 1/1/2004 and 31/12/2007 before the 28th week of gestation and none with any

severe PC and/or mental deficiencies. At the end of a specific consultation the following items are identified for all participating children: QOL data (reported by children and their parents), clinical examination of the child according to “Touwen” protocol, comprehensive cognitive profile (WPPSI IV, NEPSY [executive function and focus], figure REY). The study began in 2012 and will continue until December 2015. In March 2015, the 27th month of the study, 490 parents were contacted (80% of our active files). Among these 490, 347 parents (71%) gave their consent to participate. Dr. Gire is thus expert in the organization of a multicenter study with neuropsychological assessment of children.

Additionally, she is the coinvestigator of an ancillary EPIPAGE project: EPIRMEX. This project focuses on different aspects of conventional or advanced brain MRIs at term (39-41 weeks) in a sub sample of very preterm infants included in EPIPAGE 2, and its relationship with executive functions up to 5 years et 1 /2 of age. The high number of MRIs performed in our region (130 out of 540) is testimony to Dr. Gire’s strong involvement in this study.

10.32 Science Team

10.321 Baumstarck Karine

Dr Karine Baumstarck is a physician specialized in Public Health. She works in a Hospital University Center (CHU, Assistance Publique, Hôpitaux de Marseille, France) in the Clinical Research Unit as methodologist. She supports different investigators in the structuration of their clinical research projects. She also is affiliated to a University Department (Aix Marseille University, France), EA 3279, Quality of life and chronic diseases, responsible Prof. Pascal Auquier.

10.322 Kaminski Monique

Dr. Kaminski, Director Emeritus of research at INSERM’s EPOPÉ team, UMR 1153, is an epidemiologist in perinatal and children’s health; and works on the long-term development of very premature children. Dr. Kaminski was involved in implementation and data analysis of such major chort study as EPIPAGE 1, EDEN, and is currently a member of the EPIPAGE 2 scientific committee.

10.323 Ancel Pierre Yves

Dr. Ancel is MCU-PH (*Maître de conférence des universités – praticien hospitalier*) at the University Paris V; Director of the Obstetric Epidemiology Team, Perinatal and Pediatric, EPOPé, UMR 1153 Inserm. Dr. Ancel is a perinatal care epidemiologist and a participant in the EPIPAGE 1 cohort as well as the manager/national coordinator of EPIPAGE 2.

10.324 Calderon Johanna

Calderon Johanna neuropsychologist is responsible for a Boston study that is assessing the Cogmed program on children with cardiac malformations

10.33 Investigators and associated members of the steering committee EPIPAGE 2

The EPIREMED study is part of EPIPAGE 2 and allows us to benefit from the collaboration of those involved in the EPIPAGE 2 cohort's steering committee. The different investigators are involved in the monitoring and care of very premature babies. Their diverse training and responsibilities meet all aspects of our project. These investigators are either pediatrician-neonatologists or pediatric neurologists working in type III neonatal units, in neuro-pediatric services for very premature infants’s assessment or in a referral center for learning disabilities.

10.4 FINAL REPORT AND PUBLICATION

In accordance with Article R 5121-13 of the Public Health Code, clinical studies prior to their publication are not subject to any written or oral comments without the joint agreement of the coordinating investigator and the promoter.

Any publication must state that APHM is the promoter and the study's databases will be co-owned by the promoter APHM and by the INSERM U1153, EPOPÉ team. The results, as well as all research-related data, must under no circumstances be transmitted to third parties without compensation negotiated beforehand by the Medical Research Branch of the APHM and/or INSERM U 1153

All data, results, inventions and discoveries resulting from the study will automatically become the exclusive property of the sponsor which may use this information in the manner deemed suitable with the agreement of the investigator.

Publication requirements: all results of an APHM funded clinical study or a request for proposal is under the scientific responsibility of the coordinating investigator, and these results must be made public if they have sufficient scientific validity. Any request to hide the results, change and mitigate the contents of the final report and publication will be rejected by the promoter. Publications must describe in an honest and balanced way all aspects of the study regardless of other interests, particularly those of the non-scientists. The publications' authors must comply with the quality rules of scientific journals.

10.5 CASE REPORTS FORM

All information collected by the overseeing practitioner will be transcribed in the case report. The data, whether clinical or non-clinical, will be transferred as the data are obtained.

XI REGULATORY AND ETHICAL ASPECTS

11.1 APPROVALS

The expected sponsor is represented by the *Assistance Publique-Hôpitaux de Marseille* and must have approvals from the French authorities prior to the study's initiation. The sponsor and the investigators will conduct the study in accordance with Good Clinical Practices and the French applicable regulatory requirements (*Code de la Santé Publique*, article L.1121-1/Public Health Act No. 2004-806 of 9 August 2004 on public health policy and its implementing decrees of August 27, 2006), as the applicable privacy requirements and ethical principles outlined in the Declaration of Helsinki. The protocol and amendments will be approved by the French *Comité de Protection des Personnes* (Protection to Persons committee) and by the *Agence Nationale de Sécurité des Médicaments.* (national agency for drug safety). Participation is voluntary and all patients will submit a written informed consent. Protection of the rights will be guaranteed, and anonymity conserved. The study's results will be in the form of feedback to the patients.

11.2 CONFIDENTIALY

In accordance with French laws and regulations respectively (Law of August 9, 2004, Computer Law and Liberties of January 6, 1978, as amended by the Act of 1 July 1994 and Decree of May 9, 1995) and (National Data Processing and Liberties Commission and the Advisory Committee on the treatment of research information in the health field: Decision of 5 January 2006. Reference Methodology MR-001)): all patient records identities will remain confidential.

11.3 OTHER REGULATORY ASPECTS

Management of protocol amendments and adverse events will follow the regulatory procedures of the sponsor.

11.31 Monitoring and quality control

In accordance with applicable regulations including Good Clinical Practices, and Sponsor's procedures, a Sponsor's monitor will visit the investigator before, during and after the study. Sponsor's monitors will contact the site prior to the start of the study to review with the site staff the protocol, study requirements, and their responsibilities to satisfy regulatory, ethical, and Sponsor's requirements. When reviewing data collection procedures, the discussion will also include identification, agreement and documentation of data items for which the CRF will serve as the source document.

Sponsor will monitor the study and site activity to verify that the:

- Data are authentic, and complete.
- Safety and rights of subjects are being protected (obtaining consents and recording prior to their participation in the trial).
- Study is conducted in accordance with the currently approved protocol and any other study agreements, GCP, and all applicable regulatory requirements.

The investigator and the head of the medical institution agrees to allow the monitor direct access to all relevant documents

The type and frequency of monitoring are determined using the definition of the level of monitoring based on patient risk and depend on the number of patients included, the rate of inclusions and difficulties encountered during the conduct of the study (procedures approved by the group quality promotion FHF which determines the level of monitoring to be carried out based on risk to the subject-OECD recommendation on the Governance of Clinical Trials, December 2012).
In this study, the level of monitoring is rated “minimal” with a risk for the patient level A. The Sponsor’s monitor will verify the signed informed consents. If one / multiple consents are not compliant, files will be monitored randomly.

The protocol’s study information will be posted on **clinicaltrials.gov** before subjects are enrolled.

11.32 Vigilance of clinical trials

Definitions

Adverse event (AE)

Any noxious or undesirable event experienced by a participant during a clinical trial, whether or not considered related to the protocol should be considered as an adverse event.

Serious adverse event (SAE)

A serious adverse event is an adverse event that is:

- Fatal
- life-threatening
- significantly, persistently or permanently disabling
- requiring in-patient hospitalization or prolongation of hospitalization
- requiring intervention to prevent permanent impairment or damage
- Any event that could be considered as potentially harmful
- Any event medically accurate according to the investigator’s judgment

In addition, congenital abnormalities, occurrence of malignancy or clinical injuries resulting from overdose are always considered as SAE.

Responsibilities of the investigator

Modalities of collection, verification and presentation of adverse events

All adverse events occurring during clinical trials have to be collected, verified, registered and reported from Day 0 (inclusion day for the participant) until the last day of the study, or as soon as the investigator becomes aware of the AE that he considers linked to the protocol and this up to its resolution.
Adverse events are collected:

- During clinical examination, from blood sample analysis, or from the investigator questioning the patient
- from the patient’s unsolicited reporting, as encouraged to do towards the investigator

Serious adverse events reporting (SAE)

The investigator must assess the SAE in severity and report it to the sponsor within 24 hours of his/her becoming aware of it (SAE Form at the end of the CRF).

The investigator must search for evidence of a causality between the protocol and the SAE, and supply when possible a medical prognosis. The investigator must provide information on symptoms, time of onset, subsidence, action taken and subject outcome.

The SAE Form must be sent to the sponsor together with the hospital reports, examination reports and biological results related to the SAE (including negative results); Single patient reports must be anonymised and bear a code/randomization number.

Once completed, dated and signed, the Serious Adverse Event Form must be addressed by fax to:

Direction of Clinical Research and Innovation of AP-HM
80, rue Brochier, 13354 Marseille Cedex 05
Phone 04 91 38 27 47 Fax: 04 91 38 14 79
E-mail: drci@ap-hm.fr

All subjects with SAE must be followed up for outcome until resolution (even if the patient dropped out/was excluded from the study); reports must be sent to the sponsor within 8 days of the initial SAE declaration by fax or email.

Causality link

The investigator must assess the causality link between the SAE and the protocol. If a causality link is suspected, the investigator/sponsor will regard the SAE as a Suspected Unexpected Serious Adverse Reaction (SUSAR).

Declaration period

SAEs must be reported for a participant:

- From the day he signed the consent form
- For the duration of the follow-up as defined in the study protocol
- Up to 4 weeks after the end of the study protocol
- Anytime, as soon as the investigator becomes aware of it and if a causality link has been evidenced between the protocol and the SAE.

Responsibilities of the sponsor

Unexpected SAE declaration

The sponsor must assess the causality link between the SAE and the protocol.

The sponsor will also report all expected/unexpected SAE based on the SPC (Summary of Product Characteristics).

The sponsor will assume the responsibility for appropriate reporting of adverse events to Ethics Committees and relevant Health Authorities.

The regulatory delays for SAE declaration are:

- 7 days for SAE, fatal SUSARs or life-threatening SUSARs. A further delay of 8 days is granted for full and accurate documentation of the case
- 15 days for all other SUSARs. A further delay of 8 days is granted for full and accurate documentation of the case
- In double-blinded studies, the sponsor must report the SUSAR to the relevant Health Authorities and Ethics committees as soon as possible after the unblinding visit.

New Safety Data declaration

The sponsor must declare any new safety data and send an Annual Security Report to the relevant Health Authorities and Ethics committees.

Annual Security Report

At the date of the anniversary for the trial authorization issued by Health Authorities and Ethics Committees, the sponsor must write a safety report including:

- The list of serious adverse event that may be linked to the protocol including unexpected and expected serious events.
- A critical assessment of patient safety suitable for research.

This report can be submitted for approval to the principal investigator.

The sponsor must send the Annual Security Report within 60 days of the trial authorization.

Data Safety Monitoring Board

A committee of Data and Safety Monitoring Board will not be formed for this protocol.

XII REFERENCES

Aarnoudse-Moens CS, Weisglas-Kuperus N, van Goudoever JB, Oosterlaan J. Meta-analysis of neurobehavioral outcomes in very preterm and/or very low birth weight children. Pediatrics. 2009 Aug;124(2):717-28

Adams AM, Gathercole SE. Limitations in working memory: implications for language development. Int J Lang Commun Disord. 2000 Jan-Mar;35(1):95-116

Alloway TP, Alloway RG Investigating the predictive roles of working memory and IQ in academic attainment.J Exp Child Psychol. 2010 May;106(1):20-9

Alloway TP, Gathercole SE, Pickering SJVerbal and visuospatial short-term and working memory in children: are they separable? Child Dev. 2006 Nov-Dec;77(6)

Ancel PY, Goffinet F; EPIPAGE 2 Writing Group .EPIPAGE 2: a preterm birth cohort in France in 2011.. BMC Pediatr. 2014 Apr 9;14:97

Ancel PY, Goffinet F; EPIPAGE-2 Writing Group, Kuhn P, Langer B, Matis J, Hernandorena X, Chabanier P, Joly-Pedespan L, Lecomte B, Vendittelli F, Dreyfus M, Guillois B, Burguet A, Sagot P, Sizun J, Beuchée A, Rouget F, Favreau A, Saliba E, Bednarek N, Morville P, Thiriez G, Marpeau L, Marret S, Kayem G, Durrmeyer X, Granier M, Baud O, Jarreau PH, Mitanchez D, Boileau P, Boulot P, Cambonie G, Daudé H, Bédu A, Mons F, Fresson J, Vieux R, Alberge C, Arnaud C, Vayssière C, Truffert P, Pierrat V, Subtil D, D'Ercole C, Gire C, Simeoni U, Bongain A, Sentilhes L, Rozé JC, Gondry J, Leke A, Deiber M, Claris O, Picaud JC, Ego A, Debillon T, Poulichet A, Coliné E, Favre A, Fléchelles O, Samperiz S, Ramful D, Branger B, Benhammou V, Foix-L'Hélias L, Marchand-Martin L, Kaminski M. Survival and morbidity of preterm children born at 22 through 34 weeks' gestation in France in 2011: results of the EPIPAGE-2 cohort study. JAMA Pediatr. 2015 Mar;169 (3):230-8

Anderson PJ .Neuropsychological outcomes of children born very preterm. Semin Fetal Neonatal Med. 2014 Apr;19(2):90-6

Anderson PJ, Doyle LW; Victorian Infant Collaborative Study Group Executive functioning in school-aged children who were born very preterm or with extremely low birth weight in the 1990s.Pediatrics. 2004 Jul;114(1):50-7

Anderson PJ, Reidy N.. Assessing executive function in preschoolers. Neuropsychol Rev. 2012 Dec;22(4):345-60

Auquier P, Clement A, C Sapin, El Khammar M, San Macro JL, Siméoni MC: Development, validation of HRQL measurement in children: VSP-Ae. Qual Life Res 2001, 10: 268

Auquier P, Clément A, Sapin C, El Khammar M, San Marco JL, Siméoni MC: Development, validation of HRQL measurement in children: VSP-Ae. Qual Life Res 2001, 10:268

Aylward GP Cognitive and neuropsychological outcomes: more than IQ scores. Ment Retard Dev Disabil Res Rev. 2002;8(4):234-40

Baddeley A. The fractionation of working memory. Proc Natl Acad Sci U S A. 1996 Nov 26;93(24)

Baron IS, Erickson K, Ahronovich MD, Litman FR, Brandt J. Spatial location memory discriminates children born at extremely low birth weight and late-preterm at age three. Neuropsychology. 2010 Nov;24(6):787-94

Bergman Nutley S, Söderqvist S, Bryde S, Thorell LB, Humphreys K, Klingberg T Gains in fluid intelligence after training non-verbal reasoning in 4-year-old children: a controlled, randomized study. Dev Sci. 2011 May;14(3):591-601

Bhutta AT, Cleves MA, Casey PH, Cradock MM, Anand KJ Cognitive and behavioral outcomes of school-aged children who were born preterm: a meta-analysis. JAMA. 2002 Aug 14;288(6):728-37

Bierman Kl , Torres M . Promoting the development of executive functions though early education and prevention programs. In J.A Griffin LS. Freund and P McCardle (eds) Executive function in preschool age children: integrating mesasurement , neurodevelopment and translational research. Washington , DC : American psychological Associataion (2015)

Boivin MJ, Busman RA, Parikh SM, Bangirana P, Page CF, Opoka RO, Giordani B. A pilot study of the neuropsychological benefits of computerized cognitive rehabilitation in Ugandan children with HIV. Neuropsychology. 2010 Sep;24(5):667-73
Borella E, Carretti B, Riboldi F, De Beni R. Working memory training in older adults: evidence of transfer and maintenance effects .Psychol Aging. 2010. Dec;25(4):767-78
Borradori Tolsa C, Barisnikov K, Lejeune F, Hüppi P.Development of executive functions in preterm children]. Arch Pediatr. 2014 Sep;21(9):1035-40
Borteyrou X, Bruchon-Schweitzer M, Spielberger CD. [The French adaptation of the STAXI-2, C.D. Spielberger's State-trait anger expression inventory]. Encephale. 2008 Jun;34(3):249-55
Boussicault G, Nguyen The Tich S, Branger B, Guimard P, Florin A, Rozé JC, Flamant C.The Global School Adaptation score: a new neurodevelopmental assessment tool for very preterm children at five years of age. J Pediatr. 2013 Aug;163(2):460-4
Boyle MH, Miskovic V, Van Lieshout R, Duncan L, Schmidt LA, Hoult L, Paneth N, Saigal S.Psychopathology in young adults born at extremely low birth weight. Adolesc Med State Art Rev. 2013 Apr;24(1):330-40
Braun J, Weyhreter H, kohnlein O, storck M, Bode H : kognitives training: ein program zur forderung von vorschulkindern mit intellectual defitizen . psychologie in Erziehung und Unterricht ; 47 : 10-7
Brown RT, Wynne ME, Borden KA, Clingerman SR, Geniesse R, Spunt AL Methylphenidate and cognitive therapy in children with attention deficit disorder: a double-blind trial. J Dev Behav Pediatr. 1986 Jun;7(3):163-74
Butler RW, Copeland DR, Fairclough DL, Mulhern RK, Katz ER, Kazak AE, Noll RB, Patel SK, Sahler OJ. A multicenter randomized clinical trial of a cognitive remediation program for childhood survivors of a pediatric malignancy. J Consult Clin Psychol. 2008 Jun;76(3):367-78
Charollais A, Stumpf MH, De Quelen R, Rondeau S, Pasquet F, Marret S; Perinatal Network of Haute-Normandie Delayed language development at two years of age in very preterm infants in the Perinatal Network of Haute-Normandie. Early Hum Dev. 2014 Dec;90(12):891-2
Clark CA, Woodward LJ. Neonatal cerebral abnormalities and later verbal and visuospatial working memory abilities of children born very preterm. Dev Neuropsychol. 2010 2010;35(6):622-42
Cortese S, Ferrin M, Brandeis D, Buitelaar J, Daley D, Dittmann RW, Holtmann M, Santosh P, , Stevenson J, Stringaris A, Zuddas A, Sonuga-Barke EJ; European ADHD Guidelines Group (EAGG Cognitive training for attention-deficit/hyperactivity disorder: meta-analysis of clinical and neuropsychological outcomes from randomized controlled trials. J Am Acad Child Adolesc Psychiatry. 2015 Mar;54(3):164-74
Cowan N. Working Memory Underpins Cognitive Development, Learning, and Education..Educ Psychol Rev. 2014 Jun 1;26(2):197-223
Cowan N. Working Memory Underpins Cognitive Development, Learning, and Education. Educ Psychol Rev. 2014 Jun 1;26(2):197-223
Curtis WJ, Lindeke LL, Georgieff MK, Nelson CA Neurobehavioural functioning in neonatal intensive care unit graduates in late childhood and early adolescence. Curtis WJ, Lindeke LL, Georgieff MK, Nelson CA. Brain. 2002 Jul;125(Pt 7):1646-59
De Ribaupierre A, Bailleux C The development of working memory: further note on the comparability of two models of working memory. J Exp Child Psychol. 2000 Oct;77(2):110-27
Del Giudice E, Grossi D, Angelini R, Crisanti AF, Latte F, Fragassi NA, Trojano L.. Spatial cognition in children. I. Development of drawing-related (visuospatial and constructional) abilities in preschool and early school years. Brain Dev. 2000 Sep;22(6):362-7
Del Giudice E, Grossi D, Angelini R, Crisanti AF, Latte F, Fragassi NA, Trojano L.. Spatial cognition in children. I. Development of drawing-related (visuospatial and constructional) abilities in preschool and early school years. Brain Dev. 2000
Del Giudice E, Trojano L, Fragassi NA, Posteraro S, Crisanti AF, Tanzarella P, Marino A, Grossi D Spatial cognition in children. II. Visuospatial and constructional skills in developmental reading disability..Brain Dev. 2000 Sep;22(6):368-72

Diamond A.Executive functions. Annu Rev Psychol. 2013;64:135-68
Doyle LW, Anderson PJ, Battin M, Bowen JR, Brown N, Callanan C, Campbell C, Chandler S, Cheong J, Darlow B, Davis PG, DePaoli T, French N, McPhee A, Morris S, O'Callaghan M, Rieger I, Roberts G, Spittle AJ, Wolke D, Woodward LJLong term follow up of high risk children: who, why and how?. BMC Pediatr. 2014 Nov 17;14:279
Elvrum AG, Andersen GL, Himmelmann K, Beckung E, Öhrvall AM, Lydersen S, Vik T. Bimanual Fine Motor Function (BFMF) Classification in Children with Cerebral Palsy: Aspects of Construct and Content Validity. Phys Occup Ther Pediatr. 2015 Aug 19:1-16
Espy KA, Bull R, Martin J, Stroup W.Measuring the development of executive control with the shape school. Psychol Assess. 2006 Dec;18(4):373-81
Espy KA, McDiarmid MM, Cwik MF, Stalets MM, Hamby A, Senn TE. The contribution of executive functions to emergent mathematic skills in preschool children. Dev Neuropsychol. 2004;26(1):465-86
Galbiati S, Recla M, Pastore V, Liscio M, Bardoni A, Castelli E, Strazzer S.Attention remediation following traumatic brain injury in childhood and adolescence. Neuropsychology. 2009 Jan;23(1):40-9
Gathercole SE, Alloway TP, Willis C, Adams AM Working memory in children with reading disabilities.J Exp Child Psychol. 2006 Mar;93(3):265-81
Gathercole SE, Pickering SJ, Ambridge B, Wearing H The structure of working memory from 4 to 15 years of age.. Dev Psychol. 2004 Mar;40(2):177-90
Gathercole SE, Pickering SJ.Working memory deficits in children with low achievements in the national curriculum at 7 years of age. Br J Educ Psychol. 2000 Jun;70 (Pt 2):177-94
Goodman R, S Scott. Comparing the Strengths and Difficulties Questionnaire and the Child Behavior Checklist: is small beautiful? J Abnorm Child Psychol 1999; 27(1):17-24
Goodman R. The extended version of the Strengths and Difficulties Questionnaire as a guide to child psychiatric caseness and consequent burden. J Child Psychol Psychiatry 1999; 40(5):791-9
Goodman R. The Strengths and Difficulties Questionnaire: a research note. J Child Psychol Psychiatry 1997; 38(5):581-6
Grunau RE, Whitfield MF, Davis C Pattern of learning disabilities in children with extremely low birth weight and broadly average intelligence. Arch Pediatr Adolesc Med. 2002 Jun;156(6):615-20
Grunewaldt KH, Løhaugen GC, Austeng D, Brubakk AM, Skranes J. Working memory training improves cognitive function in VLBW preschoolers. Pediatrics. 2013 Mar;131(3):e747-54
Grunewaldt KH, Skranes J, Brubakk AM, Lähaugen GC Computerized working memory training has positive long-term effect in very low birthweight preschool children. Dev Med Child Neurol. 2015 Jul 3
Guimard P, Cosnefroy O, Florin A. Teacher rating of behaviors and competences, and prediction of academic performances and of later shool pathways in primary and secondary school. L 'orientation scolaire et professionelle 2007 ; 36:179-202.
Halperin JM, Marks DJ, Bedard AC, Chacko A, Curchack JT, Yoon CA, Healey DM. Training executive, attention, and motor skills: a proof-of-concept study in preschool children With ADHD. J Atten Disord. 2013 Nov;17(8):711-21
Hutchinson EA[1], De Luca CR, Doyle LW, Roberts G, Anderson PJ; Victorian Infant Collaborative Study Group .School-age outcomes of extremely preterm or extremely low birth weight children. Pediatrics 2013 : 131 : E1053-61
Jarjour IT. Neurodevelopmental outcome after extreme prematurity: a review of the literature. Pediatr Neurol. 2015 Feb;52(2):143-52
Johnson S, Wolke D, Hennessy E, Marlow N .Educational outcomes in extremely preterm children: neuropsychological correlates and predictors of attainment..Dev Neuropsychol. 2011;36(1):74-95
Johnson S. Cognitive and behavioural outcomes following very preterm birth. Semin fetal neonat med 2007;12:363-73
Kail R. the development of memory in children . troisième edition. NY: wh Freeman 1990
Karch D, Albers L, Renner G, Lichtenauer N, von Kries R The efficacy of cognitive training programs in children and adolescents: a meta-analysis. Dtsch Arztebl Int. 2013 Sep;110(39):643-52

Kerr-Wilson CO, Mackay DF, Smith GC, Pell JP. Meta-analysis of the association between preterm delivery and intelligence. J Public Health (Oxf). 2012 Jun;34(2):209-16
Kibby MY, Marks W, Morgan S, Long CJ. Specific impairment in developmental reading disabilities: a working memory approach.. J Learn Disabil. 2004
Kirk HE, Gray K, Riby DM, Cornish KM Cognitive training as a resolution for early executive function difficulties in children with intellectual disabilities. Res Dev Disabil. 2015 Mar;38:145-60
Klingberg T, Forssberg H, Westerberg H Increased brain activity in frontal and parietal cortex underlies the development of visuospatial working memory capacity during childhood..J Cogn Neurosci. 2002 Jan 1;14(1):1-10
Korkman M, Kemp SL, Kirk U Effects of age on neurocognitive measures of children ages 5 to 12: a cross-sectional study on 800 children from the United States. Dev Neuropsychol. 2001;20(1):331-54
Kozulin A, Lebeer J, Madella-Noja A, Gonzalez F, Jeffrey I, Rosenthal N, Koslowsky M.Cognitive modifiability of children with developmental disabilities: a multicentre study using Feuerstein's Instrumental Enrichment--Basic program. Res Dev Disabil. 2010 Mar-Apr;31(2):551-9
Larroque B, Ancel PY, Marret S, Marchand L, André M, Arnaud C, Pierrat V, Rozé JC, Messer J, Thiriez G, Burguet A, Picaud JC, Bréart G, Kaminski M; EPIPAGE Study group.. Neurodevelopmental disabilities and special care of 5-year-old children born before 33 weeks of gestation (the EPIPAGE study): a longitudinal cohort study. Lancet. 2008 Mar 8;371(9615):813-20
Litt J, taylor Hg, klein N, hack M. Learning disability in children with very low birth weight : prevalence,neuropsychological correlates and educational interventions. J learn disabil 2005; 158: 15-20
Logie RH. The seven ages of working memeory. In : richarson JTE; Engle, RW; hasher, L ; Logie Rh Stoltzfus Er, Zacks, Rt editors . wokring memeory and human cognition. New York oxford university press; 1996.p 31-65
Løhaugen GC, Antonsen I, Håberg A, Gramstad A, Vik T, Brubakk AM, Skranes J.Computerized working memory training improves function in adolescents born at extremely low birth weight. J Pediatr. 2011 Apr;158(4):555-561
Marlow N, Wolke D, Bracewell MA, Samara M; EPICure Study Group. Neurologic and developmental disability at six years of age after extremely preterm birth..N Engl J Med. 2005 Jan 6;352(1):9-19
Marois P, Marois M, Pouliot Laforte A, Vanasse M, Lambert J, Ballaz L. Gross Motor Function Measure Evolution Ratio: Use as a control for natural progression in cerebral palsy. Arch Phys Med Rehabil. 2015 Aug 17
Marret S, Ancel PY, Marchand L, Charollais A, Larroque B, Thiriez G, Alberge C, Pierrat V, Rozé JC, Fresson J, Bréart G, Kaminski M; groupe EPIPAGE [Special outpatient services at 5 and 8 years in very-preterm children in the EPIPAGE study]. Arch Pediatr. 2009 Sep;16 Suppl 1:S17-27
Marret S, Chollat C, de Quelen R, Pinto Cardoso G, Abily-Donval L, Chadie A, Torre S, Vanhulle C, Mellier D, Charollais A, Ancel PY; Réseau de périnatalité en Haute-Normandie. [Course and neurological/behavioral development of preterm children]. Arch Pediatr. 2015 Feb;22(2):195-202
Mathiasen R, Hansen BM, Nybo Anderson AM, Greisen G Socio-economic achievements of individuals born very preterm at the age of 27 to 29 years: a nationwide cohort study..Dev Med Child Neurol. 2009 Nov;51(11):901-8
Melby-Lervåg M, Hulme C Is working memory training effective? A meta-analytic review..Dev Psychol. 2013 Feb;49(2):270-91
Mulder H, Pitchford NJ, Hagger MS, Marlow N.Development of executive function and attention in preterm children: a systematic review. Dev Neuropsychol. 2009;34(4):393-421
Mulder H, Pitchford NJ, Marlow N. Inattentive behaviour is associated with poor working memory and slow processing speed in very pre-term children in middle childhood. Br J Educ Psychol. 2011 Mar;81(Pt 1):147-60
Mulder H, Pitchford NJ, Marlow N. Processing speed and working memory underlie academic attainment in very preterm children. Arch Dis Child Fetal Neonatal Ed. 2010

Mulder H, Pitchford NJ, Marlow N. Processing speed mediates executive function difficulties in very preterm children in middle childhood. J Int Neuropsychol Soc. 2011 May;17(3):445-54
Nix RL, Bierman KL, Domitrovich CE, Gill S. Promoting Children's Social-Emotional Skills in Preschool Can Enhance Academic and Behavioral Functioning in Kindergarten: Findings from Head Start REDI.. Early Educ Dev. 2013;24(7)
Nosarti C, Giouroukou E, Micali N, Rifkin L, Morris RG, Murray RM. Impaired executive functioning in young adults born very preterm. J Int Neuropsychol Soc. 2007 Jul;13(4):571-81
Omizzolo C, Scratch SE, Stargatt R, Kidokoro H, Thompson DK, Lee KJ, Cheong J, Neil J, Inder TE, Doyle LW, Anderson PJ. Neonatal brain abnormalities and memory and learning outcomes at 7 years in children born very preterm. Memory. 2014;22(6):605-15
Pascoe L, Roberts G, Doyle LW, Lee KJ, Thompson DK, Seal ML, Josev EK, Nosarti C, Gathercole S, Anderson PJ. Preventing academic difficulties in preterm children: a randomised controlled trial of an adaptive working memory training intervention - IMPRINT study. BMC Pediatr. 2013 Sep 16;13:144
Pickering SJ Development of memory for pattern and path: further evidence for the fractionation of visuo-spatial memory. Gathercole SE, Hall M, Lloyd SA.. Q J Exp Psychol A. 2001 May;54(2):397-420
Pickering SJ. The development of visuo-spatial working memory. Memory. 2001 Jul;9(4):423-432
Rabiner DL, Murray DW, Skinner AT, Malone PS. A randomized trial of two promising computer-based interventions for students with attention difficulties. J Abnorm Child Psychol. 2010 Jan;38(1):131-42
Rai E· Long-term outcomes of prematurity in adolescence. Psychol Med. 2011 Aug;41(8):1763-74
Rapport MD, Orban SA, Kofler MJ, Friedman LM. Do programs designed to train working memory, other executive functions, and attention benefit children with ADHD? A meta-analytic review of cognitive, academic, and behavioral outcomes. Clin Psychol Rev. 2013
Ravens-Sieberer U, Auquier P, Erhart M, Gosch A, Rajmil L, Bruil J, Power M, Duer W, Cloetta B, Czemy L, Mazur J, Czimbalmos A, Tountas Y, Hagquist C, Kilroe J; European KIDSCREEN Group. The KIDSCREEN-27 quality of life measure for children and adolescents: psychometric results from a cross-cultural survey in 13 European countries. Qual Life Res. 2007 Oct;16(8):1347-56
Reidy N, Morgan A, Thompson DK, Inder TE, Doyle LW, Anderson PJ. Impaired language abilities and white matter abnormalities in children born very preterm and/or very low birth weight. J Pediatr. 2013 Apr;162(4):719-24
Robin J, Parkinson J. The potential for school based interventions that target executive function to improve academic achievement : a review. Review of educational research. À paraitre 2015
Rodrigues A, Befi-Lopes DM..Phonological working memory and its relationship with language development in children. Pro Fono. 2009 Jan-Mar;21(1):63-8
Rose SA, Feldman JF, Jankowski JJ, Van Rossem R Basic Information Processing Abilities at 11 years Account for Deficits in IQ Associated with Preterm Birth. Intelligence. 2011 Jul;39(4):198-209

Rose SA, Feldman JF, Jankowski JJ, Van Rossem R The structure of memory in infants and toddlers: an SEM study with full-terms and preterms.. Dev Sci. 2011 Jan;14(1):83-91
Rueda MR, Rothbart MK, McCandliss BD, Saccomanno L, Posner MI. Training, maturation, and genetic influences on the development of executive attention
Rushe TM, Rifkin L, Stewart AL, Townsend JP, Roth SC, Wyatt JS, Murray RM. Neuropsychological outcome at adolescence of very preterm birth and its relation to brain structure. Dev Med Child Neurol. 2001 Apr;43(4):226-33
Saavalainen PM, Luoma L, Laukkanen E, M Bowler D, Määttä S, Kiviniemi V, Herrgård E. School performance of adolescents born preterm: neuropsychological and background correlates. Eur J Paediatr Neurol. 2008 May;12(3):246-52
Saigal S, Stoskopf B, Streiner D, Boyle M, Pinelli J, Paneth N, Goddeeris J Transition of extremely low-birth-weight infants from adolescence to young adulthood: comparison with normal birth-weight controls.JAMA. 2006 Feb 8;295(6):667-75

Schulz KF, Altman DG, Moher D. CONSORT 2010 statement: Updated guidelines for reporting parallel group randomised trials .J Pharmacol Pharmacother. 2010 Jul;1(2):100-7

Shinaver CS 3rd, Entwistle PC, Söderqvist S. Cogmed WM training: reviewing the reviews. Appl Neuropsychol Child. 2014;3(3):163-72

Söderqvist S, Nutley SB, Ottersen J, Grill KM, Klingberg T. Computerized training of non-verbal reasoning and working memory in children with intellectual disability. Front Hum Neurosci. 2012. Oct 2;6:271

Spencer-Smith M, Klingberg T. Benefits of a working memory training program for inattention in daily life: a systematic review and meta-analysis.PLoS One. 2015 Mar 20;10(3)

St Clair-Thompson HL , stevens R, hunt A et bolder E. Improving children’s working memory and classroom performance. Eductaionnal psychology 2010, 30, 203-219

Suchan B Neuroanatomical correlates of processing in visual and visuospatial working memory. Cogn Process. 2008 Mar;9(1):45-51

Swanson HL, Sachse-Lee C Mathematical problem solving and working memory in children with learning disabilities: both executive and phonological processes are important.J Exp Child Psychol. 2001 Jul;79(3):294-321

Swanson HL. Intellectual growth in children as afunction of domain specific and domain general working memeory subgroups. Intelligence , 2011. 39, 481-492

Swanson HL.Working memory and phonological processing as predictors of children's mathematical problem solving at different ages. Mem Cognit. 2004 Jun;32(4):648-61

Taylor HG, Minich NM, Klein N, Hack M Longitudinal outcomes of very low birth weight: neuropsychological findings. J Int Neuropsychol Soc. 2004

Taylor HG. Children born preterm or with very low birth weight can have both global and selective cognitive deficits. J Dev Behav Pediatr. 2006 Dec;27(6):485-6

Thorell LB , lindqvist S, Bergmen S, bohlin NG,, klingberg T. training and transfer of executive functions in preschool children developmental science 2009, 12, 106-113

Torchin H, Ancel PY, Jarreau PH, Goffinet F. [Epidemiology of preterm birth: Prevalence, recent trends, short- and long-term outcomes]. J Gynecol Obstet Biol Reprod (Paris). 2015 Jul 1

Torrioli MG, Frisone MF, Bonvini L, Luciano R, Pasca MG, Lepori R, Tortorolo G, Guzzetta FPerceptual-motor, visual and cognitive ability in very low birthweight preschool children without neonatal ultrasound abnormalities. Brain Dev. 2000 May;22(3):163-8

Van de Weijer-Bergsma E, Wijnroks L, Jongmans MJ. Attention development in infants and preschool children born preterm: a review. Infant Behav Dev. 2008 Sep;31(3):333-51

Van Noort-van der Spek IL, Franken MC, Weisglas-Kuperus N .Language functions in preterm-born children: a systematic review and meta-analysis. Pediatrics. 2012 Apr;129(4):745-54

Watkins MW, Beaujean AA. Bifactor structure of the Wechsler Preschool and Primary Scale of Intelligence--Fourth Edition. Sch Psychol Q. 2014 Mar;29(1):52-63

Zwicker JG, Harris SR. Quality of life of formerly preterm and very low birth weight infants from preschool age to adulthood: a systematic review. Pediatrics. 2008 Feb;121(2)

XIII APPENDIX

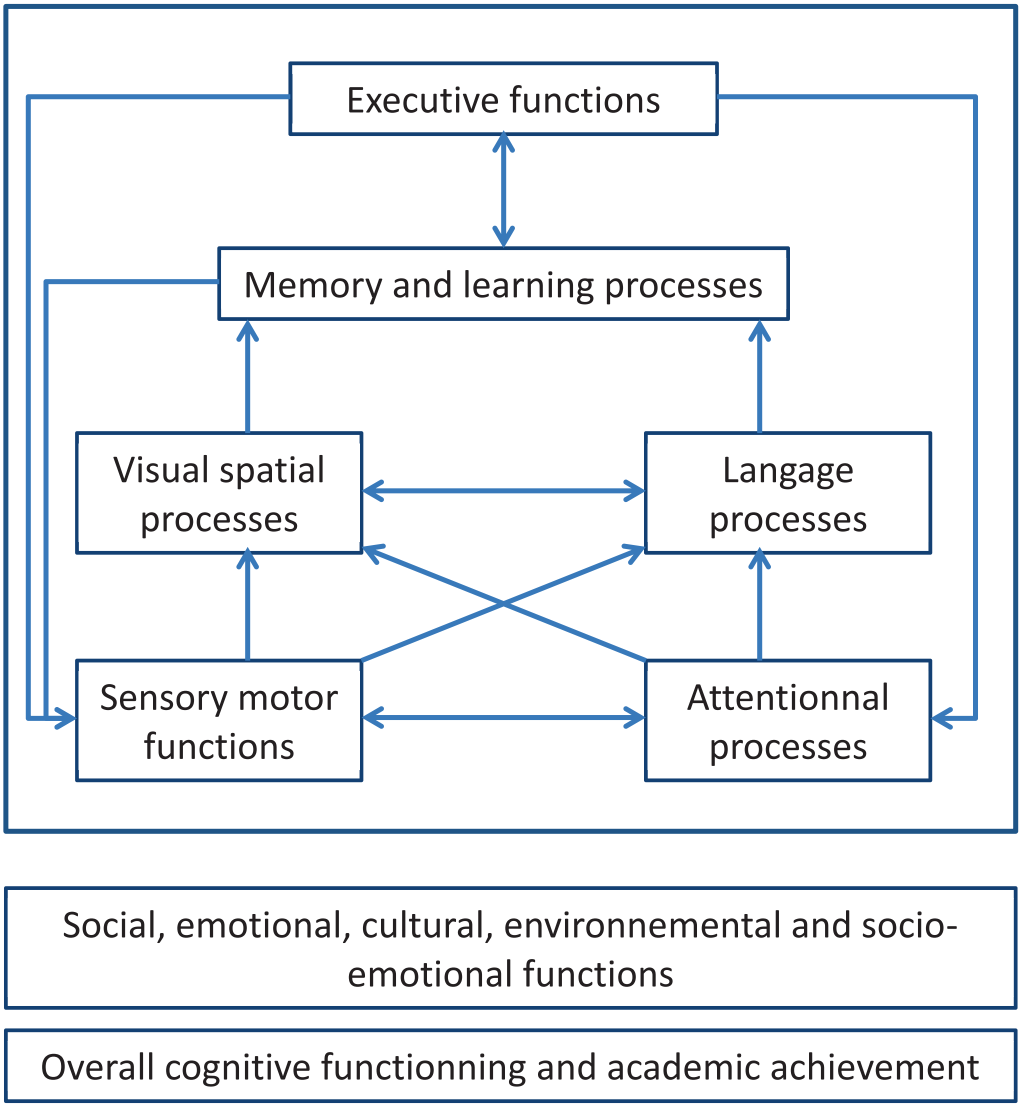

Appendix 1 – Conceptual model for scholl neuropsychological assessment

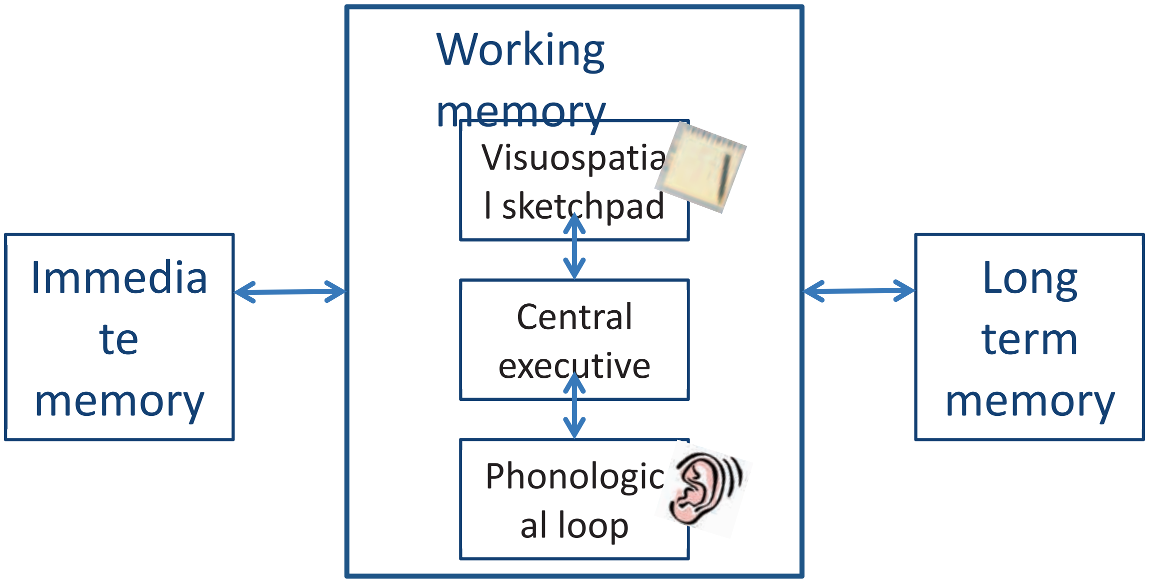

Appendix 2 – Working Memory model (Baddely and Hitch 1995)

Authors	Population	Participant characteristics	Preschool participants : Author, age years, CT, number	Diagnosis	Primary , secondary end points	Results
Kark D 2013	22 studies : children with various cognitive deficit	ADHD ,brain injury, specific learning disorder, healthy children, mental retardation, selective memory disorder, learning difficulties	Boivin MJ (6-16) (CT multi processes captain log) (RCT 32vs28)<br><br>Brown RT(5-13) (CT behavioral therapy) (RCT 19 vs14)<br><br>Butler RW (6-17) (CT metacognition)(RCT 109vs54)<br><br>Kozulin A (4-20)(CT at school : figural visuomotor modality (99vs49)<br><br>Braun (5-7)(CT behavioral therapy)(CS 14vs14)<br><br>Galbiati (6-18)(CT muliprocess: rehacom)(CS 40vs25)	Brain injury in connatal , hiv infection<br><br>ADHD<br><br>Brain injury (brain tumor)<br><br>Mental retardation<br><br>Brain injury<br><br>ADHD	Attention, EF,WM behavior<br><br>Attention, EF,WM, behavior, School Attention, school , ,self esteem<br><br>Attention, EF,WM, behavior, School Attention, school , ,self esteem<br><br>Intelligence<br><br>Attention, EF Intelligence<br><br>Attention EF behavior,Intelligence	Attention, EF,WM behavior : NS<br><br>***Wm,* FE*Behavior*,** School, attention, self esteem : NS<br><br>***Behavior** , Attention : NS<br><br>***Intelligence**<br><br>Attention: NS ***EF,*intelligence**<br><br>***Behavior, *Attention, *Intelligence,** EF : NS
Melby – lervag 2013	23 studies adults and children with CT of WM)(4-72 ans)	Learning difficulties, WM deficit, ADHD, older adult, dyslexia,mental defiiciencies	**Bergman Nutley , moy 4.3 (4-5 ans)(RCT CT WM cogmed)** (101)<br><br>Saint clair thompson 2008, (moy 4.5) (RCT CT : memory booster)<br><br>**Thorell 2009 (moy 4.8) (4-5 ans)RCT WM cogmed)(65)**	unselected preschool<br><br>unselected preschool<br><br>unselected preschool	Visuospatial WM, non verbal ability, problem solving, fluid intelligence<br><br>Verbal WM, visuo spatial WM, arithmetics<br><br>Verbal Wm,visual WM, non verbal ability, attention : 2 points	***WM visuo spatial** problem solving, fluid intelligence,: NS<br><br>***Verbal WM, *Visuo spatial WM**, arithmetic : NS<br><br>***Verbal Wm, *visual WM*,** non verbal ability,: ns , attention : 0.05
Kirk 2015	17 studies (children with WM CT , 10 with attention CT)	WM in TD children and ADHD. Attention training in TD and ADHD, CT in children with ID	**Bergman Nutley , moy 4.3 (4-5 ans)(RCT CT WM cogmed) (101)**<br><br>**Thorell 2009 (moy 4.8) (4-5 ans)RCT WM cogmed)(65)**<br><br>**Soderq vist 2012 (6-12) (RCT CT WM: cogmed)(41)**<br><br>Halperin 2012(5 ans) (CT teams : attention)(29)<br><br>Rudea(5 ans) (CT :attention)(37)	ADHD<br><br>ADHD<br><br>Unseslected preschool	Verbal WM,visual WM, short term memory, reaonning, parents ADHD symptom<br><br>Parent and teacher rating attention,ADHD impairment<br><br>FE	***Verbal Wm,*visual WM, *short term memory ,** reasonning parents adhd symptom : NS<br><br>Parent and teacher rating attention*, ADHD impairment : NS<br><br>***Fluid intelligence**,vocabulary : NS
Megan spencer smith 2015	CT WM In inattention	ADHD, WM impairment	Grunewald 2013 5-6 ans (CS WM CT: cogmed**)**	WM impairement (VLBW)	Inattentive attention, verbal et visuo-spatial WM	*** attention, *verbal et visuo-spatial WM**

TABLE 1 – CT in preschool children in the different metanalysis (Kark 2013, Melby Lervag 2013, Kirk 2015, Magan Spencer –Smith 2015)

**significance: p<0.05, NS : non significant, gras : preschool children studies.RCT: randomized controlled studies , CS: controlled studies, WM: working memory, EF: executive functions, TD: typically developing, ADHD : Attention Deficit Hyperactivity Disorder,VLBW: very low birth weight*

Long-term effects of visual spatial working memory training program performed at preschool age in very preterm infants with visual spatial working memory deficit
A Randomized Controlled Trial

EPIREMED

Statistical Analysis Plan

V1 1 2 2020

**Sponsor**	Assistance Publique des Hôpitaux de Marseille
**Grant**	PHRC-15-626
**Coordinator**	Catherine Gire<br>Department of Neonatology, North Hospital, APHM University Hospital
**Data manager**	Any Beltran
**Methodologist**	Julie Berbis
**Statistical analysis**	Mohamed Boucekine<br>Service d'Epidémiologie et Economie de la Santé, AP-HM
**N°eudract**	2016-A00122-49
**ClinicalTrials.gov ID**	NCT 02757794

Contents

I. Administrative details ... 3
II. Hypothesis and objectives (study protocol version n°4 - 26/10/2017) ... 4
1. Objectives ... 4
2.1. Primary objective and primary endpoint ... 4
2.2. Secondary objectives and secondary endpoints ... 4
III. Method ... 4
1. Design ... 4
2. Selection criteria ... 5
3.1. Inclusion criteria ... 5
3.2. Exclusion criteria (non-inclusion criteria) ... 5
3. Sample size ... 5
4. Data management ... 6
5. General ... 6
6. Flow chart ... 6
7. Description ... 6
8. Data imputation ... 6
9. Univariate analysis ... 7
10. Statistical modelling of the primary and secondary outcomes ... 7

I. Administrative details

Protocol

Main stages of the study	Date
Grant	2015
French Ethics Committee	April 12, 2016
Study start (enrolment of first inclusion)	November 29, 2016
Study completion (last follow-up)	August 20, 2019
Data manager	September 2019 – January 2020

SAP Versions

Number	Date	Justification
V1	1 2 2020	

Roles & responsibilities

Name	Function	Role
Catherine Gire	Principal investigation	Review and validation of the SAP
Julie Berbis	Analysis supervisor	Co-writer of SAP[1]
Mohamed Boucekine	Biostatistician	Writer of SAP

[1] Writing the SAP according to JAMA recommendations [Gamble C, Krishan A, Stocken D, et al. Guidelines for the Content of Statistical Analysis Plans in Clinical Trials. JAMA. 2017;318(23):2337–2343. doi:10.1001/jama.2017.18556]

II. Hypothesis and objectives (study protocol version n°4 - 26/10/2017)

1. Objectives

2.1. Primary objective and primary endpoint

The main objective is to assess the long-term effects (18 months) of CT (Cogmed) on visuospatial processing in preschool-age VP infants with WM impairment. Visuospatial processing is a broad cognitive process encompassing many subcomponents such as attention, sensory-motor skills, EF and visuospatial WM.

2.2. Secondary objectives and secondary endpoints

To assess, after 6 months of the intervention program, the effects of CT on the following parameters:

- Intellectual functioning: global IQ (Wechsler Pre-Primary Scale of Intelligence)
- Cognitive processing: language and visuospatial processing, working memory, fluidity of intelligence, and speed processing by five primary indexes of the Wechsler Pre-Primary Scale of Intelligence
- Executive functions: auditory attention, planification and inhibition
- Language processing and its abilities: ability of cultural and cognitive verbal learning, phonological judgment and semantics, verbal processing speed, verbal WM, motor programming, visual attention, analogizing ability
- Child behavior
- Parental anxiety
- Child quality of life
- School performance

III. Method

1. Design

This trial is a multicenter, randomized, controlled, open-label, two-parallel group study. The recruitment will be prospective.

The two groups are:

- A control group: standard care management
- An experimental group: standard care management in association with a 2-month Cognitive Training program called Cogmed JM

2. Selection criteria

3.1. Inclusion criteria

- Already included in the EPIPAGE 2, born between 24 and 34 weeks' GA (gestational amenorrhea),
- Children aged 5 to 6 years,
- Children exhibiting a total intellectual quotient >70 from the WPPSI IV (during the 5-year assessment in EPIPAGE 2),
- Children having a visuo-spatial Working Memory impairment defined by a working memory index <85 from the WPPSI IV,
- Children with parents (or legal guardians) authorizing participation in the study and a signed informed consent form,
- Children affiliated with medical insurance.

3.2. Exclusion criteria (non-inclusion criteria)

- Children with severe cerebral palsy, based on the Gross Motor Function (GMFCS score >2) and Bimanual Fine Motor Function (BFMF >2) classification system (Elvrum AG 2015, Marois P 2015),
- Children with blindness or amblyopia, defined by a visual acuity <3 (during the 5-year assessment in EPIPAGE 2),
- Children with deafness, as defined by a prescribed hearing aid,
- Children with chromosomal disorder or autistic syndrome,
- Children included in the EPILANG study protocol (an ancillary project to EPIPAGE),
- Children who do not speak French
- Children with parents having no internet connection,
- Triplets.

3. Sample size

A sample size of 64 participants per group (n=128 total) was selected to obtain an 80% power to detect a difference of 7.5 points on the VSI (0.5 standard deviation) at 16 months post training between the two groups and was clinically significant compared to similar studies.[1,2] The threshold for statistical significance had a p-value of .05. We assumed 166 patients were needed (83 per group) by projecting 23% of patients would be lost to follow-up between baseline and last assessments.

4. Data management

The monitoring of the study (including data management and data quality insurance) will be ensured by the sponsor. Quality assurance will be performed from the final database. The database will be forward to the statistical team (Clinical research unit, DRC, APHM).

5. General

Statistical analysis of this study will be carried out by Mohamed Boucekine under the responsibility of Pr Julie Berbis (Service d'Epidémiologie et Economie de la Santé, AP-HM). Analysis will be performed using SPSS software version 20.0. Statistical significance was defined as p<0.05. One interim analysis will be planned at 50% of the inclusions.
The methodology will be based on the Consolidated Standards of Reporting Trials Statement (CONSORT, http:// www.consort-statement.org/consort-statement/).

6. Flow chart

A chart will summarize the number of eligible subjects, the number of included subjects, the number of randomized subjects, the number of subjects who completed the follow-up at each evaluation times as well as the reasons of subjects lost to follow-up and excluded from analysis. The total number of subjects at different stages of the study will be presented using the following model (CONSORT 2010 Flow Diagram)

7. Description

The number of eligible subjects, the number of included subjects and the ratio will be provided. The number of inclusions per center will also be provided (total, per group).
First, a descriptive analysis of the whole sample will be performed (intention-to-treat population); characteristics collected at inclusion correspond to medical history, data related to admission and to inclusion.
Qualitative variables will be presented as proportions and numbers, quantitative variables as the means and standard deviation, or median and interquartiles. For each variable, the proportion of missing data will be specified.
Results will also be produced by group: "experimental" and "control". As recommended by the CONSORT group, comparison of the 2 groups on the variables collected at inclusion will not be provided for the publication. It will be provided in the final report.

8. Data imputation

Intention-to-treat (ITT) analyses were done. To fortify the validity of the results for the most accurate regression model estimates, a multiple imputation procedure was conducted before all analyses. For randomized clinical trials with missing data, the multiple imputation

procedure is a valid method to handle missing data[3] and to minimize possible biases.[4] To contextualize the impact of imputation, univariate and multivariate analyses without multiple imputations were also performed but not reported.

The imputation of multiple data sets was carried out using the chained equations algorithm.[5] This approach has the advantage to handle different types of variables (continuous, binary, categorical, ordinal) and does not assume multivariate normality of the data.[6] Five imputation datasets were used according to the literature.[7–9] In practice, chained equations algorithm involves running a series of regression models such that each variable with missing data is regressed on the other variables in the data set according to its distribution. For example, categorical variables will be modelled using logistic regression and continuous variables will be modelled using linear regression. More details of the process are provided in Hendry's article.[10]

The imputed variables were those of the main criterion and the secondary criteria, assessed in the three evaluations times as well as the adjustment variables (see "Statistical modelling of the primary and secondary outcomes section").

For the primary outcome the proportion of missing value were 0%, 11%, and 19% at each time point (baseline; at six-months [+/-2 months], and at 16-months [+/-2 months] after training) respectively. For the secondary outcomes and adjustment variables the proportion of missing values ranged from 0.6% to 22.5%.

9. Univariate analysis

A first univariate comparison of the primary outcome (visuospatial processing) and the secondary outcomes (WM, visuospatial processing, language skills, intellectual functioning, children's behavioral, executive and attention processes, quality of life and schooling) were carried out between the randomized Cogmed© and the control group using the t-test (or Mann-Whitney) for the quantitative variables and Chi square test (or Fisher's exact test) for the qualitative variables.

10. Statistical modelling of the primary and secondary outcomes

To assess the effect of CT on these outcomes at each time point (baseline; at six-months [+/-2 months], and at 16-months [+/-2 months] after training), a mixed model for repeated measures was performed for each outcome. We systematically adjusted for confounding by including the followed confounder in the mixed regression model: neurodevelopmental profile severity (for three subtests of NEPSY 2: (auditory attention score, design fluency score, inhibition score), one subtest of WPPSI IV (processing speed index), the presence of an impairment in motor performance assessed by the motor assessment of MABC-2 battery (scores of MABC-2 <=5th percentile), gestational age, birth weight, child's sex, parents' socio-professional status and parents' educational levels. Theses confounding factors were

selected by univariate preselection using a threshold p-value <=0.1or by their clinical relevance.

Estimated marginal means for each group at each time was reported. The unstructured covariance matrix was used for the within-subject correlation.

An additional sensitivity analysis using the previous mixed model was conducted for participants who completed at least 15 training sessions by Cogmed© recommendations to assess robustness of the conclusions.

When appropriate, 95% confidence intervals (CIs) were presented. The alpha level of significance was set at 5%, hence values where $p<.05$ values were referred to as a ‘‘statistically significant difference between the two groups”. According to Cohen’s criteria, the size difference between the two groups was measured by the effect-size[11].

Another sensitivity analysis was conducted for participants who completed at least 20 or 25 training sessions by Cogmed.

References

1. Anderson PJ. Neuropsychological outcomes of children born very preterm. *Semin Fetal Neonatal Med*. 2014;19(2):90-96. doi:10.1016/j.siny.2013.11.012
2. Pascoe L, Roberts G, Doyle LW, et al. Preventing academic difficulties in preterm children: a randomised controlled trial of an adaptive working memory entraînement intervention - IMPRINT study. *BMC Pediatr*. 2013;13:144. doi:10.1186/1471-2431-13-
3. Jakobsen JC, Gluud C, Wetterslev J, Winkel P. When and how should multiple imputation be used for handling missing data in randomised clinical trials - a practical guide with flowcharts. *BMC Med Res Methodol*. 2017;17(1):162. doi:10.1186/s12874-017-0442-1
4. Sterne JAC, White IR, Carlin JB, et al. Multiple imputation for missing data in epidemiological and clinical research: potential and pitfalls. *BMJ*. 2009;338:b2393. doi:10.1136/bmj.b2393
5. IBM Software. IBM SPSS Missing Values - Build better models when you fill in the blanks. Published June 2011. Accessed July 17, 2023. https://www.dmss.com.br/produtos/statistics/download/IBM%20SPSS%20Missing%20Values.pdf
6. Lee KJ, Carlin JB. Multiple imputation for missing data: fully conditional specification versus multivariate normal imputation. *Am J Epidemiol*. 2010;171(5):624-632. doi:10.1093/aje/kwp425

7. Austin PC, White IR, Lee DS, van Buuren S. Missing Data in Clinical Research: A Tutorial on Multiple Imputation. *Can J Cardiol*. 2021;37(9):1322-1331. doi:10.1016/j.cjca.2020.11.010

8. Buuren S van. *Flexible Imputation of Missing Data*. Chapman and Hall/CRC; 2012. doi:10.1201/b11826

9. Rubin DB. *Multiple Imputation for Nonresponse in Surveys*. Wiley; 1987.

10. Hendry GM, Naidoo RN, Zewotir T, North D, Mentz G. Model development including interactions with multiple imputed data. *BMC Med Res Methodol*. 2014;14:136. doi:10.1186/1471-2288-14-136

11. Cohen J. *Statistical Power Analysis for the Behavioral Sciences*. 2nd ed. L. Erlbaum Associates; 1988.
