# Supplementary material for: Cognitive Training for Visuospatial Processing in Children Aged 5½ to 6 Years Born Very Preterm With Working Memory Dysfunction: A Randomized Clinical Trial
Source: JAMA Netw Open. 2023 Sep 7;6(9):e2331988. doi: 10.1001/jamanetworkopen.2023.31988 (PMC10485729; doi:10.1001/jamanetworkopen.2023.31988)
Supplement: Supplement 2. — eTable 1. Collected Measures eReferences eTable 2. Demographics and Clinical Characteristics of the Included and Nonincluded Children at Birth and at 5 Years Old eTable 3. Description of Training Adherence in the Intervention Group eTable 4. Training Adherence in the Intervention Group According to Parents’ Socioprofessional Status and Educational Level With Missing Data Imputation eTable 5. Sensitivity Analysis Adjusted With and Without Missing Data Imputations Restricted to Children Who Completed at Least 20 or 25 sessions of Training in the Intervention Group eTable 6. Missing Data Distribution [file jamanetwopen-e2331988-s002.pdf]

Supplemental Online Content

Gire C, Beltran Anzola A, Marret S, et al; EPIREMED Study Group. Cognitive training for visuospatial processing in children aged 5½ to 6 years born very preterm with working memory dysfunction: a randomized clinical trial. *JAMA Netw Open*. 2023;6(9):e2331988. doi:10.1001/jamanetworkopen.2023.31988

eTable 1. Collected Measures

eReferences

eTable 2. Demographics and Clinical Characteristics of the Included and Nonincluded Children at Birth and at 5 Years Old

eTable 3. Description of Training Adherence in the Intervention Group

eTable 4. Training Adherence in the Intervention Group According to Parents' Socioprofessional Status and Educational Level With Missing Data Imputation

eTable 5. Sensitivity Analysis Adjusted With and Without Missing Data Imputations Restricted to Children Who Completed at Least 20 or 25 sessions of Training in the Intervention Group

eTable 6. Missing Data Distribution

This supplemental material has been provided by the authors to give readers additional information about their work.

© 2023 Gire C et al. *JAMA Network Open.*

eTable 1. Collected Measures

Domains	Measures	Population assessed	Description	Baseline (Epipage 2 database)	Inclusion visit	Intermediate visit	Final visit
Clinical and sociodemographic data at birth		Mother and child					
Maternal and obstetrical factors	Maternal age			x			
	Parents' socio-professional status			x			
	Antenatal corticosteroid therapy with a full-course minimum			x			
	Antenatal administration of magnesium sulfate during the last hospitalization			x			
	Multiple pregnancies			x			
	Gestational age			x			
	Birth weight			x			
	Sex of child			x			
	Mode of delivery			x			
Neonatal and postnatal period	Apgar at 5 minutes	Child		x			
	Bronchopulmonary dysplasia			x			
	Postnatal corticotherapy			x			
	Presence of IVH			x			
	Necrotizing enterocolitis			x			
	Severe neonatal morbidity			x			
	Late onset sepsis			x			
	Presence of severe lesions			x			
Sociodemographic data at five years							
Family data	Maternal educational level				x		
	Paternal educational level				x		
	Siblings				x		
	Living situation (Housing situation to describe their environment)				x		
Primary Outcomes							
Visual-spatial processing	Visual-Spatial Index (VSI)	Child	FSIQ Subtest – WPPSI-IV (Means: 100 ; CI:15)	x		x	x
Secondary Outcomes							

© 2023 Gire C et al. *JAMA Network Open.*

Working memory	Working Memory Index (WMI)	Child	FSIQ Subtest - WPPSI-IV (Means: 100 ; CI:15)	x		x	x
Motor performance	Manual Dexterity, Aiming, Catching, Balance	Child	MABC-2 Subtests	x			
Attention and Executive Functioning	Statue	Child	NEPSY-2 Subtest (Means:10; CI:3)	x		x	
	Speeded Naming			x		x	
	Design Fluency				x	x	
	Inhibition			x			
	Auditory attention				x	x	x
Intellectual functioning	Full Scale Intellectual Quotient (FSIQ)	Child	WPPSI-IV (Means: 100; CI:15)	x		x	
	Verbal Comprehension Index (VCI)			x		x	
	Fluid Reasoning Index (FRI)			x		x	
	Processing Speed Index (PSI)			x		x	
Language skills*	Lexicon	Child	CléA Subtest (Means: 100; CI:15)		x	x	
	Phonology				x	x	
	Morphosyntax				x	x	
	Resources				x	x	
	Comprehension				x	x	
	Production				x	x	
	Judgment				x	x	
Parental assessment of child’s behavior	Emotional Symptoms	Parents	Goodman Strengths and Difficulties Questionnaire		x	x	
	Conduct Problems				x	x	
	Hyperactivity				x	x	
	Peer Problems				x	x	
	Prosocial Behavior				x	x	
	Total Deviance/Difficulties				x	x	
Parental assessment of child’s Quality of life	Relationship with parents/family	Parents	VSP-A Dimension		x	x	
	School performance		VSP-A Dimension		x	x	
	Relationship with teacher		VSP-A Dimension		x	x	
Educational assistance	Assistance at school	Parents	Special needs teaching assistant (SNA)/assistance by a professional other than an SNA/technical aid/personalized education plans for children with disabilities other types of support at school		x		

© 2023 Gire C et al. *JAMA Network Open.*

Allied health intervention	Medical consultation	Parents	Follow-up by a specialist center (Early Medial-Social Action Centers and Medical Psychologic Centers of Early Childhood) or by a professional (a psychologist/a psychometrician/an orthoptist/a speech therapist/a physiotherapist)		x	x
Parents' anxiety	Anxious state score	Parents	State-Trait Anxiety Inventory (STAI)	x	x	
	Anxious trait score			x	x	
Schooling of child assessed by a teacher	Five Verbal skills, five nonverbal skills, class behavior	Teacher	GSA questionnaire	x	x	

Abbreviations: FSIQ = Full-Scale Intelligence Quotient; WPPSI-IV=Wechsler Preschool and Primary Scale of Intelligence, Fourth Edition; MABC-2=The Movement Assessment Battery for Children-Second Edition; NEPSY-2=NEuroPSYchology assessment - Second Edition; VSP-A=Perceived Quality of Life and Health of Adolescents and Children Questionnaire; GSA=Global School Adaptation score.

Notes[1]: ***Primary endpoint*:** The visual-spatial processing will be assessed by using the visuospatial index (VSI) of the WPPSI-IV at the inclusion visit, the six-month post-intervention (+/− 2 months) and at 18-months post-intervention (+/− 2 months). This index consists of two subtests: block design and object assembly. The average score is 100 with a standard deviation of 15. Rationale for the primary endpoint: The visual perceptual integration testing performance for VPs is poorer than those born full term, and these VPs present with a visuo-constructive dyspraxia. This deficit is connected to a poor integration of visual function, perceptual and/or fine motor skills (e.g., reproduction of a complex geometric figure). This disorder is three times more frequent in VP adolescents than in those born at term. Among those born extremely prematurely, 30% have results below the 15th percentile in visuospatial performance. The impact of WM rehabilitation on visuospatial skills is an interesting line of research, most particularly in premature infants. ***Secondary endpoints:*** The secondary endpoints, related to the children and to their parents, will be assessed at the inclusion visit and at the six-month post-intervention (+/− 2 months). Children's endpoints: The intellectual functioning and other cognitive processes will be obtained by global intellectual quotient (IQ) and IQ indices using the WPPSI-IV (Wechsler). The WPPSI-IV, designed for children ages 4-to-7-years-old, assesses the overall intellectual functioning (total comprehensive intellectual quotient) and specific cognitive processes. This is done through the main indices: verbal comprehension index, fluid reasoning index, visual spatial processing speed index, and the working memory index. The evaluation of the Working Memory by the Working Memory Index (WMI) in WPPSI-IV is uniquely visuospatial at this age. The average is 100 with a standard deviation of 15, as with all Wechsler Scales. The global FSIQ, as well as the main indices, will be assessed when monitoring the EPIPAGE cohort. It is lower for VP infants as compared to full-term infants. Executive and attention processes: The NEPSY-2 (NEuroPSYchology assessment - Second Edition) assesses the neuropsychological development of preschool and school age children (3–12 years old) and is used to obtain emerging executive functions in 5–6-year-olds. Auditory attention, statue, and design fluency are the only three subtests in the Executive and Attention Function domains which will be administered in this study. These three tests measure selective and divided attention in auditory modality, inhibition and Speeded Naming. A review of Van de Weijer-Bergsma,[2] which is confirmed by Mulder in a meta-analysis[3], shows that the selective, divided and supported attentional domains are likely to be affected in VP preschoolers. Evaluation of language and its skills: Language is a complex mental process requiring an assessment of all its components. This assessment will be made from the CléA battery calibrated for those children between 2- and 15-years-old.[4] The battery consists of seven tests: lexicon: Known Digital Channels: a reflection of verbal learning ability, both cultural and cognitive; phonology: Oral Word Identification: capacity to give a word phonological and semantic judgment, morphosyntax: Rapid Denomination: timed tested; gives an indication treatment speed; resources: Word memory: explores the short-term mnemonic span; **comprehension:** Facial and oral praxis photographs: relevant in motor programming; **production**: Visual attention: inspired from NEPSY, questions the visual spatial component; **judgment:** Resolution of logical problems: ability to reason analogically (progressive matrices). Studies suggest that specific language disorders can be associated with specific WM impairments, particularly with the phonological loop.[5,6] Two meta analyses**-**studies demonstrate global language gaps/impairment in the very premature vs. the full-term child with deficits in learning ability, phonology, semantics, grammar, speech coherence and verbal reasoning.[7,8] Behavioral Evaluation: the child's behavior will be assessed with the Goodman Strengths and Difficulties Questionnaire, which includes 25 self-administered questions answered by the parents, and will assess any impact this intervention has on the child's behavior.[9] Evaluation of the child's quality of life (QoL): The quality of life of the children will be assessed using the Perceived Quality of Life and Health of Adolescents and Children Questionnaire (VSP-A [*Vie et Santé Perçue de l'Adolescent et de l'enfant*]) as reported by the parents.[10] The 49-item version portrays nine dimensions and index: relationships with parents/family, body image, vitality, relationships with friends, general well-being, leisure, school performance, relationships with teacher, and relationships with medical staff. A higher score, which ranges between 0 and 100, indicates a better QoL. The French norms are available through Ravens-Sieberer U 2007.[11] Schooling: This will be evaluated by the GSA questionnaire (Global School Adaptation score), a French tool completed and validated by the teacher,[12] and re-evaluated in a preschool population in 2013.[13] The questionnaire covers five verbal skills (verbal communication, verbal participation, vocabulary, syntax, pronunciation), five non-verbal abilities (memory, arithmetic, logical reasoning skills, manual dexterity and fine motor skills) and eight questions evaluating class behavior (compliance with rules, attention, autonomy, speed of accomplishing the task, self-esteem, ability to keep the pace and fatigability). The final question asks the teacher about possible future special educational needs of the child. Parents' endpoint: Anxiety: The Spielberger state-trait anxiety inventory (STAI) will be used to assess anxiety. The STAI is a self-reporting questionnaire consisting

© 2023 Gire C et al. *JAMA Network Open.*

of 40 items that measure both the state and trait scores. These scores range from 20 (absence of anxiety) to 80 (high anxiety). This questionnaire will assess if anxiety is impacted as a result of parental intervention (mother).[14] Rationale for the secondary endpoints: It is of value to measure the impact of intervention on the WM and on other non-trained brain processes as well as on parental anxiety, child behavior and parental perceptions of the child's quality of life.

References:

1. Gire C, Beltran Anzola A, Kaminski M, et al. A randomized EPIREMED protocol study on the long-term visuo spatial effects of very preterm children with a working memory deficit. *BMC Pediatr*. 2021;21(1). doi:10.1186/s12887-021-02867-x
2. van de Weijer-Bergsma E, Wijnroks L, Jongmans MJ. Attention development in infants and preschool children born preterm: a review. *Infant Behav Dev*. 2008;31(3):333-351. doi:10.1016/j.infbeh.2007.12.003
3. Mulder H, Pitchford NJ, Hagger MS, Marlow N. Development of executive function and attention in preterm children: a systematic review. *Dev Neuropsychol*. 2009;34(4):393-421. doi:10.1080/87565640902964524
4. Charollais A, Stumpf MH, De Quelen R, et al. Delayed language development at two years of age in very preterm infants in the Perinatal Network of Haute-Normandie. *Early Hum Dev*. 2014;90(12):891-892. doi:10.1016/j.earlhumdev.2014.10.001
5. Adams AM, Gathercole SE. Limitations in working memory: implications for language development. *Int J Lang Commun Disord*. 2000;35(1):95-116.
6. Rodrigues A, Befi-Lopes DM. Phonological working memory and its relationship with language development in children. *-Fono Rev Atualizacao Cient*. 2009;21(1):63-68.
7. van Noort-van der Spek IL, Franken MCJP, Weisglas-Kuperus N. Language functions in preterm-born children: a systematic review and meta-analysis. *Pediatrics*. 2012;129(4):745-754. doi:10.1542/peds.2011-1728
8. Reidy N, Morgan A, Thompson DK, Inder TE, Doyle LW, Anderson PJ. Impaired language abilities and white matter abnormalities in children born very preterm and/or very low birth weight. *J Pediatr*. 2013;162(4):719-724. doi:10.1016/j.jpeds.2012.10.017
9. Goodman R. The Strengths and Difficulties Questionnaire: a research note. *J Child Psychol Psychiatry*. 1997;38(5):581-586.
10. Auquier P, Clement A, Sapin C, Khammar ME, Marco JLS, Simeoni MC. Development, Validation of HRQL Measurement in Children: VSP-AE. *Qual Life Res*. 2001;10(3):268-268.
11. Ravens-Sieberer U. *The Kidscreen Questionnaires: Quality of Life Questionnaires for Children and Adolescents ; Handbook*. Pabst Science Publ.; 2006.
12. Guimard P, Cosnefroy O, Florin A. Évaluation des comportements et des compétences scolaires par les enseignants et prédiction des performances et des parcours à l'école élémentaire et au collège. *Orientat Sc Prof*. 2007;(36/2):179-202. doi:10.4000/osp.1372
13. Boussicault G, Nguyen The Tich S, Branger B, et al. The Global School Adaptation score: a new neurodevelopmental assessment tool for very preterm children at five years of age. *J Pediatr*. 2013;163(2):460-464. doi:10.1016/j.jpeds.2013.01.052
14. Spielberger CD. Manual for the State-Trait Anxiety Inventory STAI (Form Y) ("Self-Evaluation Questionnaire"). Published online 1983. Accessed April 8, 2021. http://ubir.buffalo.edu/xmlui/handle/10477/1873

© 2023 Gire C et al. *JAMA Network Open.*

eTable 2. Demographics and Clinical Characteristics of the Included and Nonincluded Children at Birth and at 5 Years Old

Characteristic	Total missing data. N	Eligible children (N=313)[a]		p-value
		Non-Participant (n=144)	Participant (n=169)	
At birth				
Maternal age. mean (SD)	0	30 y 4 mo (5 y 9 mo)	30 y 2 mo (6 y 3 mo)	.76
Parents' socioprofessional status[b]	20			.34
Higher		95/134 (71)	124/159 (78)	
Lower		37/134 (28)	33/159 (21)	
Without socioprofessional status		2/134 (1)	2/159 (1)	
Antenatal corticosteroid therapy with a full-course minimum	21	96/135 (71)	102/157 (65)	.26
Rescue course of antenatal corticosteroid therapy	11	3/139 (2)	3/163 (2)	>.99
Antenatal administration of magnesium sulfate during last hospitalization	12	15/137 (11)	22/164 (13)	.52
Multiple births	0	50/144 (35)	47/169 (28)	.19
If two fetuses. chorionicity	17			.10
Dichorionic/diamniotic		29/41 (71)	34/39 (87)	
Monochorionic/monoamniotic		3/41 (7)	0 (0)	
Monochorionic/diamniotic		9/41 (22)	5/39 (13)	
Gestational age. mean (SD), wk	0	29.6 (2.5)	29.6 (2.6)	.90
Birth weight. mean (SD), g	2	1294.5 (472.4)	1317.3 (506.0)	.68
Birth weight, percentile of EPOPé curves	2			.47
<10th		67/142 (47)	74/169 (44)	
≥ 10th		75/142 (53)	95/169 (56)	
Child's sex	0			.40
Female		73/144 (51)	78/169 (46)	
Male		71/144 (49)	91/169 (54)	
Mode of delivery	6			.45
Vaginal without instruments		42/141 (30)	40/166 (24)	
Vaginal with instruments		3/141 (2)	6/166 (4)	
Cesarean		96/141 (68)	120/166 (72)	
Apgar at 5 minutes. mean (SD)	19	8.7 (1.9)	8.3 (2.0)	.07
Bronchopulmonary dysplasia[c]	13			.81
None		98/137 (72)	122/163 (75)	
Mild		20/137 (15)	20/163 (12)	
Moderate		2/137 (1)	4/163 (2)	
Severe		17/137 (12)	17/163 (10)	
Postnatal corticotherapy	11	15/139 (11)	18/163 (11)	.94
Intraventricular hemorrhage	5			.97
None		104/141 (74)	121/167 (72)	
Grade 1 or Grade 2		32/141 (23)	40/167 (24)	
Grade 3 or Grade 4		5/141 (4)	6/167 (4)	
Necrotizing enterocolitis	5	5/142 (4)	12/166 (7)	.16
Severe neonatal morbidity	11	29/138 (21)	32/164 (20)	.75
Late-onset sepsis	3	31/143 (22)	34/167 (20)	
Presence of severe lesions on last TFU scan before discharge	5	9/141 (6	7/167 (4)	.39
At five years old				
Child's age. mean (SD)	1	5 y 7 mo (2 mo)	5 y 7 mo (2 mo)	.06
Visual-spatial index				
Score. mean (SD)	1	91.9 (12.0)	93.8 (11.5)	.14
Working memory index				
Score. mean (SD)	1	81.3 (7.2)	80.00 (5.3)	.06

© 2023 Gire C et al. *JAMA Network Open.*

Total MABC-2 score. mean (SD)	7	76.7 (13.4)	74.5 (14.3)	.17
Intellectual functioning				
Full scale IQ (FSIQ). Z-score. mean (SD)	1	88.2 (10.1)	88.5 (10.3)	.83
Verbal comprehension index. Score. mean (SD)	1	94.7 (13.5)	96.1 (13.4)	.35
Fluid reasoning index. Score. mean (SD)	1	92.4 (10.9)	94.0 (12.3)	.24
Processing speed index. Score. mean (SD)	1	92.2 (12.3)	90.6 (12.3)	.26
Executive and attention processes				
Inhibition. Score. mean (SD)	70	8.7 (3.0)	7.9 (3.1)	**.05**
Statue. Score. mean (SD)	30	9.6 (3.5)	9.3 (3.3)	.42
Speeded Naming. Score. mean (SD)	115	10.0 (3.1)	9.7 (2.9)	.41
Educational assistance at school[d]	37	21/127 (17)	32/149 (21)	.30
Medical consultation[f]	37	68/128 (53)	88/148 (59)	.29

Abbreviations: EPOPé=Obstetrical, Perinatal and Pediatric Epidemiology Research Team of the Center for Research on Epidemiology and Statistics Sorbonne Paris Cité (CRESS) at the National Institute for Health and Medical Research (INSERM) and the Paris-Descartes University, CPAP=Continuous Positive Airway Pressure, FIO_2=Fraction of inspired oxygen, IVH=Intraventricular hemorrhage, MABC-2=Movement Assessment Battery for Children - Second Edition, TFU= transfontanellar ultrasonography.

Note:

[a]Data are presented as the number of participants with data/total number (percentage) unless otherwise indicated.

[b]Higher socio-professional status comprises three working categories: managerial, intermediate, and administrator. Lower socio-professional status comprise two working categories: domestic or sales employee.

[c]Mild BPD was defined as 28 or more days of oxygen and spontaneous respiration-room air at 36 weeks. Moderate BPD was defined as as 28 or more days of oxygen and mechanical ventilation or continuous positive airway pressure or fraction of inspired oxygen greater than 21% at 36 weeks. Severe BPD was defined as 28 or more days of oxygen and mechanical ventilation or continuous positive airway pressure or fraction of inspired oxygen greater than 21% at 36 weeks.

[d]Children who benefitted from any the following school assistance: special needs teaching assistant, assistance by any other professional, technical aid, personalized educational plans for children with disabilities, and other types of support.

[f]Children who in the 12 months before inclusion in the study, were followed up at a specialist center (Early Medical-Social Action Centers and Medical Psychologic Centers of Early Childhood) or benefited from 1 or more medical consultations by the following professionals: psychologist, psychometrician, orthoptist, speech therapist, and/or physiotherapist.

© 2023 Gire C et al. *JAMA Network Open.*

eTable 3. Description of Training Adherence in the Intervention Group: All Participants Randomized in Intervention Group and Participants Who Completed at least 15, 20, and 25 Training Sessions

	Participants randomized in Intervention group		Participants who completed at least 15 training sessions		Participants who completed at least 20 training sessions		Participants who completed at least 25 training sessions	
	Participants, No.	Mean (SD)	Participants, No.	Mean (SD)	Participants, No.	Mean (SD)	Participants, No.	Mean (SD)
Number of sessions	84	18.1 (9.1)	57	23.8 (2.5)	52	24.4 (1.4)	43	25.00
Starting index[a]	84	41.4 (17.0)	57	47.5 (6.1)	52	46.8 (5.9)	43	45.9 (5.60)
Maximum index[b]	84	59.5 (24.4)	57	70.8 (6.9)	52	70.6 (7.00)	43	70.00 (6.9)
Progress Index[c]	84	18.2 (10.3)	57	23.4 (6.4)	52	23.8 (6.3)	43	24.0 (6.3)

Note:

[a] Calculated from the results obtained on the second and third working days, and the maximum index

[b] Calculated from the results obtained during the two best days of the training

[c] Calculated by subtracting the "Starting index" from the "Maximum index". The average expected progress index is around 24 units, but the distribution is wide and an average rate of progress is between 14 and 32 units, Higher Index scores indicate good compliance and effort with the training.

© 2023 Gire C et al. *JAMA Network Open.*

eTable 4. Training Adherence in the Intervention Group According to Parents' Socioprofessional Status and Educational Level With Missing Data Imputation: All Participants Randomized in Intervention Group and Participants Who Completed at least 15, 20, and 25 Training Sessions

Participants randomized in Intervention group	Participants who completed at least 15 training sessions		Participants who completed at least 20 training sessions		Participants who completed at least 25 training sessions	
	OR (95%CI)	P value	OR (95%CI)	P value	OR (95%CI)	P value
Higher parents' socio-professional status[a]. yes. n (%)	2.2 (0.7-6.5)	.15	2.7 (0.9-8.0)	.08	3.1 (1.0-9.3)	.05
Parents' educational level[b]. yes, n (%)	1.3 (0.5-3.7)	.57	1.5 (0.5-3.9	.45	1.2 (0.5-3.1)	.71

Abbreviations: OR = Odds Ratio; CIs=Confidence Intervals

Note:
[a] Higher socio-professional status comprise three working categories: managerial, intermediate, and administrator.
[b] At least one parent with an academics degree of least a first-year university level

© 2023 Gire C et al. *JAMA Network Open.*

eTable 5. Sensitivity Analysis Adjusted With and Without Missing Data Imputations Restricted to Children Who Completed at least 20 or 25 Sessions of Training in the Intervention Group

			Inclusion visit		Intermediate visit		Final visit	
		**Participants, No.**	**Group difference (95% CI)[a]**	**P value[b]**	**Group difference (95% CI)[a]**	**P value[b]**	**Group difference (95% CI)[a]**	**P value[b]**
Participants who completed at least 20 sessions of training in Intervention group								
Without missing data imputations	Visual-spatial index. Score. mean (SD)	91	0.6 (-4.3 ; 5.5)	.81	-1.0 (-6.0 ; 3.9)	.68	-3.5 (-9.5 ; 2.6)	.26
	Working memory index. Score. mean (SD)	91	2.0 (-0.5 ; 4.6)	.11	4.8 (0.7 ; 8.9)	**.02**	2.3 (-2.2 ; 6.9)	.31
With missing data imputations	Visual-spatial index. Score. mean (SD)	137	1.2 (-2.8 ; 5.3)	.56	-0.1 (-4.4 ; 4.1)	.96	-0.2 (-6.8 ; 6.4)	.96
	Working memory index. Score. mean (SD)	137	0.9 (-1.1 ; 3.0)	.37	5.9 (2.3 ; 9.4)	**.001**	3.1 (-1.9 ; 8.1)	.22
Participants who completed at least 25 sessions of training in Intervention group								
Without missing data imputations	Visual-spatial index. Score. mean (SD)	84	-0.4 (-5.7 ; 4.9)	.88	-0.9 (-6.2 ; 4.5)	.74	-1.5 (-7.9 ; 4.9)	.64
	Working memory index. Score. mean (SD)	84	1.7 (-1.0 ; 4.4)	.21	4.9 (0.5 ; 9.3)	**.03**	1.3 (-3.2 ; 5.7)	.57
With missing data imputations	Visual-spatial index. Score. mean (SD)	128	0.4 (-3.9 ; 4.7)	.86	-0.03 (-4.6 ; 4.5)	.99	0.5 (-5.7 ; 6.7)	.88
	Working memory index. Score. mean (SD)	128	0.8 (-1.4 ; 2.9)	.49	5.8 (2.1 ; 9.5)	**.002**	1.9 (-3.1 ; 6.9)	.46

Abbreviations: CIs=Confidence Intervals
Note:
[a] A group difference greater than 0 reflects a higher score in the intervention group, and less than 0 reflects a lower score
[b] Results are adjusted for neurodevelopmental profile severity for 3 subtests of NEPSY, 2nd Edition: auditory attention score, design fluency score, and inhibition score; 1 subtest of the Wechsler Preschool and Primary Scale of Intelligence, 4th Edition (processing speed index); the presence of an impairment in motor performance, assessed by the motor assessment of Movement Assessment Battery for Children, 2nd Edition scores in the 5th percentile or lower); and gestational age, birth weight, child’s sex, parents' socioprofessional status, and parents’ educational level.

© 2023 Gire C et al. *JAMA Network Open.*

eTable 6. Missing Data Distribution

**Missing data distribution**	Valid	Missing	% missing
Final visit - Working Memory Index	131	38	22.5
Final visit - Visual-Spatial Index	136	33	19.5
Baseline (Epipage 2 database) - Inhibition	136	33	19.5
Intermediate visit - Visual-Spatial Index	150	19	11.2
Intermediate visit - Working Memory Index	150	19	11.2
Inclusion visit - Parents' educational level	157	12	7.1
Baseline (Epipage 2 database) - Parents' socio-professional status	159	10	5.9
Baseline (Epipage 2 database) - Manual Dexterity, Aiming, Catching, Balance	163	6	3.6
Inclusion visit - Auditory attention	166	3	1.8
Inclusion visit - Design Fluency	166	3	1.8
Baseline (Epipage 2 database) - Birth weight	169	0	0.0
Baseline (Epipage 2 database) - Visual-Spatial Index	169	0	0.0
Baseline (Epipage 2 database) - Working Memory Index	169	0	0.0
Baseline (Epipage 2 database) - Sex of child	169	0	0.0
Baseline (Epipage 2 database) - Processing Speed Index	169	0	0.0
Baseline (Epipage 2 database) - Gestational age	169	0	0.0

© 2023 Gire C et al. *JAMA Network Open.*
