# Supplementary material for: Cognitive Training for Visuospatial Processing in Children Aged 5½ to 6 Years Born Very Preterm With Working Memory Dysfunction: A Randomized Clinical Trial
Source: JAMA Netw Open. 2023 Sep 7;6(9):e2331988. doi: 10.1001/jamanetworkopen.2023.31988 (PMC10485729; doi:10.1001/jamanetworkopen.2023.31988)
Supplement: Supplement 3. — EPIREMED Study Group [file jamanetwopen-e2331988-s003.pdf]

Supplemental Online Content: Nonauthor Collaborators
*First name, last name, and suffix (if applicable) are required and will appear in PubMed.

***Group Name(s): EPIREMED Study Group**

***First Name and Middle Initial(s)**	***Last Name**	***Suffix (eg, Jr, III)**	Academic Degrees	Institution	Location (city, state/province, country)	Role or Contribution, eg, chair, principal investigator	Group (if more than 1 Group listed in the byline) and/or Subgroup (eg, Steering Committee)
Barthélémy	TOSELLO			AP-HM, France	Marseille, France	Investigator	
Veronique	BREVAUT			AP-HM, France	Marseille, France	Investigator	
Patricia	GARCIA			AP-HM, France	Marseille, France	Investigator	
Tristan	DESILES			AP-HM, France	Marseille, France	Neuropsychologist	
Gwenaëlle	MENARD			AP-HM, France	Marseille, France	Neuropsychologist	
Mélodie	PACHE			AP-HM, France	Marseille, France	Neuropsychologist	
Ludovic-Mohamed	ZAHED			AP-HM, France	Marseille, France	Neuropsychologist	
Delphine	MITANCHEZ			AP- HP - Armand Trousseau Hospital,	Paris, France	Investigator	
Charlotte	COUDRONNIERE			University Hospital of Nantes, France	Nantes, France	Neuropsychologist	
Mireille	DENAVEAUT BOULAY			University Hospital of Caen Normand	Caen, France	Neuropsychologist	
Elodie	FALQUE			University Hospital of Nîmes, France	Nîmes, France	Neuropsychologist	
Magali	REBATTEL			University Hospital of Nîmes, France	Nîmes, France	Neuropsychologist	
Karine	VOIRIN			University Hospital of Reims, France	Reims, France	Neuropsychologist	
Eliane	BASSON			University Hospital of Lyon, France	Lyon, France	Investigator	
Sophie	RUBIO GURUNG			University Hospital of Lyon, France	Lyon, France	Investigator	
Mélanie	RODRIGUEZ			University Hospital of Lyon, France	Lyon, France	Neuropsychologist	
Hélène	DEFORGE			Regional University Hospital of Nancy	Nancy, France	Neuropsychologist	
Marie	LEMARCHAND			University Hospital of Rouen, France	Rouen, France	Neuropsychologist	
Emeline	DUBOIS			University Hospital of Toulouse, Fran	Toulouse, France	Neuropsychologist	
Stéphanie	IANNUZZI			University Hospital of Toulouse, Fran	Toulouse, France	Neuropsychologist	
Julie	OERTEL			University Hospital of Nice, France	Nice, France	Investigator	
Anne	DE SAINT MARTIN			Regional University Hospital of Strasb	Strasbourg, France	Investigator	
Claire	ZORES KOENIG			Regional University Hospital of Strasb	Strasbourg, France	Investigator	
Hélène	MUSMEAUX			Regional University Hospital of Strasb	Strasbourg, France	Neuropsychologist	
Lucille	SCHNEIDER			Regional University Hospital of Strasb	Strasbourg, France	Neuropsychologist	
Claire	LERAT			University Hospital of Montpellier, Fr	Montpellier, Frace	Neuropsychologist	
Angélique	PANNETIER			University Hospital of Clermont-Ferra	Clermont-Ferrand, France	Neuropsychologist	
Isabelle	PIN			University Hospital of Grenoble, Fran	Grenoble, France	Investigator	

Supplemental Online Content: Nonauthor Collaborators
*First name, last name, and suffix (if applicable) are required and will appear in PubMed.

*First Name and Middle Initial(s)	*Last Name	*Suffix (eg, Jr, III)	Academic Degrees	Institution	Location (city, state/province, country)	Role or Contribution, eg, chair, principal investigator	Group (if more than 1 Group listed in the byline) and/or Subgroup (eg, Steering Committee)
Thierry	DEBILLON			University Hospital of Grenoble, Fran	Grenoble, France	Investigator	
Karine	GUICHARDET			University Hospital of Grenoble, Fran	Grenoble, France	Neuropsychologist	
Patrick	ZANDER			University Hospital of Tour, France	Tour, France	Neuropsychologist	
Elie	SALIBA			University Hospital of Tour, France	Tour, France	Investigator	
Valérie	BENHAMMOU			University of Paris, CRESS, Obstetrica	Paris, France	Research Assistant	
Monique	KAMINSKI			University of Paris, CRESS, Obstetrica	Paris, France	Methodologist	
Laetitia	MARCHAND-MARTIN			University of Paris, CRESS, Obstetrica	Paris, France	Statistician	
Samira	MEDJAHED			University of Paris, CRESS, Obstetrica	Paris, France	Research Assistant	
Johanne	PIRRELLO			AP-HM, France	Marseille, France	Research Assistant	
Julie	BERBIS			CEReSS - Health Service Research anc	Marseille, France	Methodologist	
Pascal	AUQUIER			CEReSS - Health Service Research anc	Marseille, France	Methodologist	
Karine	BAUMSTARCK			CEReSS - Health Service Research anc	Marseille, France	Methodologist	
