# Supplementary material for: Cognitive Training for Visuospatial Processing in Children Aged 5½ to 6 Years Born Very Preterm With Working Memory Dysfunction: A Randomized Clinical Trial
Source: JAMA Netw Open. 2023 Sep 7;6(9):e2331988. doi: 10.1001/jamanetworkopen.2023.31988 (PMC10485729; doi:10.1001/jamanetworkopen.2023.31988)
Supplement: Supplement 4. — Data Sharing Statement [file jamanetwopen-e2331988-s004.pdf]

Data Sharing Statement

Gire. Cognitive Training for Visuospatial Processing in Children Aged 5½ to 6 Years Born Very Preterm With Working Memory Dysfunction. *JAMA Netw Open*. Published September 07, 2023. doi:10.1001/jamanetworkopen.2023.31988

Data All the individual participant data after de-identification; study protocol, statistical analysis plan and statistical code (including data dictionary)
Data available: No

Additional Information
Explanation for why data not available: Access to the data supporting this study's findings (participant-level data, study protocol, datasets, and statistical code) is available from the corresponding author upon reasonable request. The data are not publicly available due to privacy or ethical restrictions.
